# Exploring Andean High-Altitude Lake Extremophiles through Advanced Proteotyping

**DOI:** 10.1021/acs.jproteome.3c00538

**Published:** 2024-02-20

**Authors:** Katharina Runzheimer, Clément Lozano, Diana Boy, Jens Boy, Roberto Godoy, Francisco J. Matus, Denise Engel, Bruno Pavletic, Stefan Leuko, Jean Armengaud, Ralf Moeller

**Affiliations:** † Department of Radiation Biology, Institute of Aerospace Medicine, German Aerospace Center (DLR), 51147 Cologne, Germany; ‡ Département Médicaments et Technologies pour la Santé (DMTS), CEA, INRAE, SPI, Université, Paris-Saclay, F-30200 Bagnols-sur-Cèze, France; § Institute of Microbiology, Leibniz University Hannover, 30419 Hannover, Germany; ∥ Institute of Soil Science, Leibniz University Hannover, 30419 Hannover, Germany; ⊥ Instituto de Ciencias Ambientales y Evolutivas, Universidad Austral de Chile, 509000 Valdivia, Chile; # Laboratory of Conservation and Dynamics of Volcanic Soils, Department of Chemical Sciences and Natural Resources, Universidad de La Frontera, 4811230 Temuco, Chile; ∇ Network for Extreme Environmental Research (NEXER), Universidad de La Frontera, 4811230 Temuco, Chile

**Keywords:** tandem mass spectrometry proteotyping, Atacama Desert, Altiplano, high-altitude Andean lakes, extremophiles, halophiles

## Abstract

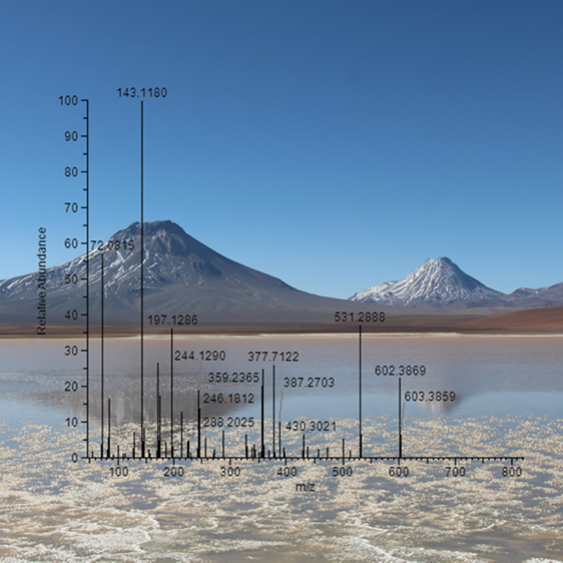

Quickly identifying and characterizing isolates from extreme environments is currently challenging while very important to explore the Earth′s biodiversity. As these isolates may, in principle, be distantly related to known species, techniques are needed to reliably identify the branch of life to which they belong. Proteotyping these environmental isolates by tandem mass spectrometry offers a rapid and cost-effective option for their identification using their peptide profiles. In this study, we document the first high-throughput proteotyping approach for environmental extremophilic and halophilic isolates. Microorganisms were isolated from samples originating from high-altitude Andean lakes (3700–4300 m a.s.l.) in the Chilean Altiplano, which represent environments on Earth that resemble conditions on other planets. A total of 66 microorganisms were cultivated and identified by proteotyping and 16S rRNA gene amplicon sequencing. Both the approaches revealed the same genus identification for all isolates except for three isolates possibly representing not yet taxonomically characterized organisms based on their peptidomes. Proteotyping was able to indicate the presence of two potentially new genera from the families of *Paracoccaceae* and *Chromatiaceae/Alteromonadaceae*, which have been overlooked by 16S rRNA amplicon sequencing approach only. The paper highlights that proteotyping has the potential to discover undescribed microorganisms from extreme environments.

## Introduction

### Atacama Desert and High-Altitude Andean Lakes

Extremophile environments harbor an incommensurable diversity of yet undiscovered microorganisms. The Atacama Desert and its adherent Puna de Atacama in South America are perfect examples of such an environment, combining high-altitude (up to >6000 m a.s.l.), high UV radiation, aridity, and hypersaline lakes.^1−3^ These conditions, combined with various geomorphological patterns, including coastal and Andean salt flats, rivers, lagoons, and geothermal fields among others, create a remarkable ecosystem. A common feature is the presence of naturally high arsenic concentrations as a result of leaching and weathering of rocks and mineral strata which cause the arsenic to reach water bodies such as salt flats, lagoons, and rivers. In addition, the effect of evaporation increases the concentration of the metalloid along with other mineral salts.^4^ While the Atacama region has long been considered hostile to life, recent studies have demonstrated the immense diversity of microorganisms, which are able to survive these extreme conditions.^5,6^ Nevertheless, further research is needed to comprehensively document the wide diversity and function of this ecosystem,^7^ particularly in view of the recent scenario with significant increase in mining operations and their environmental impacts.^8,9^

The Andean sea lakes in the high altitudes of South America, also known as high-altitude Andean lakes (HAAL) are considered to be environments on Earth that resemble conditions of early Mars (3.7–3.2. Ga ago).^10,11^ Since the most important parameter for prediction of Earth-like life on other celestial bodies is liquid water,^12^ these lakes on early Mars could be envisioned as favorable sites for life.^10^ In fact, lakes on early Mars and HAAL display striking similarities; thus research on stress factors in these terrestrial environments may foster the search for potential life on future missions to Mars.^10^

Organisms that have adapted to the habitats located in Chile include thermophiles, acidophiles, halophiles, alkaliphiles, xerotolerant and radioresistant bacteria, as well as psychrophiles.^5^ A study investigating the HAAL Laguna Lejia and Laguna en Salar de Aguas de Calientes showed the presence of cultivable *Firmicutes* and *Gammaproteobacteria* including genera of *Exiguobacterium, Halomonas, Serratia, Aeromonas,* and *Shewanella*. Isolates from these habitats revealed resistance toward UV radiation.^13^ Another study reported the existence of *Proteobacteria*, *Bacteroidetes,* and unclassified organisms as main components of the bacterial community within 0.5 and 4 m depth sediment samples from lake Licancabur.^10^ In addition, a study at the Salar de Maricunga highlighted the presence of highly interesting proteins of yet unidentified microorganisms that are resistant to oxidative stress induced by perchlorates, hydrogen peroxides, UV radiation, or other forms of oxidative stress. Some of the closest known proteins related to these functions belong to halophilic bacteria such as *Roseibaca ekhonensis* or *Halofilum ochraceum*.^14^ Perchlorates have been detected in relatively high amounts in Martian soils^15,16^ as well as in dry regions like the Atacama Desert^17^ and in its water bodies.^18^ Due to their hygroscopic properties and the ability to reduce the melting point, they could lead to a potential liquid habitat for halophilic prokaryotes on Mars. However, they display toxicity due to their chaotropic activity. Therefore, research on such perchlorate metabolisms, which are primarily found in microorganisms of hypersaline sites, enables a better understanding of possible life on Mars.^14^

### Techniques for Identification of Microorganisms, Their Strengths and Limitations

The pioneering work of Venter and his team during the large-scale Sorcerer II expedition have yielded a wealth of insights into the genetic information on diverse marine microorganisms.^19^ Meanwhile, the rise of culturomics, i.e., the systematic isolation of multiple isolates by assaying numerous culture conditions,^20,21^ has shown to be a valuable tool for the discovery of novel clinical and environmental isolates.^22^ The high number of isolates obtained from such methods requires fast, robust, and cost-effective identification techniques that lend themselves to high-throughput isolation approaches. The analysis of specific gene sequences or even the whole genome sequence can be further used for taxonomic classification. When proposing new microorganisms, the overall genome related index including the average nucleotide identity (ANI) and digital DNA–DNA hybridization (dDDH) values should be calculated between the newly discovered isolates and the related type strains. Thresholds of 98.7% (16S rRNA), 95–96% (ANI), and 70% (dDDH) have been proposed for defining new prokaryotic species.^23^

16S rRNA gene amplicon sequencing was and is the most widely used option in species identification but also has its drawbacks. Since the 16S rRNA gene consists of regions where sequences are highly conserved, one is sometimes not able to distinguish between several closely related bacterial species, leading to a wrong or imprecise identification. This is the case, for example, in the identification of *Pseudomonas*(24,25) and *Bacillus* species.^25,26^ In the case of *Pseudomonas*, other molecular markers such as *atpD, gyrB,* as well as *rpoB* and *rpoD* are used for multilocus sequence analysis to distinguish between different species.^24,27^ In recent years, whole-genome sequencing and other metagenomic analysis methods helped to overcome the shortcomings of 16S rRNA analysis, but there are still some obstacles to deal with, such as the accurate identification of unculturable microorganisms and the comprehensive analysis of functional traits.

Matrix-assisted laser desorption-ionization time-of-flight spectrometry is a reliable option for identification. However, it is limited to organisms that are referenced in a spectral database containing spectra from previously analyzed organisms and by low resolution.^28^ Therefore, it may not be suitable for environmental isolates or mixtures of microorganisms but can be useful for dereplicating identical isolates during culturomics.

Recently, tandem mass (MS/MS) spectrometry proteotyping has been documented as a competitive option to identify and characterize environmental isolates, as exemplified with marine isolates^29^ and biofilms from extreme environments.^30^ It utilizes the shotgun proteomics approach in which proteins are enzymatically cleaved into small peptides using trypsin. The resulting peptides are then separated by reverse-phase chromatography and sequenced by using MS/MS spectrometry. The taxonomic information associated with the peptide sequences can be analyzed to determine the presence of specific taxa in the sample. This is done by using taxon-specific peptide sequences at all taxonomical ranks.^31^ Since proteotyping is not limited to one gene or protein, results may be more discriminative than single-gene identification. Overall, proteotyping can offer a rapid, high-throughput, and cost-effective alternative for the identification of microbial isolates.^29^ Furthermore, the biological material required for this approach is advantageously low as shown by a recent study.^32^

### Aim of the Study

This study aims to comprehensively document the diversity and ecological roles of microorganisms in high-altitude Andean lakes. It is the first study examining extremophile isolates via MS/MS proteotyping. For this, we isolated and cultivated 66 organisms and conducted identification via 16S rRNA sequencing and MS/MS proteotyping. Results from both techniques were compared, and specific cases were discussed to better assess the strengths of proteotyping for the discovery and taxonomical classification of new isolates, even those far related to marker gene-sequenced organisms. In essence, the paper aims to explore the effectiveness of proteotyping in rapidly identifying and characterizing microorganisms from extreme environments with the potential to uncover novel species that might remain undetected using traditional DNA-based approaches.

## Materials and Methods

### Sampling and Cultivation

**Figure 1:**
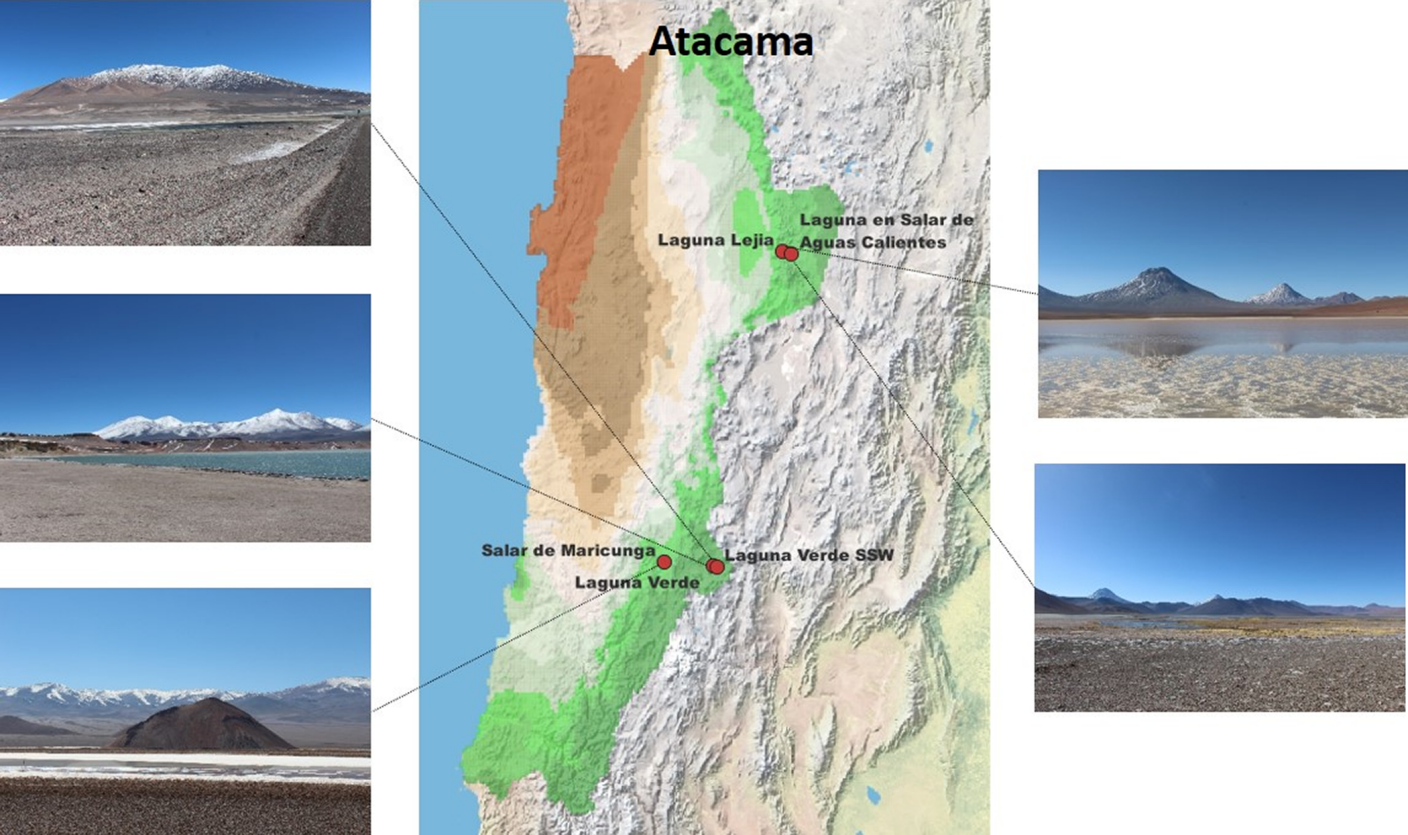
Sample locations in the Atacama in Chile. Remodified image from Boy et al.,^33^ with the inclusion of sampling locations. Image was created with QGIS.^34^ Rain distribution is indicated by color grading and corresponds to 0 (brown) to 155 mm p.a. (green).^33^

Sampling of five high-altitude lakes located in the Chilean Andes was conducted in March 2022 (Figure 1). For this, 1 L from each of the lakes Laguna Lejia (L. Lejia), Salar de Maricunga (S. d. Maricunga), Laguna Verde (L. Verde) and its side lake in the southwest (referred as L. Verde SSW), as well as Laguna en Salar de Aguas Calientes (L. Calientes) were sampled in a sterile manner, transported to the laboratory, and kept at 4 °C until further analysis. During sampling, GPS coordinates and the environmental conditions such as water temperature, surrounding temperature, geographic locations, and UV radiation were measured. In the laboratory, the chemical parameters of the water samples including the pH, electric conductivity, resistivity (RES), as well as salinity were determined (Table S1).

Aliquots of aquatic samples were used for the inoculation of different media in serial dilutions. Resulting colonies were picked and isolated on solid media, resulting in 66 unique isolates. For each isolate, DNA was extracted for 16S rRNA gene amplicon sequencing and subsequent identification. In parallel, proteins were subjected to a shotgun proteomic workflow, followed by a proteotyping-based identification as described in previous works.^29,35^

**Figure 2:**
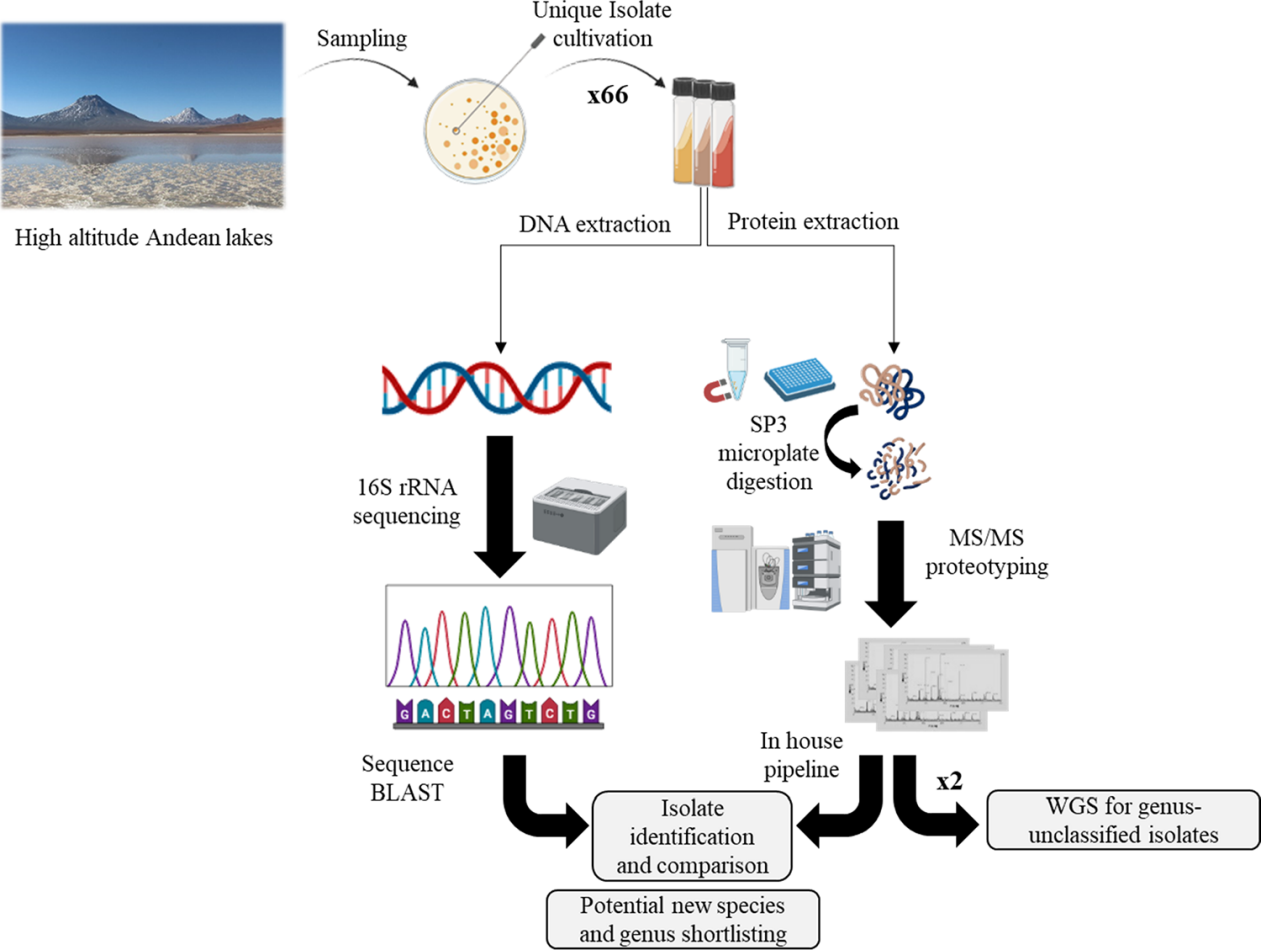
Overview of the experimental workflow. Samples were taken from five different HAAL, and microorganisms were cultivated with diverse techniques. Pure isolates were obtained and further analyzed according to their 16S rRNA sequence and peptide profile. For 16S rRNA sequencing, DNA was extracted, amplified, purified, and further sequenced and blasted against a nucleotide database provided by NCBI. For MS/MS proteotyping, isolates were cultivated in liquid matter, and proteins were digested with the SP3 microplate procedure. Peptides were analyzed with MS/MS and proteotyped according to an in-house pipeline. Figure was created with Biorender.com.

An overview of the experimental workflow is presented in Figure 2.

Cultivation was performed by using standard media for aquatic ecosystems [Reasoner 2A (R2A) from Teknova and Marine Broth (MB) from Difco] and was complemented by individual environmental-close cultivation using R2A media with modified pH and NaCl values as measured for this specific environment (see Table S1). One hundred μL to 1 mL of aquatic samples were spread undiluted and in several dilution series from 10^–1^ to 10^–4^ on agar plates and were incubated at room temperature (37 °C for halophile organisms) for 7 (to 12 for halophilic isolates) days to determine colony forming units (CFU/ml). To select for extremophile isolates, cultivation was also performed after X-ray treatment by exposing different volumes of original water samples to a final dose of 1000 (18.98 Gy/min for 53 min) and 4000 Gy (14.78 Gy/min for 300 min) with an X-ray system (Gulmay Medical Systems, Camberley, Surrey, United Kingdom). The shielding effect of an empty Eppendorf tube was considered within the calculation of the final dose. In addition, cultivation was performed on marine agar with pH 10 to select alkalitolerant species, marine agar with NaCl content of 15%, as well as artificial seawater media for halophiles (ASW, DSMZ medium J457) to screen for halophilic organisms. After counting and determination of the growth numbers in CFU/ml (*n* = 3), the plates were further incubated to acquire morphological unique isolates. To obtain pure cultures, colonies were picked and repeatedly restreaked on fresh media. Halophiles from hypersaline lakes S. d. Maricunga and L. Verde were isolated by cultivation on MB agar with 15% NaCl and counted after 12 days of incubation. Archaeal isolates were obtained from enrichment cultures in ASW media, followed by incubation and isolation from counting plates. The obtained isolates were further identified via 16S rRNA gene amplicon sequencing and MS/MS proteotyping. Supplementary data Table S2 provides information about the original cultivation conditions of each isolate.

### 16S rRNA Identification and Whole-Genome Sequencing

For 16S rRNA identification, colonies of every isolate were taken from freshly inoculated solid media to extract DNA according to the ZymoBIOMICS DNA Miniprep Kit protocol of Zymo Research. The concentration of the extracted DNA was measured with the fluorometric quantification device Qubit, and 16S rRNA was further amplified using the universal primers 27F and 1492R (bacteria)^36,37^ and Halo5F and Halo1462R (archaea).^38^ For amplification, 2–5 μL of DNA, 12.5 μL of 2× Q5Mastermix, and 2.5 μL of 10 μM of each primer were complemented with distilled, nuclease free water to a final volume of 25 μL. Bacterial amplification was initiated with a heating step of the lid to 110 °C, followed by a heating step at 98 °C for 30 s and a loop consisting of a denaturation step at 94 °C for 30 s, an annealing temperature of 57 °C for 45 s, and an elongation step at 72 °C for 90 s. The cycle was repeated 39 times and closed with a heating step at 72 °C for 2 min. For archaeal amplification, initial heating of the lid was performed as already described followed by a heating step at 95 °C for 3 min and cycles consisting of a denaturation step at 95 °C for 30 s, an annealing step at 55 °C for 30 s, and an elongation step at 72 °C for 1 min. The loop was closed after 35 repetitions and a final heating step at 72 °C for 3 min.

Two μL of each PCR sample was used to verify the product on 1% agarose gel, and the remaining part of the sample was further purified according to instructions of the purification kit Wizard SV Gel and PCR Clean-Up System obtained from Promega. The purified 16S rRNA gene amplicon was sequenced with forward primers 27F and Halo5F with the commercial Sanger Sequencing service by Seqlab and Eurofins. Sequences of an average length of 1000 bp were further manually analyzed with Chromas software and blasted via NCBI to identify the most closely related species. Results were reduced to sequences from type material. Selected isolates, which demonstrated discrepancies with the proteotyping results, showing a 16S rRNA gene sequence similarity to a next relative species below 98.7% and examples discussed within this paper, were sequenced full length with the above-mentioned reverse primers and aligned using the software MEGA11.^39^ 16S rRNA-based phylogenetic trees were generated with the data acquired from the NCBI database and further modified with MEGA11 (Version 11.0.13).^39^ The phylogenetic tree construction was conducted according to the maximum-likelihood and the Tamura-Nei model.^40^

Isolates that were genus-unclassified according to the proteotyping results and interpretation were further whole-genome sequenced. For this, the extracted DNA was analyzed using Oxford Nanopore technology with the NBK112.24 kit and an R9.16 flow cell. A concentration of 200 ng/μL DNA was used for the analysis. High accuracy base calling was achieved using the software Guppy (Version 6.4.6). The genome assembly was performed using Flye software (Version 2.9.2).^41^ Annotation of the assembled genomes was carried out using Prokka software (Version 1.14.6)^42^ through the web-based analysis platform Galaxy.^43^ From these data, predicted protein-coding-genes and the resulting proteins were used for amino acid-based phylogenetic trees, which were constructed with the alignment and phylogenetic tree construction software MEGA11 (Version 11.0.13)^39^ using the maximum-likelihood option and the Jones-Taylor-Thornton (JTT) model.^44^ The ANI was calculated via the online tool provided by EzBiocloud (ANI Calculator | Ezbiocloud.net).^45^

### MS/MS Proteotyping

For proteotyping, one colony of each unique isolate was picked in a sterile manner and transferred in a 15 mL tube with 7 mL of either R2A, MB, MB with NaCl added to 15%, TSB, or ASW. Cells were grown at room temperature as well as temperatures of 30 and 37 °C according to their individual optimal growth temperature. Cells were harvested after 1–12 days depending on their growth and varying among the isolates (for detailed information see Table S2). Cells from 2 mL of the liquid cultures were collected within a 2 mL Eppendorf tube by centrifugation for 15 min at 8000 *g*. Supernatants were discarded, and pellets were centrifuged again to remove all of the remaining liquid. Afterward, pellets were stored at −20 °C until further analysis.

For protein extraction, cell pellets were suspended in 200 μL of LDS buffer containing 106 mM Tris HCl, 141 mM Tris base, 2% LDS, 10% Glycerol, and 0.51 mM EDTA, supplemented with 5% beta-mercaptoethanol. Samples were incubated for 5 min at 99 °C in a thermomixer and sonicated for 5 min in an ultrasonic water bath. Samples were transferred into 0.5 mL screw cap microtubes containing 50 mg of a custom-made bead mixture.^46^ Bead beating was performed with a Precellys Evolution instrument at 10,000 rpm for 10 cycles of 30 s, with 30 s of pause between each cycle. Samples were centrifuged at 16,000 × *g* for 1 min, and supernatants were transferred to new microcentrifuge tubes before incubation at 99 °C for 5 min.

SP3 digestion was performed in a 96-well plate as previously described.^29^ Briefly, a 1:1 mix of hydrophilic (ref. GE65152105050250) and hydrophobic (ref. GE45152105050250) SpeedBeads magnetic beads was prepared at 50 mg/mL and stored at 4 °C until use. Protein reduction and alkylation were performed using 20 μL of protein solution, 4 μL of DTT at 35 mM, and 4 μL of iodoacetamide at 105 mM for 10 min in the dark at room temperature per replicate. A total of 200 μg of beads (4 μL) was added to the protein solution. A total of 200 μL of acetonitrile (85% final concentration) was added to induce protein aggregation. Bead–protein complexes were trapped using MagnaBind (Thermo Scientific). Supernatants were discarded, and proteins were washed twice with 200 μL of 70% ethanol and once with 180 μL of acetonitrile. Protein digestion was conducted at 50 °C for 30 min with 30 μL of digestion buffer containing 0.1 μg of Trypsin Gold in 50 mM NH_4_HCO_3_. Beads were trapped as described above, and the resulting peptides were pooled and acidified with 0.5% trifluoroacetic acid before LC-MS/MS analysis.

The resulting peptides were analyzed with a Q-Exactive HF (Thermo Scientific, Villebon sur Yvette, France) tandem mass spectrometer coupled to an ultimate 3000 nano-LC system (Thermo Scientific). Peptides were desalted on a reverse-phase PepMap 100 C18 μ-precolumn (5 mm, 100 Å, 300 mm i.d. × 5 mm, Thermo Scientific) and separated on a nanoscale PepMap 100 C18 nanoLC column (3 μm, 100 Å, 75 μm i.d. × 50 cm, Thermo Scientific) at a flow rate of 0.3 μL/min using a 23 min separation gradient (4% B from 0 to 3 min, 4–25% B from 3 to 20 min, and 25–32% B from 20 to 23 min) followed by 11 min washing (32–72% B from 23 to 24 min and 72% B from 24 to 34 min) and 15 min column equilibration (72–4% B from 35 to 50 min) of mobile phase A (0.1% HCOOH/100% H_2_O) and phase B (0.1% HCOOH/80% CH_3_CN).

The mass spectrometer was operated in a data-dependent acquisition mode with a Top20 strategy. Full-scan mass spectra were acquired from *m/z* 350 to *m*/*z* 1800. Only peptides with 2 or 3 positive charges were selected for fragmentation with a dynamic exclusion time of 10 s and an isolation window of *m*/*z* 1.6.

For each microbial isolate, the number of MS/MS spectra and the percentage of these spectra assigned to peptide sequences, known as peptide-to-spectrum matches (PSMs), were recorded. The number of MS/MS spectra depends on the amount of protein material extracted from each sample. Additionally, PSMs assigned to a taxon, referred to taxon-to-spectrum-matches (TSMs) in previous works,^29,47^ were determined. TSMs were attributed based on the sample’s peptidome to the closest organisms present in the database, ranging from the highest (superkingdom) to the lowest (species) taxonomical rank as previously described.^29,48^ Briefly, the proteotyping was conducted via an in-house-developed procedure consisting of a cascade search as follows: (1) 10,000 spectra were selected to run a Mascot search against the NCBInr database, reduced to one representative per species and totaling 94,176,939 protein sequences, 39,636,215,241 amino acids, and corresponding to 50,995 organisms (494 archaea, 2,231 eukaryota, 12,047 bacteria, and 36,223 viruses); (2) all spectra used for a Mascot query against a database were reduced to the genera previously identified during step 1 and all their descendants; (3) similarly, all spectra were searched against a database reduced to the species identified during step 2. Peptides were validated using a p-value below 0.3, 0.15, and 0.05 for steps 1, 2, and 3, respectively. Mascot searches were configured as follows: 3 ppm peptide tolerance during step 1, and 5 ppm peptide tolerance during steps 2 and 3, 0.02 Da MS/MS fragment tolerance, 2+ or 3+ peptide charges, a maximum of two missed cleavages, carbamidomethylation of cysteine as fixed modification, oxidation of methionine as variable modification, and trypsin as a proteolytic enzyme.

The percentage of TSM attribution at a specific taxonomical rank depends on the density and relatedness of reference genomes in the database that represent the analyzed isolate. A low percentage indicates that the isolate of interest has not been genome sequenced, or its genome is not yet included in the database used for interpretation. Specific peptides (spePEP), which are peptides unique to a particular taxonomic rank, were also considered. The number of taxon-specific peptides is influenced by the density of reference genomes in the corresponding branch of the tree of life and the molecular relatedness between closely related branches. As more genomes are sequenced, the number of species-specific peptides decreases. Identification at the species, genus, or family level can be determined based on the absolute number of TSMs under a given analytical condition. If similar numbers of TSMs and spePEP are shared among different closely related taxa, it indicates that the isolate possesses common characteristics among these taxa, and therefore, only the higher taxonomical rank should be considered valid. Functional characterization was performed as already described before.^29^

## Results and Discussion

### Environmental Parameters of the HAAL

For every HAAL, environmental parameters of each location including GPS data, water temperature, UV radiation, and water parameters including pH, conductivity, total dissolved solids (TDS), RES, salinity, and voltage were measured (Table S1).

Environmental parameters measured in this study correspond to several already reported characteristics, like high salt contents, of other HAAL.^3^ The lakes in this study, located at 3700–4300 m a.s.l., exhibited a neutral to alkaline pH and a wide range of salinity, reaching up to 27% with TDS values up to 217.3 g/L. This corresponds to a salt concentration approximately 8 times higher than that of seawater which contains 3.5% salt.^49^ The high salt concentrations are likely due to aridity, high solar energy leading to evaporation, and a negative water balance, resulting in increased TDS concentrations. S. d. Maricunga, which had the highest observed salt concentration, also exhibited a positive voltage, indicating the presence of oxidation and thus a more hostile environment. This could be attributed to high concentrations of perchlorate and nitrate, which have been reported in northern Chile.^18^

### Cultivation of Isolates from HAAL

Growth numbers from cultivation on different solid media and after different stress tests were counted and are presented in Table 1.

**Table 1:** Quantification of Growth Numbers in CFU/mL across the Five Different Lagoons^a^

	R2A	MB	modified R2A	1000 Gy, MB	4000 Gy, R2A	4000 Gy, modified R2A	MB, pH 10	MB, 15% NaCl
S. d. Maricunga	3.24 × 10^2^	2.94 × 10^2^	0	0	0	0	9.10 × 10^2^	1.10 × 10^2^
L. Verde	3.00 × 10^0^	4.48 × 10^2^	1.67 × 10^1^	0	0	0	0	0
L. Lejia	2.00 × 10^3^	9.20 × 10^5^	1.10 × 10^5^	4.60 × 10^3^	4.00 × 10^0^	2.00 × 10^0^	4.00 × 10^6^	7.00 × 10^1^
L. Verde SSW	1.70 × 10^5^	8.00 × 10^4^	3.40 × 10^5^	3.30 × 10^0^	0	0	5.00 × 10^4^	0
L. Calientes	3.00 × 10^5^	1.50 × 10^5^	3.50 × 10^5^	1.00 × 10^1^	0	0	2.00 × 10^4^	0

a Values reported as 0 indicate that no CFUs were observed. Modified R2A displays R2A media with modified pH and NaCl values as measured for this specific environment. Aquatic samples were spread on solid media (*n* = 3) following 7 days (12 days for MB with 15% NaCl) of incubation at room temperature.

Highest overall CFUs were found using MB and MB, pH 10 agar with L. Lejia revealing the highest CFU/ml. Modified R2A led to higher CFU/ml counts than unmodified R2A for L. Lejia, L. Verde SSW, and L. Calientes indicating the advantage of environmental-close cultivation conditions. Radiation treatment decreased the number of CFUs to 0 for S. d. Maricunga and L. Verde, while for L. Lejia, colonies were still detected after treatment with 4000 Gy, which is likely due to their higher initial growth numbers. Growth was detected for all HAAL on alkaline MB agar (pH 10) except L. Verde, and it was even higher than on neutral MB agar for S. d. Maricunga and L. Lejia. This finding matches to the measured neutral to alkaline pH values (Table S1).

### Panorama of Cultivated Isolates

Using the proteotyping approach, a total of 51 different species, 35 genera, 24 families, and 9 classes were identified among the 66 isolates. The 9 different classes are *Gammaproteobacteria* (27.27%), *Alphaproteobacteria* (16.67%), *Actinomycetia* (21.21%), *Bacilli* (19.70%), *Flavobacteriia* (4.55%), *Halobacteria* (3.03%), *Betaproteobacteria* (3.03%), *Cytophagia* (3.03%), and *Deinococci* (1.52%), spanning 6 phyla: *Bacteroidetes, Proteobacteria*, *Actinomycetota, Bacillota, Euryarchaeota*, and *Deinococcus*-*Thermus* (Table S2).

Previous studies have reported the presence of *Gammaproteobacteria* in L. Calientes,^13^ as well as *Proteobacteria* in general as an omnipresent population in sediments and aquatic habitats of Atacama,^10,50,51^ which we can confirm in this study. *Bacteroidetes* are also known to be abundant in most aquatic environments, including the HAAL of the Chilean Altiplano, and play an important role in these ecosystems by degrading high-molecular-weight compounds and enhancing algae growth.^50,52,53^ Several genes of microorganisms capable of metabolization, degradation, and protection from perchlorate related to genes from the genera *Roseibaca* spp. and *Marinobacter* spp. have been detected within the S. d. Maricunga.^14^ Same genera were also cultivated within the lakes L. Lejia, L. Verde, and S. d. Maricunga in this study. The latter revealed an oxidative environment according to its redox potential (Table S1) potentially promoting the abundancy of perchlorate-metabolizing and -resisting microorganisms. To our knowledge, this is the first study investigating the cultivable community within the lagoons L. Verde and its side lake and S. d. Maricunga located in Chile, thus preventing further comparison of the cultivable community.

### Identification with Proteotyping and 16S rRNA Amplicon Sequencing

From the 66 unique isolates exhibiting variations in morphology and microscopy, 22 isolates showed 16S rRNA gene identity percentages below 98.7% but above 95% (Table S2), indicating genus-level identification.^54^ This was also already observed within previous studies about HAAL that reported also a high number of unknown species relying on 16S rRNA gene analysis.^50,55^ In comparison, MS/MS proteotyping revealed the presence of 18 potentially new species and 2 potentially new genera based on the number of MS/MS spectra matches and shared peptides among taxa.

Overall, both 16S rRNA analysis and MS/MS proteotyping yielded consistent genus-level identification for all isolates, except for three isolates. Two of them were identified as potential new genera based on their peptide profiles (SM33 and SS13). It is worth noting that the limited availability of genome sequences currently hinders the identification of certain species through proteotyping. However, this limitation is expected to be addressed in the future as more genomes become available for newly described species. Out of the 33 isolates with discrepant identifications between proteotyping and 16S rRNA amplicon sequencing, 11 species lacked deposited genomes, thereby impeding identification through proteotyping (Table S2). Further statistical comparison of the concurrence of the results obtained by 16S rRNA sequencing and proteotyping is visualized within Table 2.

**Table 2:** Concurrence of Proteotyping and 16S rRNA Sequencing on the Species and Genus Level

	16S rRNA amplicon sequencing	tandem mass proteotyping
identification of same genus	63	
identification of same species	33	
potential new species	22 (<98.7% similarity)	18
potential new species according to both methods	8	
potential new genera	0 (<95% similarity)	2

On average, 11,531 MS/MS spectra were recorded, resulting in 4,633 PSMs representing an 39.9% attribution rate (Table S3). The ratio of TSMs attributed to a specific taxon as described in refs (29) and (47) ranged from 46.4 to 99.5% with an average of 90.7% at the genus level (TSMgenus/PSMall) and from 44.5 to 99.3% with an average of 78.5% at the species level (TSMspecies/PSMall). In a previous study on marine microorganisms from the NW Mediterranean Sea, a higher ratio at the species level (TSMspecies/PSMall) between 78.6 and 99.5% with an average of 96.4% was observed.^29^ This suggests that HAAL may contain a larger proportion of unsequenced proteins that could be of interest for biotechnological applications. Further details on the proteotyping statistics are presented in Table S3. Several examples of identification are presented in Table 3.

**Table 3:** Proteotyping and 16S rRNA-based Identification Results of the Isolates SS18, HP23, HR17, SM24, SM33, and SS13.^a^

sample ID	family	#TSMs	#spe PEPs	genus	#TSMs	#spePEPs	species	#TSMs	#spe PEPs	16S rRNA-based identification	16S rRNA similarity in %
SS18	*Rhodobacteraceae (reassigned to Roseobacteraceae and Paracoccaceae*)	5241	3820	*Paracoccus*	5241	3820	*Paracoccus aeridis*	5114	2484	*Paracoccus aeridis*	99.47
HP23	*Rhodobacteraceae (reassigned to Roseobacteraceae and Paracoccaceae*)	3111	1435	*Roseovarius*	2795	596	*Roseovarius tolerans*	1711	34	*Roseovarius tibetensis*	97.60
HR17	*Deinococcaceae*	3269	2317	*Deinococcus*	3269	2317	*Deinococcus metalli*	2243	138	*Deinococcus yunweiensis*	99.93
SM24	*unclassified Betaproteobacteria_family*	3830	739	*unclassified Betaproteobacteria_genus*	3830	739	*Betaproteobacteria bacterium HGW-Betaproteobacteria-16*	3679	713	*Hydrogenophaga palleronii*	98.57
SM33	*Rhodobacteraceae (reassigned to Roseobacteraceae and Paracoccaceae*)	5114	1909	*unclassified Rhodobacteraceae_genus*	3716	548	*Rhodobacteraceae bacterium EhC02*	3716	548	Seohaeicola saemankumensis	98.71
SS13	*Chromatiaceae*	3311	694	*Rheinheimera*	3187	351	*Rheinheimera pacifica*	2095	39	*Arsukibacterium ikkense*	98.28

a Presented are the sample ID and the best match according to proteotyping results consisting of identification of family, genus, and species level as well as its attributed TSMs and its spePEPs. In addition, closest relative species according to the 16S rRNA sequence with its similarity in % is given. Table S4 provides a comprehensive overview about further matches for every isolate.

### Concordant Proteotyping and 16S rRNA Identifications

A total of 46 samples were identified by the proteotyping approach with confidence at the species level, including sample SS18. For this isolate, a total of 10,509 MS/MS spectra were obtained, with 5,241 TSMs assigned at the genus level (Table 3), resulting in an assignment rate of 49.9%. Specifically, SS18 displayed 5,114 TSMs and 2484 spePEP attributed to *Paracoccus aeridis*, with no other matches within the database. This identification is supported by the fact that the database contained 128 genomes for the genus *Paracoccus*, thus providing high confidence. Similarly, 16S rRNA analysis also identified *Paracoccus aeridis* with a high confidence of 99.47% sequence similarity, exceeding the species boundary.

### Proteotyping Indicates Confidence at the Genus Rank for 18 Isolates

Proteotyping-based identification confidently assigned 18 samples to the genus level but revealed clear distances from currently sequenced species genomes. For example, sample HP23 displayed 2,795 TSMs and 596 spePEP associated with the *Roseovarius* genus (see Table 3). However, at the species level, 963–1711 TSMs and 6–48 spePEP were attributed to 13 different species within this genus, suggesting that HP23 represents either an unsequenced *Roseovarius* species not present in the database or a new, undescribed species (see Table S4). The assignment of TSMs at the genus level to 11,333 MS/MS spectra corresponded to a 24.7% attribution rate. Among the species, *Roseovarius tolerans* had the highest number of TSMs, while *Roseovarius nitratireducens* had the highest number of spePEP indicating a closer relationship to HP23. The strong decrease of TSMs assigned at the species level compared to the genus level is indicative of a low confidence for the species level due to the absence of a representative genome in the database.

Noteworthy, these findings align with the 16S rRNA identity percentage of 97.60%, which falls below the species-level confidence threshold. The 16S rRNA analysis indicated that the closest relatives of the isolates belonged to the genus *Roseovarius*, with *Roseovarius tibetensis* being the closest species. However, the proteotyping approach could not identify *Roseovarius tibetensis* due to the lack of its genome in the database used.

### Specific Cases Showed Discrepancies between Proteotyping and 16S rRNA Results

HR17 exhibited a high 16S rRNA identity percentage of 99.93% with *Deinococcus yunweiensis*, while proteotyping indicated similar TSMs and specific peptide numbers for two different *Deinococcus* species: *Deinococcus metallic* (2,243 TSMs and 138 spePEPs) and *Deinococcus* sp. KSM4–11 (2,198 TSMs and 143 spePEP) (Tables 3 and S4). These results suggest that the isolate shares sequence similarities with both species. The lack of information at the genome database level indicates that the correct species may not be represented yet. However, it is noteworthy that *Deinococcus* sp. KSM4–11 and *Deinococcus metallic* showed 94.5 and 93.9% similarities, respectively, to HR17 at the 16S rRNA gene sequence level. No hit was found for *Deinococcus yunweiensis* using the proteotyping approach, likely due to the absence of its genome in the database. The attribution rate at the genus level for *Deinococcus* based on 9,721 MS/MS spectra was 23.8%.

Other discrepancies were observed among three out of six *Pseudomonas* species, namely, LR4a, LR20, and SR29 (see Table S2). Although the 16S rRNA analysis indicated sequence alignments above the species threshold, the proteotyping results differed in these cases. However, for two of the three cases (LR20 and SR29), genome sequences of the closely related hits identified through 16S rRNA amplicon sequencing were available for MS/MS proteotyping analysis. The database for proteotyping consisted of a comprehensive set of 71 full genome sequences for the genus *Pseudomonas*. Utilizing protein-based identification on well-sequenced and extensively studied microorganisms proves to be beneficial as it is not limited to a single gene and provides greater discriminatory power. It is advantageous in cases where 16S rRNA sequencing is not enough for accurate identification, such as with species belonging to *Pseudomonas* and *Bacillus*,^24,26^ because of the lack of discriminative strength of this marker gene and the requirement of additional methods for species identification. Furthermore, proteotyping may be beneficial for identifying new species within halophilic organisms since a general polymorphism and discrepancy of 5% within the 16S rRNA sequence among halophilic organisms were previously observed.^56^ In order to utilize proteotyping as an effective method for identification, it is crucial to incorporate as many full genome sequences in public databases as possible as it would improve the accuracy of the identification and facilitate comprehensive genomic and protein-based comparisons.

### Proteotyping Indicates the Presence of Genus-Unclassified Organisms and Potential New Genera

Two samples, namely, SS13 and SM33, potentially belong to new genera based on the peptide profile and proteotyping interpretation. Moreover, proteotyping analysis revealed the presence of previously uncharacterized organisms (SM33 and SM24), which were exclusively detected through metagenomic studies.

Proteotyping analysis of isolate SM24 indicated a high similarity to the *Betaproteobacteria* bacterium HGW-*Betaproteobacteria*-16, an unclassified organism in the *Betaproteobacteria* group (Table 3), with a significant number of spePEP (713). This isolate shows some relatedness with the *Hydrogenophaga* genus with 445 genus spePEP (Table S4). SM24 and its HGW-16 relative may represent a new species within the *Hydrogenophaga* genus, which is currently represented only by metagenomic sequences. The 16S rRNA sequence analysis identified *Hydrogenophaga palleronii* as the most closely related sequence, with a similarity of 98.57%, slightly below the accepted species threshold of 98.7%. This species was not directly detected by proteotyping analysis despite the availability of its genome. Additionally, 16S rRNA analysis did not indicate the presence of the yet unclassified HGW-*Betaproteobacteria*-16, which is due to the absence of the sequence for the 16S rRNA gene within the genome.

However, the conserved protein rpoB’ derived from whole-genome sequencing of the *Betaproteobacteria* bacterium HGW-*Betaproteobacteria*-16 (Genbank assembly: GCA_002840835.1) detected within a metagenomic study^57^ exhibits its closest match to *Hydrogenophaga croceae* with a similarity of 87.98%.

**Figure 3:**
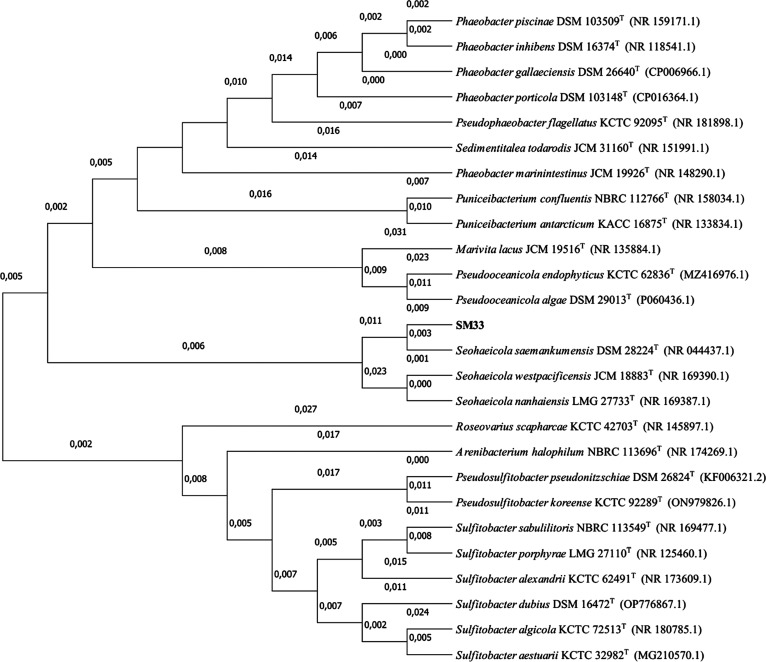
16S rRNA-based tree of SM33 and related sequences within the NCBI database. Numbers given at the branches indicate the amount of substitution per nucleotide position. Nucleotide tree is based on 1325 positions.

Isolate SM33 exhibited characteristics that suggested it could belong to a potential new genus based on the proteotyping approach. However, 16S rRNA amplicon sequencing showed a close relationship to *Seohaeicola saemankumensis* with a concordance of 98.71%, indicating that it is above the threshold for being a potential novel species. Further 16S rRNA analysis identified other genera, including *Phaeobacter, Pseudophaeobacter, Roseovarius, Arenibacterium, Sulfitobacter, Pseudooceanicola, Marivita, Puniceibacterium,* and *Sedimentitalea* (see Figure 3). The expanded analysis, incorporating data from uncultured/environmental sample sequences, indicated that this branch of the tree of life could be further delineated at the genus level with the incorporation of new isolates.

Proteotyping results revealed a significant number of spePEP belonging to an unclassified *Rhodobacteraceae* strain EhCO2 (Table 3), which could not be identified with 16S rRNA sequencing due to the absence of the gene within its available genome (RefSeq: GCF_001650895.1). Its genome revealed sequence similarities of 83–85% to several genera including *Marinibacterium, Ruegeria, Pukyongiella, Leisingera, Sulfitobacter, Roseovarius,* and *Pseudooceanicola* for the highly conserved gene *rpob*.

The ratio of genus-TSMs/recorded MS/MS spectra (20,713) was 17.9% (3,716 TSMs, unclassified *Rhodobacteraceae*_genus), 10.9% (2,252 TSMs, *Pseudooceanicola*), and 9.7% (2,009 TSMs, *Lutimaribacter*) for the top three genera (Table S4). Since *Seohaeicola saemankumensis* was not included in the proteotyping database, it could not be detected through this method. However, another species within the same genus (*Seohaeicola zhoushanensis*), which was available within the genomic database, was also not detected, indicating that SM33 represents an uncharacterized and unclassified organism within the *Roseobacteraceae* family. There were detections of taxon-specific peptides for 12 other genera within the same family (including for example*Pseudooceanicola, Lutimaribacter, Sulfitobacter, and Litorimicrobium*). Based on the detection of spePEP for an unclassified *Rhodobacteriaceae* genus and other genera within the family, it is presumed that SM33 may belong to an uncharacterized genus within the *Roseobacteraceae* family.

Proteotyping can be valuable in scenarios involving unclassified organisms sequenced through metagenomic studies that have not been included in 16S rRNA databases like NCBI. Isolate SM33 was placed within the same family based on both 16S rRNA sequencing and proteotyping analysis, but different information from available databases were used to identify the genus and species. These findings align with previous works highlighting the limited discriminating power of 16S rRNA-based identification^25,58^ and the relevance of whole genome-based taxonomical assignation.^59^

The SS13 isolate (see Tables 3 and S4) exhibited 694 and 169 order-specific peptides assigned to *Chromatiales* and *Alteromonadales,* respectively. These two orders belong to the same class, indicating that the isolate shares specific sequences with both orders. At the order level, there were 3,311 TSMs related to *Chromatiales* and 2,459 TSMs related to *Alteromonadales* out of a total of 3,810 TSMs at the class level. At the family level, taxon-specific peptides suggested similarities with *Chromatiaceae* and *Alteromonadaceae*, with 694 and 139 family-specific peptides, respectively. At the genus level, it showed relatedness to *Rheinheimera, Arsukibacterium*, and *Alishewanella* with 351, 49, and 108 genus-specific peptides, respectively. The ratio of genus-TSMs/recorded MS/MS spectra (14,637) was 21.8% (3,187 TSMs, *Rheinheimera*), 15.5% (2,269 TSMs, *Arsukibacterium*), and 14.9% (2,181, *Alishewanella*).

**Figure 4:**
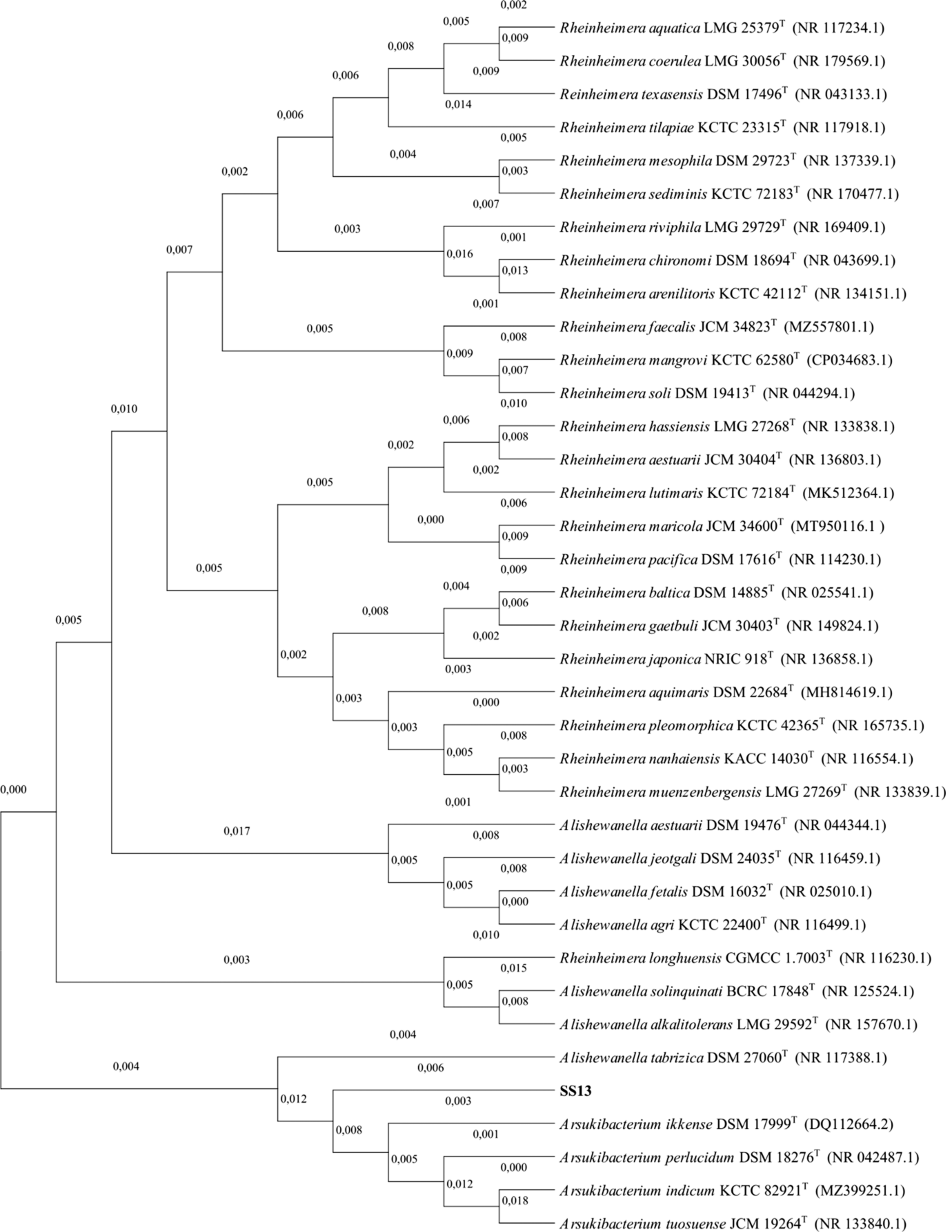
16S rRNA-based phylogenetic tree of SS13 and the closest sequences within the NCBI database. The numbers on the branches represent the substitutions per nucleotide position. In total, 1341 basepairs were compared.

These results suggest that the isolate is likely not well-represented in the database and belongs to a new genus that shares molecular sequences with the families *Chromatiaceae*, *Alteromonadaceae* and the genera *Rheinheimera*, *Arsukibacterium*, and *Alishewanella*. The significant decrease in taxon-specific peptides between family and genus levels indicates a lack of confidence in genus identification. The presence of spePEP in relatively low quantities across multiple species belonging to the three possible genera further supports the hypothesis of a new genus. However, the 16S rRNA gene identity of 98.28% with *Arsukibacterium ikkense*, the closest relative species, suggests an unreliable species-level identification as the threshold is 98.7%.^23^ The 16S rRNA phylogenetic tree, based on 1,341 base pairs, shows a distinct branch comprising *Arsukibacterium* and the SS13 isolate, which is separated from *Rheinheimera* (see Figure 4). However, proteotyping revealed the highest similarity of peptides from SS13 to the genus *Rheinheimera*. This discrepancy between 16S rRNA gene amplicon sequencing and MS/MS spectrometry proteotyping is of high interest as the discovery of this new genus and additional isolates could contribute to improving the taxonomy of this specific class.

**Figure 5:**
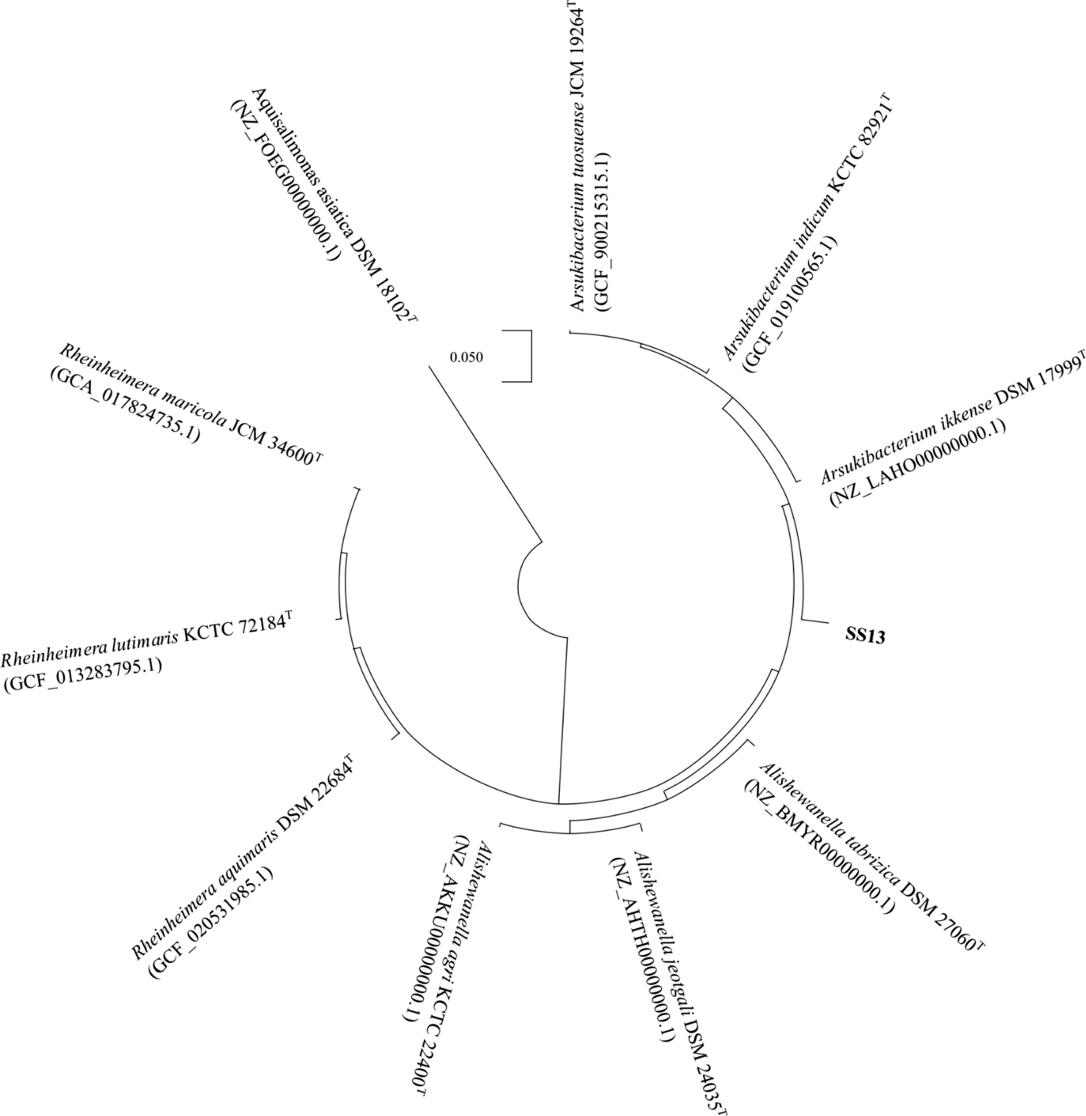
Phylogenetic reconstruction based on MLSA of specific conserved proteins: rpoA, rpoB’, rpoB, secY, and chaperonin GroEL. The final data set consisted of a total of 3,768 positions. *Aquisalimonas asiatica* DSM 18102^T^ was used as the rooted outgroup. To classify SS13, three different species from three different genera were included. The scale bar represents 0.050 substitutions per position.

Since this isolate displayed similarity to two different families according to both methods, its genome was used to further unravel the isolate’s taxonomy. To provide a comprehensive taxonomic overview, the genome of SS13 was compared to other highly conserved proteins across kingdoms, as previously demonstrated.^60^ A multilocus sequence analysis (MLSA) was conducted by concatenating amino acid sequences of specific conserved proteins: DNA-directed RNA polymerase subunit alpha (rpoA), DNA-directed RNA polymerase subunit beta (rpoB), isoform of DNA-directed RNA polymerase subunit beta’ (rpoB’), preprotein translocase subunit SecY (secY), and the chaperonin GroEL, which was the second most abundant protein detected through proteomics interpretation with the genome sequence. These proteins were compared across three different species in three different genera from the families *Chromatiaceae* and *Alteromonadaceae*. In total, 3,768 amino acid positions were included and compared in the final data set. The resulting phylogenetic tree (see Figure 5) suggests that SS13 may represent an organism positioned between the two genera *Arsukibacterium* and *Alishewanella*, belonging to the *Chromatiaceae* and *Alteromonadaceae* families, respectively, further supporting the hypothesis of a new genus.

Furthermore, the ANI was calculated between SS13 and representative species from the three different genera. The ANI values were significantly below the species threshold of 95–96%.^23^ Specifically, the ANI was 73.22% between SS13 and *Arsukibacterium ikkense* (RefSeq: NZ_LAHO00000000.1), 73.40% between SS13 and *Rheinheimera maricola* (RefSeq: GCA_017824735.1), and 71.89% between SS13 and *Alishewanella tabrizica* (RefSeq: NZ_BMYR00000000.1). Additionally, the analysis revealed a similar low relation (70.66%) between the species *Rheinheimera maricola* and *Arsukibacterium ikkense*.

Overall, the proteotyping results suggested the presence of two potential new genera. In this study, we demonstrated that a microorganism with a 16S rRNA identity percentage above 95% can still represent a new genus, as previously discussed.^54^

### Proteotyping Delivers Preliminary Functional Characterization

As samples for proteotyping were prepared via a shotgun proteomics workflow from cells grown in a specific condition, a preliminary functional characterization can be obtained.^29^

For example, isolate SS13, identified as a potential new genus with the closest relative *Rheinheimera pacifica* through MS/MS proteotyping, exhibited 2,663 PSMs belonging to 541 different protein groups when using a genus-specific (*Rheinheimera*) pan-database for the query (Table S5). As expected, a higher number of protein groups and PSMs could be identified (1045 and 7487, respectively) when the annotated genome could be obtained and was used as a query for the MS/MS spectra data set (Table S6), reinforcing the fact that the isolate is quite atypical compared to the hitherto annotated genomes present in the database.

For this isolate, the peptide profile provided insights into the presence of proteins associated with housekeeping functions. Specifically, *rpoA* was related to *Alkalimonas*, *rpoB* was linked to the genera *Rheinheimera* and *Alishewanella*, *rpoB*’ was associated with *Arsukibacterium*, *Alishewanella*, and *Rheinheimera*, and *secY* was related to *Rheinheimera* and *Arsukibacterium*. The peptide profile also revealed the presence of chaperones, with the chaperonin GroEL being the most abundant protein, indicating suboptimal cultivation conditions (Tables S5 and S6). This calls for refined cultivation methods for at least this isolate and further comparative proteomics studies to understand the molecular specificities of the isolates.

The protein GroEL displayed a similarity to chaperones from various genera, including *Rheinheimera, Bowmanella, Desulfovibrio*, and *Alishewanella*. In addition, a variety of other chaperones such as chaperone protein DnaJ, DnaK, HtpG, HtpG, Skp, SurA, ClpB, and RNA chaperone ProQ could be detected. Peptide analysis identified enzymes involved in the synthesis of compatible solutes (Proline-tRNA ligase, alpha, and alpha-trehalose phosphorylase) and proteins aiding in adaptation to high salt concentrations (osmotically inducible protein Y). Additionally, antioxidative enzymes (superoxide dismutase [Fe], glutathione amide-dependent peroxidase, organic hydroperoxide resistance protein OhrB, superoxide dismutase [Cu–Zn], and peroxiredoxin OsmC) were detected, playing a crucial role in protecting against oxidative stress.^61^ Furthermore, proteins have been observed that aid in various environmental conditions such as cold stress (cold shock-like protein CspA), starvation (DNA protection during starvation protein, stringent starvation protein A), or resistance to toxins (multidrug resistance protein MdtF and MexB) and phages (phage shock protein A, CRISPR-associated protein Csy3). MS/MS proteotyping also identified peptides related to a flagellar motor switch protein FliN, flagellin FliC, and chemotaxis proteins CheA and CheW in the genus *Rheinheimera*, indicating that SS13 is a motile microorganism. Microscopic observations confirmed this prediction in the laboratory.

## Conclusions

Sampling campaigns in poorly characterized extremophile environments often yield numerous isolates. We demonstrated that proteotyping was a suitable option to dereplicate the complexity of 66 isolates from five diverse HAAL. Proteotyping facilitates the selection of promising isolates for subsequent sequencing efforts while highlighting the gaps in the database and literature, permitting a time- and cost-effective approach.

One compelling outcome of this study is the consistent genus-level identification achieved through MS/MS proteotyping when compared to 16S rRNA amplicon sequencing across the isolate set and the concurrence on the species-level for extensively studied microorganisms. However, utilizing protein-based identification on well-sequenced and intensively researched microorganisms proves to be beneficial as it is not limited to a single gene and provides greater discriminatory power.

In contrast, when faced with poorly characterized and unsequenced organisms, discrepancies in the results emerged. Proteotyping not only identifies but also unveils potential new genera and species that were overlooked by single 16S rRNA gene analysis, emphasizing its superiority over 16S rRNA sequencing in characterizing microbial isolates. In addition, the study has shown that proteotyping extends beyond mere taxonomic identification, providing preliminary insights into potential adaptations and characteristics.

The distinctive taxonomic characteristics exhibited by isolate SS13 and presented within this study challenge conventional classification methods. This discovery holds implications for both our understanding of microbial diversity in extreme environments and the refinement of taxonomic approaches within this specific microbial class.

Proteotyping has emerged as a valuable tool in the fields of microbial ecology, taxonomy, and astrobiology. It proves to be particularly effective for characterizing microbial isolates, especially those that are well-suited for extremophiles and environmental samples, shedding light on their distinct properties and attributes.

## Data Availability

The mass spectrometry proteomics data have been deposited at the ProteomeXchange Consortium via the PRIDE partner repository with the data set identifier PXD044759 and 10.6019/PXD044759. The 16S rRNA data are stored within the NCBI GenBank with submission numbers OR475702-OR475748 and OR726321-OR726339.

## Supporting Information Available

Information on the next relative species according to the 16S rRNA and its similarity; best hit within the tandem mass proteotyping approach; and original habitat and original cultivation condition for isolation as well as the incubation time and media used for tandem mass proteotyping of every isolate used within this paper (XLSX)
Environmental parameters of the sample locations and chemical properties of water; summary statistics for the proteotyping interpretation of isolates; proteotyping and 16S rRNA-based identification results of the isolates SS18, HP23, HR17, SM24, SM33, and SS13; list of proteins identified for SS13 isolate using a pan-proteomics database for the genus *Rheinheimera;* and list of proteins identified for SS13 isolate using the database created from whole-genome sequencing (PDF)
