# Supplementary material for: Exploring Andean High-Altitude Lake Extremophiles through Advanced Proteotyping
Source: J Proteome Res. 2024 Feb 20;23(3):891–904. doi: 10.1021/acs.jproteome.3c00538 (PMC10913102; doi:10.1021/acs.jproteome.3c00538)
Supplement: Supplementary file 2 — pr3c00538_si_002.pdf [file pr3c00538_si_002.pdf]

# Exploring Andean High-Altitude Lake Extremophiles through Advanced Proteotyping

*Katharina Runzheimer[1], Clément Lozano[2], Diana Boy[3], Jens Boy[4] Roberto Godoy[5], Francisco J. Matus[6,7], Denise Engel [1] Bruno Pavletic[1], Stefan Leuko[1], Jean Armengaud[2] and Ralf Moeller[1*]*

*ralf.moeller@dlr.de

[1]Department of Radiation Biology, Institute of Aerospace Medicine, German Aerospace Center (DLR), 51147 Cologne, Germany, [2]Département Médicaments et Technologies pour la Santé (DMTS), Université, Paris-Saclay, CEA, INRAE, SPI, F-30200 Bagnols-sur-Cèze, France, [3]Institute of Microbiology, Leibniz University Hannover, 30419 Hannover, Germany, [4]Institute of Soil Science, Leibniz University Hannover, 30419 Hannover, Germany, [5]Instituto de Ciencias Ambientales y Evolutivas, Universidad Austral de Chile, 509000 Valdivia, Chile,[6]Laboratory of Conservation and Dynamics of Volcanic Soils, Department of Chemical Sciences and Natural Resources, Universidad de La Frontera, 4811230 Temuco, Chile, [7]Network for Extreme Environmental Research (NEXER), Universidad de La Frontera, 4811230 Temuco, Chile

Keywords: Tandem Mass Spectrometry Proteotyping, Atacama Desert, Altiplano, High-altitude Andean lakes, extremophiles, halophiles

# Table of Contents

**Table S1: Environmental parameters of the sample locations and chemical properties of the water.**

**Table S2: Information on next relative species according to the 16S rRNA and its similarity, the best hit within the tandem mass proteotyping approach, the original habitat and the original cultivation condition for isolation as well as the incubation time and media used for tandem mass proteotyping of every isolate used within this paper.**

**Table S3: Summary statistics for the proteotyping interpretation of isolates.**

**Table S4: Proteotyping and 16S rRNA based identification results of the isolates SS18, HP23, HR17, SM24, SM33 and SS13.**

**Table S5: List of proteins identified for SS13 isolate using a pan-proteomics database for the genus *Rheinheimera*.**

**Table S6: List of proteins identified for SS13 isolate using the database created from whole genome sequencing.**

***Table S1: Environmental parameters of the sample locations and chemical properties of the water.***

| | Altitude a.s.l. [m] | GPS coordinates | Day and Time of sampling | Water temperature | UV-radiation | pH | Conductivity | TDS | RES | Salinity | Redoxpotential |
|---|---|---|---|---|---|---|---|---|---|---|---|
| | | | | [°C] | [W/m²] | | [mS/cm] | (g/l) | [Ωcm] | [%] | [mV] |
| **L. Lejia** | 4295.5 | 23°30’26’’S; 67°41’36’’W | 31.03.2022, 18:00 | 18.4 | n.d. | 7.3 | 127.3 | 63.7 | 9.5E+00 | 7.7 | -15.0 |
| **L. Calientes** | 3991.4 | 23°31’57’’S; 67°35’03’’W | 31.03.2022, 17:30 | 24.9 | 5.3 | 7.6 | 4.2 | 2.1 | 2.4E+02 | 0.2 | -33.0 |
| **L. Verde** | 4335.4 | 26°53’24’’S; 68°28’58’’W | 20.03.2022, 18:00 | 10.4 | 6.6 | 8.0 | 173.7 | 136.9* | 5.8E+00 | 16.7* | -46.0 |
| **L. Verde SSW** | 4338.3 | 26°54’03’’S; 68°26’23’’W | 20.03.2022, 17:30 | 15.6 | 6.9 | 9.2 | 4.7 | 2.4 | 2.4E+02 | 0.3 | -121.5 |
| **S. d. Maricunga** | 3703.8 | 26°51’01’’S; 69°04’18’’W | 20.03.2022, 13:00 | 20.0 | 6.6 | 6.7 | 229.8 | 217.3* | 4.3E+00 | 27.9* | 20.5 |
| | | | | | | | | | | | |

***Note: For measurement of TDS and salinity, samples of Laguna Verde and Salar de Maricunga were diluted by 1:10 with distilled water as indicated with *. N.d. = not determined. TDS = total dissolved solids. RES = resistivity.***

| *Table S2: Information on next relative species according to the 16S rRNA and its similarity, the best hit within the tandem mass proteotyping approach, the original habitat and the original cultivation condition for isolation as well as the incubation time and media used for tandem mass proteotyping of every isolate used within this paper.* | | | | | |
|---|---|---|---|---|---|
| **Isolate** | **16S rRNA Result** | **16S rRNA Similarity (%)** | **Proteotyping Result** | **Original cultivation condition** | **Cultivation for Proteotyping** |
| **Isolates from Laguna de Aguas de Calientes** | | | | | |
| CS1 | *Pseudomonas borbori* | 99.72 | *Pseudomonas borbori* | Modified R2A, RT | 3 days, MB, RT |
| CS3* | *Sphingopyxis chilensis*** | 99.12 | *Sphingopyxis alaskensis* | Modified R2A, RT | 3 days, MB, RT |
| CS4* | *Pseudomonas anguilli-septica* | 99.69 | *Pseudomonas anguilliseptica* | Modified R2A, RT | 3 days, MB, RT |
| CS5* | *Variovorax boronicumulans* | 99.86 | *Variovorax paradoxus* | Modified R2A, RT | 3 days, MB, RT |
| CS6* | *Pseudomonas anguilliseptica* | 99.70 | *Pseudomonas anguilliseptica* | Modified R2A, RT | 3 days, MB, RT |
| CR10* | *Kocuria rhizophila* | 100.00 | *Kocuria rhizophila* | After 1000 Gy, R2A, RT | 6 days, R2A, RT |
| CM17 | *Micrococcus yunnanensis* | 99.71 | *Micrococcus luteus* | MB, RT | 3 days, MB, RT |
| **Isolates from Laguna Verde** | | | | | |
| HR3C* | *Erythrobacter donghaensis* | 99.26 | *Porphyrobacter dokdonensis (reassigned to Erythrobacter)* | R2A, RT | 3 days, R2A, RT |
| HM9* | *Psychroflexus sediminis* | 98.50 | *Psychroflexus tropicus* | MB, RT | 6 days, MB, RT |
| HM10 | *Idiomarina ramblicola* | 99.49 | *Idiomarina ramblicola* | MB, RT | 3 days, MB, RT |
| HM14a* | *Micrococcus yunnanensis* | 99.93 | *Micrococcus luteus* | MB, RT | 3 days, MB, RT |
| HM14b* | *Roseibaca ekhonensis* | 98.13 | *Roseibaca calidilacus* | MB, RT | 3 days, MB, RT |
| HR17* | *Deinococcus yunweiensis*** | 99.93 | *Deinococcus metalli/ Deinococcus sp. KSM4-11* | R2A, RT | 3 days, R2A, RT |

| | | | | | |
|---|---|---|---|---|---|
| HR20 | *Kocuria rhizophila* | 99.91 | *Kocuria rhizophila* | R2A, RT | 6 days, R2A, RT |
| HP23* | *Roseovarius tibetensis*** | 97.60 | *Roseovarius nitratireducens* | MB (pH10), RT | 3 days, MB, RT |
| **Isolates from Laguna Verde SSW** | | | | | |
| SR6* | *Rheinheimera soli*** | 99.28 | *Rheinheimera tangshanensis* | R2A, RT | 3 days, R2A, RT |
| SR8* | *Chryseoglobus indicus (reassigned to Microcella)* | 98.36 | *Chryseoglobus frigidaquae (basonym of Microcella frigidaquae)* | R2A, RT | 10 days, R2A, RT |
| SR9 | *Tabrizicola alkalilacus* | 99.33 | *Tabrizicola sp. DJC (Tabrizicola alkalilacus)* | R2A, RT | 1 day, TSB, RT |
| SS13* | *Arsukibacterium ikkense* | 98.28 | *Rheinheimera pacifica* | Modified R2A, RT | 3 days, MB, RT |
| SS18* | *Paracoccus aeridis* | 99.47 | *Paracoccus aeridis* | After 4000 Gy, Modified R2A, RT | 3 days, MB, RT |
| SM14* | *Belliella aquatica* | 98.97 | *Beliella aquatica* | MB, RT | 3 days, MB, RT |
| SM17a | *Micrococcus luteus* | 99.90 | *Micrococcus luteus* | After 1000 Gy, MB, RT | 3 days, MB, RT |
| SM17b* | *Marinisubtilis pacificus/Microcella indica* | 98.99 | *Chryseoglobus frigidaquae (basonym of Microcella frigidaquae)* | After 1000 Gy, MB, RT | 3 days, MB, RT |
| SM24* | *Hydrogenophaga palleronii* | 98.57 | *Betaproteobacteria bacterium HGW-Betaproteobacteria-16* | MB, RT | 3 days, MB, RT |
| SP26* | *Tabrizicola sediminis* | 98.30 | *Tabrizicola sp. DJC (Tabrizicola alkalilacus)* | MB, pH10, RT | 3 days, MB, RT |
| SP27* | *Mongoliitalea lutea* | 98.13 | *Mongoliitalea lutea* | MB, pH10, RT | 10 days, MB, RT |
| SP28* | *Yoonia vestfoldensis* | 98.86 | *Yoonia vestfoldensis* | MB, pH10, RT | 3 days, MB, RT |
| SR29* | *Pseudomonas cuatrocienegasensis* | 99.50 | *Pseudomonas borbori/ Pseudomonas sp. CC6-YY-74* | R2A, RT | 3 days, R2A, RT |
| SM33* | *Seohaeicola saemankumensis*** | 98.71 | *Rhodobacteraceae bacterium EhC02* | MB, RT | 3 days, MB, RT |

| | | | | | |
|---|---|---|---|---|---|
| SM34* | *Planococcus glaciei* | 98.24 | *Planomicrobium glaciei (reassigned to Planococcus)* | MB, RT | 3 days, MB, RT |
| SM100* | *Erythrobacter sanguineus* | 99.63 | *Porphyrobacter sp. AAP60 (reassgined to Erythrobacter)* | MB, RT | 6 days, MB, RT |
| SM101 | *Blastomonas aquatica* | 99.37 | *Blastomonas aquatica* | MB, RT | 6 days, MB, RT |
| **Isolates from Laguna Lejia** | | | | | |
| LR2* | *Kocuria oceani*** | 99.28 | *Kocuria turfanensis* | R2A, RT | 3 days, R2A, RT |
| LR4a* | *Pseudomonas arcuscaelestis*** | 100.00 | *Pseudomonas alkylphenolica* | R2A, RT | 3 days, R2A, RT |
| LR8 | *Nesterenkonia sandarakina* | 99.39 | *Nesterenkonia sandarakina* | R2A, RT | 5 days, TSB, 30°C |
| LR10 | *Exiguobacterium mexicanu m* | 99.49 | *Exiguobacterium mexicanum* | R2A, RT | 1 day, TSB, RT |
| LR14* | *Planococcus antarcticus* | 98.25 | *Planomicrobium soli (reassigned to Planococcus)* | R2A, RT | 2 day, TSB, 30°C |
| LR15* | *Planococcus massiliensis* | 98.11 | *Planococcus massiliensis* | R2A, RT | 1 day, TSB, 30°C |
| LR16 | *Microbacterium arborescens* | 99.71 | *Microbacterium arborescens* | R2A, RT | 6 days, R2A, RT |
| LR17 | *Marinilactibacillus piezotolerans* | 99.31 | *Marinilactibacillus piezotolerans* | R2A, RT | 5 days, TSB, 30°C |
| LS18a* | *Rhodococcus corynebacterioides* | 99.57 | *Rhodococcus trifolii* | Modified R2A, RT | 3 days, MB, RT |
| LS19* | *Halomonas azerica*** | 99.00 | *Halomonas sp. es.049* | Modified R2A, RT | 3 days, MB, RT |
| LR20* | *Pseudomonas songnenensis* | 99.36 | *Pseudomonas stutzeri* | R2A, RT | 3 days, R2A, RT |
| LS23 | *Planococcus salinarum* | 98.89 | *Planococcus salinarum* | Modified R2A, RT | 3 days, MB, RT |
| LM27* | *Planococcus koreensis* | 98.86 | *Planomicrobium koreense (reassigned to Planococcus koreensis)* | After 1000 Gy, R2A | 3 days, MB, RT |

| | | | | | |
|---|---|---|---|---|---|
| LM28 | *Micrococcus luteus* | 99.79 | *Micrococcus luteus* | MB, RT | 3 days, MB, RT |
| LM29 | *Citricoccus zhacaiensis* | 99.56 | *Citricoccus zhacaiensis* | Modified R2A, RT | 3 days, MB, RT |
| LR32* | *Kocuria rosea* | 99.57 | *Kocuria turfanensis* | After 4000 Gy, R2A, RT | 3 days, R2A, RT |
| LR34* | *Planococcus glaciei* | 98.30 | *Planomicrobium glaciei (reassigned to Planococcus)* | After 4000 Gy, R2A, RT | 1 day, TSB, RT |
| LR36 | *Planococcus okeanokoites* | 99.81 | *Planomicrobium okeanokoites (reassigned to Planococcus)* | After 4000 Gy, R2A, RT | 5 days, TSB, 30°C |
| LS39* | *Planococcus rifietoensis* | 99.58 | *Planococcus maitriensis* | After 4000 Gy, Modified R2A, RT | 3 days, MB, RT |
| LS40 | *Psychrobacter namhaensis* | 99.80 | *Psychrobacter namhaensis* | After 4000 Gy, Modified R2A, RT | 3 days, MB, RT |
| LM43* | *Planococcus maitriensis* | 100.00 | *Planococcus maitriensis* | After 1000 Gy, MB | 3 days, MB, RT |
| LM45* | *Marinobacter persicus* | 98.29 | *Marinobacter persicus* | After 1000 Gy, MB | 3 days, MB, RT |
| LM47 | *Halomonas subterranea* | 98.76 | *Halomonas subterranea* | After 1000 Gy, MB | 3 days, MB, RT |
| LM49* | *Salinicoccus amylolyticus*** | 98.58 | *Salinicoccus luteus* | After 1000 Gy, MB | 3 days, MB, RT |
| LP51* | *Halomonas boliviensis* | 98.13 | *Halomonas subterranea* | MB, pH10 | 6 days, MB, RT |
| LR58* | *Planococcus chinensis* | 99.79 | *Planomicrobium koreense (reassigned to Planococcus koreensis)* | R2A, RT | 1 day, TSB, 30°C |
| LS61* | *Psychroflexus montanilacus*** | 97.78 | *Psychroflexus tropicus* | Modified R2A, RT | 2 day, TSB, 30°C |
| **Isolates from Salar de Maricunga** | | | | | |
| MM8* | *Marinobacter confluentis* | 97.55 | *Marinobacter confluentis* | MB, RT | 3 days, MB, RT |
| MM9* | *Halomonas salicampi* | 98.37 | *Halomonas salicampi* | MB, RT | 3 days, MB, RT |
| MM11a* | *Psychroflexus curvus*** | 98.64 | *Psychroflexus sediminis* | MB, RT | 6 days, MB, RT |

| | | | | | |
|---|---|---|---|---|---|
| MN39* | *Halomonas salicampi* | 98.28 | *Halomonas salicampi* | MB (15% NaCl), RT | 3 days, MB (15% NaCl), RT |
| MN42* | *Idiomarina loihiensis* | 99.43 | *Idiomarina loihiensis/ Idiomarina ramblicola* | MB (15% NaCl), RT | 6 days, MB (15% NaCl), RT |
| MA43* | *Haloarcula sebkhae* | 96.80 | *Haloarcula japonica* | ASW, 37°C | 11 days, ASW, 37°C |
| MA44a* | *Halorubrum ezzemoulense* | 98.67 | *Halorubrum BOL3-1* | ASW, 37°C | 12 days, ASW, 37°C |
| | | | | | |
| ***Note: HAAL = High-altitude lake, RT = room temperature, ASW = Artificial Sea Water, MB = Marine Broth, R2A = Reasoners 2A. TSB = Tryptic Soy Broth. * indicates full length analysis of the 16S rRNA gene. ** indicates that no full genome representative of the species was available for tandem mass proteotyping.*** | | | | | |

***Table S3: Summary statistics for the proteotyping interpretation of isolates.*** *Included are the mean values and its standard deviation and the minimum and maximum value.*

| | **Spectra number** | **Number of attributed spectra (PSMall)** | **Ratio of PSMs (%)** | **Genus-TSMs (TSMgenus)** | **Ratio of TSMgenus (%)** | **Ratio of TSMgenus per PSMall (%)** | **Species-TSMs (TSMspecies)** | **Ratio of TSMspecies (%)** | **Ratio of TSMspecies per PSMall (%)** |
|---|---|---|---|---|---|---|---|---|---|
| **Mean** | 11531 | 4633 | 39.9 | 4321 | 36.7 | 90.7 | 3830 | 32.3 | 78.5 |
| **Standard Deviation** | 3041 | 1612 | 8.3 | 1754 | 10.5 | 10.3 | 1866 | 12.0 | 14.6 |
| **Min. Value** | 2166 | 827 | 25.6 | 384 | 17.7 | 46.4 | 368 | 13.3 | 44.5 |
| **Max. Value** | 20713 | 8714 | 56.1 | 8666 | 55.8 | 99.5 | 8649 | 55.5 | 99.3 |

***Table S4: Proteotyping and 16S rRNA based identification results of the isolates SS18, HP23, HR17, SM24, SM33 and SS13.*** *Presented are the sample ID and the matches according to proteotyping results consisting of identification of order, family, genus and species level as well as its attributed taxon-spectra matches (TSMs) and its specific peptides (spePEPs). In addition, closest relative species according to the 16S rRNA sequence with its similarity in % is given. For every isolate several hits within the proteotyping approach are displayed and arranged according to their order of peptide-based relation*

| Sample ID | ORDER | #TSMs | #spePEPs | FAMILY | #TSMs | #spePEPs | GENUS | #TSMs | #spePEPs | SPECIES | #TSMs | #spePEPs | 16S rRNA based Identification | 16S rRNA Similarity in % |
|---|---|---|---|---|---|---|---|---|---|---|---|---|---|---|
| SS18 | *Rhodobacterales* | 5241 | 3820 | *Rhodobacteraceae (reassigned to Roseobacteraceae and Paracoccaceae)* | 5241 | 3820 | *Paracoccus* | 5241 | 3820 | *Paracoccus aeridis* | 5114 | 2484 | *Paracoccus aeridis* | 99.47% |
| | | | | | | | *Paracoccus* | 5241 | 3820 | *Paracoccus salipaludis* | 1486 | 33 | | |
| | | | | | | | *Paracoccus* | 5241 | 3820 | *Paracoccus sphaerophysae* | 1390 | 15 | | |
| | | | | | | | *Paracoccus* | 5241 | 3820 | *Paracoccus chinensis* | 1345 | 11 | | |
| HP23 | *Rhodobacterales* | 3152 | 2361 | *Rhodobacteraceae (reassigned to Roseobacteraceae and Paracoccaceae)* | 3111 | 1435 | *Roseovarius* | 2795 | 596 | *Roseovarius tolerans* | 1711 | 34 | *Roseovarius tibetensis* | 97.60% |
| | | | | | | | *Roseovarius* | 2795 | 596 | *Roseovarius sp. A-2* | 1625 | 31 | | |
| | | | | | | | *Roseovarius* | 2795 | 596 | *Roseovarius nitratireducens* | 1508 | 48 | | |
| | | | | | | | *Roseovarius* | 2795 | 596 | *Roseovarius mucosus* | 1411 | 34 | | |
| | | | | | | | *Roseovarius* | 2795 | 596 | *Roseovarius azorensis* | 1339 | 21 | | |

| | | | | | | | | | | | | | | |
|---|---|---|---|---|---|---|---|---|---|---|---|---|---|---|
| | | | | | | | *Roseovarius* | 2795 | 596 | *Roseovarius marisflavi* | 1219 | 12 | | |
| | | | | | | | *Roseovarius* | 2795 | 596 | *Roseovarius pacificus* | 1104 | 12 | | |
| | | | | | | | *Roseovarius* | 2795 | 596 | *Roseovarius litoreus* | 1085 | 21 | | |
| | | | | | | | *Roseovarius* | 2795 | 596 | *Roseovarius sp. TE539* | 1082 | 20 | | |
| | | | | | | | *Roseovarius* | 2795 | 596 | *Roseovarius confluentis* | 1000 | 6 | | |
| | | | | | | | *Roseovarius* | 2795 | 596 | *Roseovarius faecimaris* | 981 | 11 | | |
| | | | | | | | *Roseovarius* | 2795 | 596 | *Roseovarius spongiae* | 967 | 14 | | |
| | | | | | | | *Roseovarius* | 2795 | 596 | *Roseovarius arcticus* | 963 | 18 | | |
| HR17 | *Deinococcales* | 3269 | 2317 | *Deinococcaceae* | 3269 | 2317 | *Deinococcus* | 3269 | 2317 | *Deinococcus metalli* | 2243 | 138 | *Deinococcus yunweiensis* | 99.93% |
| | | | | | | | *Deinococcus* | 3269 | 2317 | *Deinococcus sp. KSM4-11* | 2198 | 143 | | |
| | | | | | | | *Deinococcus* | 3269 | 2317 | *Deinococcus koreensis* | 1765 | 62 | | |
| | | | | | | | *Deinococcus* | 3269 | 2317 | *Deinococcus actinosclerus* | 1359 | 3 | | |
| | | | | | | | *Deinococcus* | 3269 | 2317 | *Deinococcus grandis* | 1348 | 1 | | |
| | | | | | | | *Deinococcus* | 3269 | 2317 | *Deinococcus knuensis* | 1314 | 3 | | |

| | | | | | | | | | | | | | |
|---|---|---|---|---|---|---|---|---|---|---|---|---|---|
| | | | | | | | *Deinococcus* | 3269 | 2317 | *Deinococcus aerophilus* | 1206 | 16 | | |
| | | | | | | | *Deinococcus* | 3269 | 2317 | *Deinococcus deserti* | 1174 | 12 | | |
| | | | | | | | *Deinococcus* | 3269 | 2317 | *Deinococcus humi* | 1162 | 15 | | |
| | | | | | | | *Deinococcus* | 3269 | 2317 | *Deinococcus sp. Leaf326* | 1123 | 17 | | |
| | | | | | | | *Deinococcus* | 3269 | 2317 | *Deinococcus aquatilis* | 1097 | 8 | | |
| | | | | | | | *Deinococcus* | 3269 | 2317 | *Deinococcus puniceus* | 1023 | 8 | | |
| | | | | | | | *Deinococcus* | 3269 | 2317 | *Deinococcus apachensis* | 957 | 10 | | |
| | | | | | | | *Deinococcus* | 3269 | 2317 | *Deinococcus aquiradiocola* | 690 | 9 | | |
| SM24 | *unclassified Betaproteo-bacteria_order* | 3830 | 739 | *unclassified Betaproteobacteria_family* | 3830 | 739 | *unclassified Betaproteo-bacteria_genus* | 3830 | 739 | *Betaproteo-bacteria bacterium HGW-Betaproteo-bacteria-16* | 3679 | 713 | *Hydrogeno-phaga palleronii* | 98.57 % |
| | | | | | | | | | | *Betaproteo-bacteria bacterium HGW-Betaproteo-bacteria-3* | 1129 | 15 | | |
| | *Burkholderiales* | 3408 | 483 | *Comamonadaceae* | 3408 | 483 | *Hydrogenophaga* | 3379 | 445 | *Hydrogenophaga taeniospiralis* | 2114 | 25 | | |
| | | | | | | | | | | *Hydrogenophaga sp. 2FB* | 2005 | 45 | | |

| | | | | | | | | | | | | | | |
|---|---|---|---|---|---|---|---|---|---|---|---|---|---|---|
| | | | | | | | | | | *Hydrogenophaga sp. A37* | 1927 | 31 | | |
| | | | | | | | | | | *Hydrogenophaga palleronii* | 1904 | 20 | | |
| | | | | | | | | | | *Hydrogenophaga pseudoflava* | 1734 | 11 | | |
| | | | | | | | | | | *Hydrogenophaga sp. PAMC20947* | 1710 | 16 | | |
| | | | | | | | | | | *Hydrogenophaga crassostreae* | 1668 | 16 | | |
| | | | | | | | | | | *Hydrogenophaga sp. BA0156* | 1526 | 22 | | |
| | | | | | | | | | | *Hydrogenophaga sp. IBVHS2* | 1479 | 21 | | |
| | | | | | | | *Acidovorax* | 959 | 6 | *Acidovorax cavernicola* | 959 | 6 | | |
| SM33 | *Rhodo-bacterales* | 5215 | 3302 | *Rhodobacteraceae (reassigned to Roseobacteraceae and Paracoccaceae)* | 5114 | 1909 | *unclassified Rhodobacteraceae _genus* | 3716 | 548 | *Rhodobacteraceae bacterium EhC02* | 3716 | 548 | *Seohaeicola saeman-kumensis* | 98.71% |
| | | | | | | | *Pseudooceanicola* | 2252 | 80 | *Pseudooceanicola flagellatus* | 1896 | 38 | | |
| | | | | | | | *Pseudooceanicola* | 2252 | 80 | *Pseudooceanicola atlanticus* | 1282 | 17 | | |
| | | | | | | | *Pseudooceanicola* | 2252 | 80 | *Pseudooceanicola sp. CAU 1508* | 1232 | 23 | | |
| | | | | | | | *Lutimaribacter* | 2009 | 56 | *Lutimaribacter saemankumensis* | 1594 | 23 | | |

| | | | | | | | | | | | | | |
|---|---|---|---|---|---|---|---|---|---|---|---|---|---|
| | | | | | | | *Lutimaribacter* | 2009 | 56 | *Lutimaribacter litoralis* | 1491 | 30 | | |
| | | | | | | | *Litorimicrobium* | 2009 | 43 | *Litorimicrobium taeanense* | 2009 | 43 | | |
| | | | | | | | *Sulfitobacter* | 1899 | 66 | *Sulfitobacter sabulilitoris* | 1420 | 17 | | |
| | | | | | | | *Sulfitobacter* | 1899 | 66 | *Sulfitobacter guttiformis* | 1314 | 25 | | |
| | | | | | | | *Sulfitobacter* | 1899 | 66 | *Sulfitobacter sp. SK012* | 1280 | 15 | | |
| | | | | | | | *Roseovarius* | 1758 | 43 | *Roseovarius azorensis* | 1418 | 22 | | |
| | | | | | | | *Roseovarius* | 1758 | 43 | *Roseovarius nanhaiticus* | 1315 | 21 | | |
| | | | | | | | *Oceanicola* | 1696 | 33 | *Oceanicola sp. HL-35* | 1696 | 33 | | |
| | | | | | | | *Sediminimonas* | 1547 | 22 | *Sediminimonas qiaohouensis* | 1547 | 22 | | |
| | | | | | | | *Rhodobacter* | 1453 | 32 | *Rhodobacter azotoformans* | 1142 | 6 | | |
| | | | | | | | *Rhodobacter* | 1453 | 32 | *Rhodobacter vinaykumarii* | 946 | 15 | | |
| | | | | | | | *Puniceibacterium* | 1334 | 25 | *Puniceibacterium confluentis* | 1334 | 25 | | |
| | | | | | | | *Ruegeria* | 1308 | 17 | *Ruegeria sediminis* | 1308 | 17 | | |
| | | | | | | | *Yoonia* | 1307 | 35 | *Yoonia vestfoldensis* | 1307 | 35 | | |

| | | | | | | | | | | | | | |
|---|---|---|---|---|---|---|---|---|---|---|---|---|---|
| | | | | | | | *Paracoccus* | 887 | 14 | *Paracoccus sp. I-41R45* | 887 | 14 | | |
| | | | | *unclassified Rhodobacterales_family* | 2201 | 77 | *unclassified Rhodobacterales _genus* | 2201 | 77 | *Rhodobacterales bacterium* | 2201 | 77 | | |
| SS13 | *Chromatiales* | 3311 | 694 | *Chromatiaceae* | 3311 | 694 | *Rheinheimera* | 3187 | 351 | *Rheinheimera pacifica* | 2095 | 39 | *Arsuki-bacterium ikkense* | 98.28% |
| | | | | | | | *Rheinheimera (reassigned to Arsukibacterium)* | 3187 | 351 | *Rheinheimera perlucida (reassigned to Arsukibacterium)* | 2084 | 12 | | |
| | | | | | | | *Rheinheimera (reassigned to Arsukibacterium)* | 3187 | 351 | *Rheinheimera tuosuensis (reassigned to Arsukibacterium)* | 2051 | 13 | | |
| | | | | | | | *Rheinheimera* | 3187 | 351 | *Rheinheimera pleomorphica* | 1985 | 16 | | |
| | | | | | | | *Rheinheimera* | 3187 | 351 | *Rheinheimera aquimaris* | 1913 | 14 | | |
| | | | | | | | *Rheinheimera* | 3187 | 351 | *Rheinheimera baltica* | 1886 | 21 | | |
| | | | | | | | *Rheinheimera* | 3187 | 351 | *Rheinheimera nanhaiensis* | 1857 | 9 | | |
| | | | | | | | *Rheinheimera* | 3187 | 351 | *Rheinheimera riviphila* | 1411 | 12 | | |
| | | | | | | | *Rheinheimera* | 3187 | 351 | *Rheinheimera salexigens* | 1362 | 22 | | |

| | | | | | | | | | | | | | |
|---|---|---|---|---|---|---|---|---|---|---|---|---|---|
| | | | | | | | *Rheinheimera* | 3187 | 351 | *Rheinheimera tangshanensis* | 1347 | 19 | | |
| | | | | | | | *Arsukibacterium* | 2269 | 49 | *Arsukibacterium ikkense* | 2076 | 21 | | |
| | | | | | | | *Arsukibacterium* | 2269 | 49 | *Arsukibacterium sp. MJ3* | 1881 | 25 | | |
| | | | | | | | *Pararheinheimera (reassigned to Rheinheimera)* | 1400 | 13 | *Paraheinheimera texasensis (reassigned to Rheinheimera)* | 1400 | 13 | | |
| | *Altero-monadales* | 2459 | 169 | *Alteromonadaceae* | 2302 | 139 | *Alishewanella* | 2181 | 108 | *Alishewanella tabrizica* | 1803 | 21 | | |
| | | | | | | | *Alishewanella* | 2181 | 108 | *Alishewanella longhuensis* | 1790 | 19 | | |
| | | | | | | | *Alishewanella* | 2181 | 108 | *Alishewanella aestuarii* | 1680 | 3 | | |

| *Table S5: List of proteins identified for SS13 isolate using a pan-proteomics database for the genus Rheinheimera.* | | | | | |
|---|---|---|---|---|---|
| **RAWfile** | ['Q14963_SS13.raw'] | | | | |
| **DATpath** | \\susa\Data-Mascot\20230614 | | | | |
| **DATfile** | F484198.dat | | | | |
| **DBs** | DB_CL_SS13_Rheinheimera | | | | |
| **DB fasta(s)** | C:/inetpub/mascot/sequence/DB_CL/DB_CL_SS13_Rheinheimera/current/DB_CL_SS13_Rheinheimera__SS13.fasta | | | | |
| **Number_of_spectra** | 14637 | | | | |
| **Mascot pvalue (FDR=0.0100)** | 0,0019 | | | | |
| **Number_of_PSMs** | 2663 | | | | |
| | | | | | |
| **Protein group** | **Accession** | **Description** | **Molecular Weight (Da)** | **Organism origin [Superkingdom]** | **Spectral count** |
| 1 | WP_019675910.1 | chaperonin GroEL [Rheinheimera perlucida] | 57529 | Rheinheimera perlucida [Bacteria] | 110 |
| 1 | WP_097111438.1 | chaperonin GroEL [Rheinheimera tuosuensis] | 57540 | Rheinheimera tuosuensis [Bacteria] | 5 |
| 1 | WP_092789658.1 | chaperonin GroEL [Rheinheimera pacifica] | 57593 | Rheinheimera pacifica;Gammaproteobacteria bacterium HGW-Gammaproteobacteria-15 [Bacteria] | 73 |
| 2 | WP_166839709.1 | elongation factor Tu [Rheinheimera pleomorphica] | 43480 | [] | 85 |
| 2 | WP_127700863.1 | elongation factor Tu [Rheinheimera riviphila] | 43155 | Rheinheimera sp. KYPC3 [Bacteria] | 5 |
| 2 | WP_097113087.1 | elongation factor Tu [Rheinheimera tuosuensis] | 43325 | Rheinheimera tuosuensis [Bacteria] | 1 |
| 3 | WP_068238206.1 | alanine dehydrogenase [Rheinheimera sp. EpRS3] | 39306 | Rheinheimera sp. EpRS3 [Bacteria] | 45 |
| 3 | WP_070049387.1 | alanine dehydrogenase [Rheinheimera salexigens] | 38885 | Rheinheimera salexigens [Bacteria] | 12 |
| 4 | WP_166838267.1 | F0F1 ATP synthase subunit alpha [Rheinheimera pleomorphica] | 55411 | [] | 42 |

| | | | | | |
|---|---|---|---|---|---|
| 4 | WP_053423948.1 | MULTISPECIES: F0F1 ATP synthase subunit alpha [Rheinheimera] | 55445 | Pararheinheimera tangshanensis;Rheinheimera sp. YQF-1;Rheinheimera sp. KL1 [Bacteria] | 2 |
| 4 | WP_068237054.1 | MULTISPECIES: F0F1 ATP synthase subunit alpha [Rheinheimera] | 55247 | Rheinheimera pacifica;Gammaproteobacteria bacterium HGW-Gammaproteobacteria-15;Rheinheimera sp. EpRS3 [Bacteria] | 3 |
| 4 | WP_134053426.1 | F0F1 ATP synthase subunit alpha [Rheinheimera aquimaris] | 55425 | Rheinheimera aquimaris [Bacteria] | 1 |
| 5 | WP_097113090.1 | DNA-directed RNA polymerase subunit beta [Rheinheimera tuosuensis] | 150439 | Rheinheimera tuosuensis [Bacteria] | 40 |
| 5 | WP_127700871.1 | DNA-directed RNA polymerase subunit beta [Rheinheimera riviphila] | 150350 | Rheinheimera sp. KYPC3 [Bacteria] | 2 |
| 6 | WP_132581821.1 | molecular chaperone DnaK [Rheinheimera sp. D18] | 68636 | Rheinheimera sp. D18 [Bacteria] | 40 |
| 6 | WP_097111825.1 | molecular chaperone DnaK [Rheinheimera tuosuensis] | 68945 | Rheinheimera tuosuensis [Bacteria] | 10 |
| 6 | WP_173501887.1 | molecular chaperone DnaK [Rheinheimera sp. YQF-2] | 68898 | [] | 4 |
| 6 | WP_070050234.1 | molecular chaperone DnaK [Rheinheimera salexigens] | 69095 | Rheinheimera salexigens [Bacteria] | 2 |
| 7 | WP_019677786.1 | DNA-directed RNA polymerase subunit beta' [Rheinheimera perlucida] | 154738 | Rheinheimera perlucida [Bacteria] | 40 |
| 7 | WP_008899790.1 | DNA-directed RNA polymerase subunit beta' [Rheinheimera sp. A13L] | 154591 | Rheinheimera sp. A13L [Bacteria] | 3 |
| 7 | WP_068231331.1 | DNA-directed RNA polymerase subunit beta' [Rheinheimera sp. EpRS3] | 154723 | Rheinheimera sp. EpRS3 [Bacteria] | 1 |
| 7 | WP_132586018.1 | DNA-directed RNA polymerase subunit beta' [Rheinheimera sp. D18] | 155069 | Rheinheimera sp. D18 [Bacteria] | 1 |
| 7 | WP_070047724.1 | DNA-directed RNA polymerase subunit beta' [Rheinheimera salexigens] | 154629 | Rheinheimera salexigens [Bacteria] | 1 |
| 8 | WP_173501494.1 | dihydrolipoyl dehydrogenase [Rheinheimera sp. YQF-2] | 50179 | [] | 33 |

| | | | | | |
|---|---|---|---|---|---|
| 8 | WP_019677757.1 | dihydrolipoyl dehydrogenase [Rheinheimera perlucida] | 50296 | Rheinheimera perlucida [Bacteria] | 4 |
| 8 | WP_166836789.1 | dihydrolipoyl dehydrogenase [Rheinheimera pleomorphica] | 50019 | [] | 1 |
| 8 | WP_008217546.1 | dihydrolipoyl dehydrogenase [Rheinheimera nanhaiensis] | 50090 | Rheinheimera nanhaiensis [Bacteria] | 1 |
| 9 | WP_097110509.1 | F0F1 ATP synthase subunit beta [Rheinheimera tuosuensis] | 50031 | Rheinheimera tuosuensis [Bacteria] | 32 |
| 9 | WP_134053422.1 | F0F1 ATP synthase subunit beta [Rheinheimera aquimaris] | 49915 | Rheinheimera aquimaris [Bacteria] | 5 |
| 10 | WP_092793421.1 | elongation factor G [Rheinheimera pacifica] | 77036 | Rheinheimera pacifica [Bacteria] | 30 |
| 10 | WP_008897760.1 | elongation factor G [Rheinheimera sp. A13L] | 77028 | Rheinheimera sp. A13L [Bacteria] | 4 |
| 10 | WP_173499813.1 | elongation factor G [Rheinheimera sp. YQF-2] | 77189 | [] | 3 |
| 11 | WP_026349125.1 | 30S ribosomal protein S1 [Rheinheimera perlucida] | 61774 | [] | 26 |
| 11 | WP_173500862.1 | 30S ribosomal protein S1 [Rheinheimera sp. YQF-2] | 61742 | [] | 3 |
| 11 | WP_147905460.1 | 30S ribosomal protein S1 [Rheinheimera tangshanensis] | 61547 | Pararheinheimera tangshanensis [Bacteria] | 1 |
| 12 | WP_008899096.1 | MULTISPECIES: DNA-directed RNA polymerase subunit alpha [unclassified Rheinheimera] | 36648 | Rheinheimera sp. A13L;Rheinheimera sp. YQF-1 [Bacteria] | 24 |
| 12 | WP_008224458.1 | MULTISPECIES: DNA-directed RNA polymerase subunit alpha [Rheinheimera] | 36628 | Rheinheimera nanhaiensis;Rheinheimera aquimaris [Bacteria] | 2 |
| 13 | WP_019677728.1 | MULTISPECIES: 50S ribosomal protein L5 [Chromatiaceae] | 20011 | Rheinheimera tuosuensis;Rheinheimera perlucida;Arsukibacterium ikkense [Bacteria] | 22 |
| 13 | WP_127688206.1 | 50S ribosomal protein L5 [Rheinheimera sp. YQF-1] | 20041 | Rheinheimera sp. YQF-1 [Bacteria] | 2 |
| 14 | WP_027670858.1 | pyruvate dehydrogenase (acetyl-transferring), homodimeric type [Rheinheimera baltica] | 99047 | Rheinheimera baltica [Bacteria] | 21 |
| 14 | WP_166836785.1 | pyruvate dehydrogenase (acetyl-transferring), homodimeric type [Rheinheimera pleomorphica] | 99285 | [] | 5 |

| | | | | | |
|---|---|---|---|---|---|
| 15 | WP_068230022.1 | 4-hydroxyphenylpyruvate dioxygenase [Rheinheimera sp. EpRS3] | 40532 | Rheinheimera sp. EpRS3 [Bacteria] | 20 |
| 15 | WP_127699189.1 | 4-hydroxyphenylpyruvate dioxygenase [Rheinheimera riviphila] | 40388 | Rheinheimera sp. KYPC3 [Bacteria] | 1 |
| 15 | KOO59033.1 | 4-hydroxyphenylpyruvate dioxygenase [Rheinheimera sp. KL1] | 40128 | [] | 1 |
| 16 | WP_068233188.1 | bifunctional aconitate hydratase 2/2-methylisocitrate dehydratase [Rheinheimera sp. EpRS3] | 93823 | Rheinheimera sp. EpRS3 [Bacteria] | 19 |
| 16 | WP_019675058.1 | bifunctional aconitate hydratase 2/2-methylisocitrate dehydratase [Rheinheimera perlucida] | 94023 | Rheinheimera perlucida [Bacteria] | 1 |
| 17 | WP_019677244.1 | peroxiredoxin [Rheinheimera perlucida] | 20795 | Rheinheimera perlucida [Bacteria] | 14 |
| 17 | ALZ74421.1 | alkyl hydroperoxide reductase [Rheinheimera sp. F8] | 20778 | Rheinheimera sp. F8 [Bacteria] | 7 |
| 17 | WP_132586254.1 | peroxiredoxin [Rheinheimera sp. D18] | 20733 | Rheinheimera sp. D18 [Bacteria] | 2 |
| 18 | WP_019675581.1 | citrate (Si)-synthase [Rheinheimera perlucida] | 48350 | Rheinheimera perlucida [Bacteria] | 18 |
| 18 | WP_134054460.1 | citrate synthase [Rheinheimera aquimaris] | 48229 | Rheinheimera aquimaris [Bacteria] | 3 |
| 19 | WP_127697480.1 | ADP-forming succinate--CoA ligase subunit beta [Rheinheimera riviphila] | 41851 | Rheinheimera sp. KYPC3 [Bacteria] | 18 |
| 19 | WP_070049109.1 | ADP-forming succinate--CoA ligase subunit beta [Rheinheimera salexigens] | 41877 | Rheinheimera salexigens [Bacteria] | 4 |
| 19 | WP_027670937.1 | ADP-forming succinate--CoA ligase subunit beta [Rheinheimera baltica] | 41308 | Rheinheimera baltica [Bacteria] | 1 |
| 20 | WP_097112053.1 | molecular chaperone HtpG [Rheinheimera tuosuensis] | 72694 | Rheinheimera tuosuensis [Bacteria] | 17 |
| 20 | WP_092796293.1 | molecular chaperone HtpG [Rheinheimera pacifica] | 72622 | Rheinheimera pacifica [Bacteria] | 6 |
| 20 | WP_173500934.1 | molecular chaperone HtpG [Rheinheimera sp. YQF-2] | 72625 | [] | 2 |
| 21 | WP_127697479.1 | succinate--CoA ligase subunit alpha [Rheinheimera riviphila] | 30402 | Rheinheimera sp. KYPC3 [Bacteria] | 17 |
| 22 | WP_186435071.1 | 2-oxoglutarate dehydrogenase complex dihydrolipoyllysine-residue succinyltransferase [Rheinheimera sp. YQF-1] | 42586 | [] | 16 |

| | | | | | |
|---|---|---|---|---|---|
| 22 | TXH93336.1 | 2-oxoglutarate dehydrogenase complex dihydrolipoyllysine-residue succinyltransferase [Rheinheimera sp.] | 42154 | Rheinheimera sp. [Bacteria] | 1 |
| 23 | WP_134059736.1 | 50S ribosomal protein L3 [Rheinheimera aquimaris] | 22183 | Rheinheimera aquimaris [Bacteria] | 16 |
| 23 | WP_019677716.1 | 50S ribosomal protein L3 [Rheinheimera perlucida] | 22345 | Rheinheimera perlucida [Bacteria] | 2 |
| 24 | WP_019674803.1 | endopeptidase La [Rheinheimera perlucida] | 88654 | Rheinheimera perlucida [Bacteria] | 15 |
| 24 | WP_173502184.1 | endopeptidase La [Rheinheimera sp. YQF-2] | 88990 | [] | 2 |
| 24 | TXH97250.1 | endopeptidase La [Rheinheimera sp.] | 89012 | Rheinheimera sp. [Bacteria] | 2 |
| 25 | KOO59404.1 | transcription termination factor Rho [Rheinheimera sp. KL1] | 47413 | [] | 15 |
| 25 | WP_019676918.1 | transcription termination factor Rho [Rheinheimera perlucida] | 47272 | Rheinheimera perlucida [Bacteria] | 3 |
| 26 | WP_134052672.1 | malate dehydrogenase [Rheinheimera aquimaris] | 32462 | Rheinheimera aquimaris [Bacteria] | 10 |
| 26 | TXH95859.1 | malate dehydrogenase [Rheinheimera sp.] | 32549 | Rheinheimera sp. [Bacteria] | 7 |
| 26 | WP_008220944.1 | malate dehydrogenase [Rheinheimera nanhaiensis] | 32515 | Rheinheimera nanhaiensis [Bacteria] | 2 |
| 27 | WP_026349381.1 | DEAD/DEAH box helicase [Rheinheimera perlucida] | 66633 | Rheinheimera perlucida [Bacteria] | 15 |
| 27 | WP_197412273.1 | DEAD/DEAH box helicase [Rheinheimera sp. EpRS3] | 65941 | [] | 1 |
| 27 | WP_127698537.1 | DEAD/DEAH box helicase [Rheinheimera riviphila] | 67137 | Rheinheimera sp. KYPC3 [Bacteria] | 1 |
| 28 | WP_068228927.1 | polyribonucleotide nucleotidyltransferase [Rheinheimera sp. EpRS3] | 76353 | Rheinheimera sp. EpRS3 [Bacteria] | 14 |
| 28 | ALZ76167.1 | polyribonucleotide nucleotidyltransferase [Rheinheimera sp. F8] | 75830 | Rheinheimera sp. F8 [Bacteria] | 2 |
| 28 | WP_092794581.1 | polyribonucleotide nucleotidyltransferase [Rheinheimera pacifica] | 76279 | Rheinheimera pacifica;Gammaproteobacteria bacterium HGW-Gammaproteobacteria-15 [Bacteria] | 1 |
| 29 | WP_068231960.1 | NAD-glutamate dehydrogenase [Rheinheimera sp. EpRS3] | 182258 | Rheinheimera sp. EpRS3 [Bacteria] | 14 |
| 29 | WP_019674347.1 | NAD-glutamate dehydrogenase [Rheinheimera perlucida] | 182220 | Rheinheimera perlucida [Bacteria] | 5 |

| | | | | | |
|---|---|---|---|---|---|
| 29 | WP_068063215.1 | NAD-glutamate dehydrogenase [Rheinheimera sp. SA_1] | 182093 | Rheinheimera sp. SA_1 [Bacteria] | 2 |
| 29 | WP_097109673.1 | NAD-glutamate dehydrogenase [Rheinheimera tuosuensis] | 182021 | Rheinheimera tuosuensis [Bacteria] | 2 |
| 29 | WP_008221649.1 | NAD-glutamate dehydrogenase [Rheinheimera nanhaiensis] | 182353 | Rheinheimera nanhaiensis [Bacteria] | 1 |
| 29 | WP_027670114.1 | NAD-glutamate dehydrogenase [Rheinheimera baltica] | 182204 | Rheinheimera baltica [Bacteria] | 1 |
| 29 | WP_008897386.1 | NAD-glutamate dehydrogenase [Rheinheimera sp. A13L] | 181613 | Rheinheimera sp. A13L [Bacteria] | 1 |
| 30 | WP_019677788.1 | MULTISPECIES: 30S ribosomal protein S7 [Gammaproteobacteria] | 17719 | Rheinheimera perlucida;Rheinheimera tuosuensis [Bacteria] | 13 |
| 31 | WP_027672147.1 | MULTISPECIES: 50S ribosomal protein L2 [Rheinheimera] | 30244 | Rheinheimera baltica [Bacteria] | 13 |
| 31 | WP_008224491.1 | 50S ribosomal protein L2 [Rheinheimera nanhaiensis] | 30200 | Rheinheimera nanhaiensis [Bacteria] | 1 |
| 32 | WP_026349213.1 | MULTISPECIES: ribose-phosphate pyrophosphokinase [Chromatiaceae] | 34381 | Rheinheimera perlucida;Arsukibacterium ikkense [Bacteria] | 13 |
| 33 | WP_173502384.1 | bifunctional proline dehydrogenase/L-glutamate gamma-semialdehyde dehydrogenase PutA [Rheinheimera sp. YQF-2] | 140449 | [] | 13 |
| 33 | WP_097111720.1 | bifunctional proline dehydrogenase/L-glutamate gamma-semialdehyde dehydrogenase PutA [Rheinheimera tuosuensis] | 141100 | Rheinheimera tuosuensis [Bacteria] | 1 |
| 33 | WP_134059159.1 | bifunctional proline dehydrogenase/L-glutamate gamma-semialdehyde dehydrogenase PutA [Rheinheimera aquimaris] | 138528 | Rheinheimera aquimaris [Bacteria] | 1 |
| 34 | WP_173500942.1 | superoxide dismutase [Fe] [Rheinheimera sp. YQF-2] | 21441 | [] | 13 |
| 35 | WP_166839363.1 | 30S ribosomal protein S4 [Rheinheimera pleomorphica] | 23341 | [] | 12 |
| 35 | WP_068238290.1 | MULTISPECIES: 30S ribosomal protein S4 [Rheinheimera] | 23425 | Rheinheimera pacifica;Gammaproteobacteria | 1 |

| | | | | | |
|---|---|---|---|---|---|
| | | | | bacterium HGW-Gammaproteobacteria-15;Rheinheimera sp. EpRS3 [Bacteria] | |
| 36 | WP_134054466.1 | succinate dehydrogenase flavoprotein subunit [Rheinheimera aquimaris] | 65019 | Rheinheimera aquimaris [Bacteria] | 11 |
| 36 | WP_068063429.1 | succinate dehydrogenase flavoprotein subunit [Rheinheimera sp. SA_1] | 64791 | Rheinheimera sp. SA_1 [Bacteria] | 1 |
| 37 | WP_019676211.1 | MULTISPECIES: F0F1 ATP synthase subunit epsilon [Rheinheimera] | 14799 | Rheinheimera perlucida;Rheinheimera tuosuensis [Bacteria] | 11 |
| 38 | WP_019677614.1 | phosphopyruvate hydratase [Rheinheimera perlucida] | 45969 | Rheinheimera perlucida [Bacteria] | 11 |
| 38 | TXH97413.1 | phosphopyruvate hydratase [Rheinheimera sp.] | 46055 | Rheinheimera sp. [Bacteria] | 2 |
| 38 | WP_070048699.1 | phosphopyruvate hydratase [Rheinheimera salexigens] | 45951 | Rheinheimera salexigens [Bacteria] | 1 |
| 39 | WP_008898286.1 | MULTISPECIES: cold-shock protein [Chromatiaceae] | 7741 | Rheinheimera sp. A13L;Pararheinheimera mesophila;Rheinheimera sp. KL1;Pararheinheimera tangshanensis;Rheinheimera sp. YQF-1;Rheinheimera sp. LHK132 [Bacteria] | 11 |
| 40 | WP_166837929.1 | trigger factor [Rheinheimera pleomorphica] | 48238 | [] | 11 |
| 40 | WP_070048840.1 | trigger factor [Rheinheimera salexigens] | 48549 | Rheinheimera salexigens [Bacteria] | 2 |
| 41 | WP_053426066.1 | MULTISPECIES: 50S ribosomal protein L14 [Chromatiaceae] | 13460 | Pararheinheimera tangshanensis;Pararheinheimera mesophila;Rheinheimera sp. KL1 [Bacteria] | 10 |
| 42 | ALZ78045.1 | IMP dehydrogenase [Rheinheimera sp. F8] | 52312 | Rheinheimera sp. F8 [Bacteria] | 10 |
| 42 | TXH96173.1 | IMP dehydrogenase [Rheinheimera sp.] | 52298 | Rheinheimera sp. [Bacteria] | 2 |
| 42 | WP_019675657.1 | IMP dehydrogenase [Rheinheimera perlucida] | 52368 | Rheinheimera perlucida [Bacteria] | 1 |
| 42 | WP_127684819.1 | IMP dehydrogenase [Rheinheimera sp. YQF-1] | 52041 | Rheinheimera sp. YQF-1 [Bacteria] | 1 |

| | | | | | |
|---|---|---|---|---|---|
| 43 | WP_019677731.1 | 50S ribosomal protein L6 [Rheinheimera perlucida] | 18932 | Rheinheimera perlucida [Bacteria] | 10 |
| 44 | WP_019677733.1 | MULTISPECIES: 30S ribosomal protein S5 [Rheinheimera] | 17650 | Rheinheimera perlucida;Rheinheimera tuosuensis [Bacteria] | 10 |
| 44 | WP_068238299.1 | MULTISPECIES: 30S ribosomal protein S5 [Rheinheimera] | 17657 | Rheinheimera pacifica;Gammaproteobacteria bacterium HGW-Gammaproteobacteria-15;Rheinheimera sp. EpRS3 [Bacteria] | 2 |
| 44 | WP_008899103.1 | MULTISPECIES: 30S ribosomal protein S5 [Chromatiaceae] | 17581 | Pararheinheimera tangshanensis;Rheinheimera sp. A13L;Rheinheimera sp. YQF-1;Pararheinheimera mesophila;Rheinheimera sp. KL1 [Bacteria] | 1 |
| 45 | WP_092792540.1 | pyruvate dehydrogenase complex dihydrolipoyllysine-residue acetyltransferase [Rheinheimera pacifica] | 58641 | Rheinheimera pacifica [Bacteria] | 10 |
| 45 | WP_166836787.1 | pyruvate dehydrogenase complex dihydrolipoyllysine-residue acetyltransferase [Rheinheimera pleomorphica] | 57554 | [] | 2 |
| 46 | WP_019677715.1 | MULTISPECIES: 30S ribosomal protein S10 [Chromatiaceae] | 11777 | Rheinheimera tuosuensis;Rheinheimera perlucida;Arsukibacterium sp. MJ3;Arsukibacterium ikkense [Bacteria] | 10 |
| 47 | WP_008899794.1 | MULTISPECIES: 50S ribosomal protein L1 [unclassified Rheinheimera] | 24622 | Rheinheimera sp. A13L;Rheinheimera sp. YQF-1 [Bacteria] | 10 |
| 47 | WP_027672428.1 | 50S ribosomal protein L1 [Rheinheimera baltica] | 24521 | Rheinheimera baltica [Bacteria] | 1 |
| 48 | WP_166839863.1 | NADP-dependent isocitrate dehydrogenase [Rheinheimera pleomorphica] | 80574 | [] | 10 |
| 49 | WP_008220336.1 | MULTISPECIES: DNA-binding protein HU-beta [Rheinheimera] | 9345 | Rheinheimera nanhaiensis;Rheinheimera sp. | 9 |

| | | | | | |
|---|---|---|---|---|---|
| | | | | EpRS3;Rheinheimera aquimaris;Rheinheimera pacifica;Gammaproteobacteria bacterium HGW-Gammaproteobacteria-15;Rheinheimera baltica [Bacteria] | |
| 50 | WP_068228413.1 | glutamine-hydrolyzing GMP synthase [Rheinheimera sp. EpRS3] | 58848 | Rheinheimera sp. EpRS3 [Bacteria] | 9 |
| 51 | WP_027669346.1 | phosphoenolpyruvate carboxykinase (ATP) [Rheinheimera baltica] | 59072 | Rheinheimera baltica [Bacteria] | 9 |
| 51 | WP_132586114.1 | phosphoenolpyruvate carboxykinase (ATP) [Rheinheimera sp. D18] | 59338 | Rheinheimera sp. D18 [Bacteria] | 1 |
| 51 | WP_173502434.1 | phosphoenolpyruvate carboxykinase (ATP) [Rheinheimera sp. YQF-2] | 58706 | [] | 1 |
| 52 | WP_019677720.1 | MULTISPECIES: 30S ribosomal protein S19 [Gammaproteobacteria] | 10435 | Rheinheimera perlucida;Arsukibacterium sp. MJ3;Rheinheimera tuosuensis;Alkalimonas amylolytica;Arsukibacterium ikkense;Rheinheimera sp. D18 [Bacteria] | 9 |
| 53 | WP_092795534.1 | ATP-dependent chaperone ClpB [Rheinheimera pacifica] | 96284 | Rheinheimera pacifica [Bacteria] | 9 |
| 54 | WP_027670184.1 | acyl carrier protein [Rheinheimera baltica] | 8749 | Rheinheimera baltica [Bacteria] | 9 |
| 55 | WP_068230209.1 | S46 family peptidase [Rheinheimera sp. EpRS3] | 79276 | Rheinheimera sp. EpRS3 [Bacteria] | 9 |
| 55 | WP_092791717.1 | S46 family peptidase [Rheinheimera pacifica] | 79095 | Rheinheimera pacifica [Bacteria] | 1 |
| 55 | WP_008219464.1 | S46 family peptidase [Rheinheimera nanhaiensis] | 78848 | Rheinheimera nanhaiensis [Bacteria] | 1 |
| 56 | WP_173499325.1 | protein-export chaperone SecB [Rheinheimera sp. YQF-2] | 18235 | [] | 9 |
| 57 | WP_008218806.1 | MULTISPECIES: nucleoside-diphosphate kinase [Rheinheimera] | 15618 | Rheinheimera nanhaiensis [Bacteria] | 9 |
| 57 | WP_019675675.1 | MULTISPECIES: nucleoside-diphosphate kinase [Chromatiaceae] | 15760 | Rheinheimera perlucida;Arsukibacterium ikkense [Bacteria] | 3 |

| | | | | | |
|---|---|---|---|---|---|
| 58 | WP_070048546.1 | translational GTPase TypA [Rheinheimera salexigens] | 67579 | Rheinheimera salexigens [Bacteria] | 9 |
| 58 | WP_027670281.1 | translational GTPase TypA [Rheinheimera baltica] | 67439 | Rheinheimera baltica [Bacteria] | 3 |
| 59 | WP_027669831.1 | 30S ribosomal protein S2 [Rheinheimera baltica] | 27044 | Rheinheimera baltica [Bacteria] | 9 |
| 59 | WP_008223614.1 | 30S ribosomal protein S2 [Rheinheimera nanhaiensis] | 26897 | Rheinheimera nanhaiensis [Bacteria] | 2 |
| 59 | WP_019674885.1 | MULTISPECIES: 30S ribosomal protein S2 [Chromatiaceae] | 26974 | Rheinheimera perlucida;Arsukibacterium ikkense [Bacteria] | 1 |
| 60 | WP_068237158.1 | glycine--tRNA ligase subunit beta [Rheinheimera sp. EpRS3] | 74365 | Rheinheimera sp. EpRS3 [Bacteria] | 8 |
| 60 | WP_092790630.1 | glycine--tRNA ligase subunit beta [Rheinheimera pacifica] | 74480 | Rheinheimera pacifica [Bacteria] | 2 |
| 61 | WP_132584808.1 | UMP kinase [Rheinheimera sp. D18] | 26610 | Rheinheimera sp. D18 [Bacteria] | 8 |
| 62 | WP_134054470.1 | 2-oxoglutarate dehydrogenase E1 component [Rheinheimera aquimaris] | 105321 | Rheinheimera aquimaris [Bacteria] | 7 |
| 62 | WP_097109850.1 | 2-oxoglutarate dehydrogenase E1 component [Rheinheimera tuosuensis] | 105294 | Rheinheimera tuosuensis [Bacteria] | 4 |
| 62 | WP_008223099.1 | 2-oxoglutarate dehydrogenase E1 component [Rheinheimera nanhaiensis] | 105165 | Rheinheimera nanhaiensis [Bacteria] | 2 |
| 63 | WP_127697482.1 | succinate dehydrogenase iron-sulfur subunit [Rheinheimera riviphila] | 27245 | Rheinheimera sp. KYPC3 [Bacteria] | 8 |
| 63 | WP_046518779.1 | MULTISPECIES: succinate dehydrogenase iron-sulfur subunit [Chromatiaceae] | 27331 | Pararheinheimera tangshanensis;Pararheinheimera mesophila;Rheinheimera sp. KL1 [Bacteria] | 1 |
| 64 | WP_019675905.1 | acetyl-CoA carboxylase biotin carboxylase subunit [Rheinheimera perlucida] | 49428 | Rheinheimera perlucida [Bacteria] | 8 |
| 64 | WP_092789649.1 | acetyl-CoA carboxylase biotin carboxylase subunit [Rheinheimera pacifica] | 49234 | Rheinheimera pacifica;Gammaproteobacteria bacterium HGW-Gammaproteobacteria-15 [Bacteria] | 2 |
| 64 | WP_173502791.1 | acetyl-CoA carboxylase biotin carboxylase subunit [Rheinheimera sp. YQF-2] | 49309 | [] | 1 |

| | | | | | |
|---|---|---|---|---|---|
| 65 | WP_068059796.1 | 50S ribosomal protein L7/L12 [Rheinheimera sp. SA_1] | 12274 | Rheinheimera sp. SA_1 [Bacteria] | 8 |
| 65 | WP_173502801.1 | 50S ribosomal protein L7/L12 [Rheinheimera sp. YQF-2] | 12143 | [] | 3 |
| 65 | WP_127688356.1 | 50S ribosomal protein L7/L12 [Rheinheimera sp. YQF-1] | 12228 | Rheinheimera sp. YQF-1 [Bacteria] | 1 |
| 66 | WP_132585989.1 | 30S ribosomal protein S3 [Rheinheimera sp. D18] | 26285 | Rheinheimera sp. D18 [Bacteria] | 8 |
| 66 | WP_127019507.1 | 30S ribosomal protein S3 [Rheinheimera sp. LHK132] | 26288 | Rheinheimera sp. LHK132 [Bacteria] | 1 |
| 67 | WP_134053714.1 | flagellin FliC [Rheinheimera aquimaris] | 29264 | Rheinheimera aquimaris [Bacteria] | 8 |
| 67 | WP_008220157.1 | flagellin [Rheinheimera nanhaiensis] | 28971 | Rheinheimera nanhaiensis [Bacteria] | 1 |
| 67 | WP_132584432.1 | flagellin FliC [Rheinheimera sp. D18] | 29703 | Rheinheimera sp. D18 [Bacteria] | 1 |
| 68 | WP_019675368.1 | 50S ribosomal protein L19 [Rheinheimera perlucida] | 13395 | Rheinheimera perlucida [Bacteria] | 8 |
| 69 | WP_019677727.1 | 50S ribosomal protein L24 [Rheinheimera perlucida] | 11524 | Rheinheimera perlucida [Bacteria] | 8 |
| 70 | WP_070048953.1 | aminomethyl-transferring glycine dehydrogenase [Rheinheimera salexigens] | 104873 | Rheinheimera salexigens [Bacteria] | 8 |
| 70 | WP_166840759.1 | aminomethyl-transferring glycine dehydrogenase [Rheinheimera pleomorphica] | 104879 | [] | 4 |
| 70 | WP_134053980.1 | aminomethyl-transferring glycine dehydrogenase [Rheinheimera aquimaris] | 104872 | Rheinheimera aquimaris [Bacteria] | 1 |
| 71 | WP_092790354.1 | paraslipin [Rheinheimera pacifica] | 34349 | Rheinheimera pacifica [Bacteria] | 8 |
| 72 | WP_008898288.1 | serine hydroxymethyltransferase [Rheinheimera sp. A13L] | 45269 | Rheinheimera sp. A13L [Bacteria] | 8 |
| 73 | WP_027672427.1 | MULTISPECIES: 50S ribosomal protein L11 [Rheinheimera] | 14960 | Rheinheimera baltica;Rheinheimera aquimaris [Bacteria] | 8 |
| 74 | WP_173500211.1 | FKBP-type peptidyl-prolyl cis-trans isomerase [Rheinheimera sp. YQF-2] | 21851 | [] | 7 |
| 74 | WP_027671609.1 | FKBP-type peptidyl-prolyl cis-trans isomerase [Rheinheimera baltica] | 21854 | Rheinheimera baltica [Bacteria] | 2 |
| 75 | WP_026349244.1 | preprotein translocase subunit SecA [Rheinheimera perlucida] | 102409 | Rheinheimera perlucida [Bacteria] | 7 |

| | | | | | |
|---|---|---|---|---|---|
| 75 | WP_068062096.1 | preprotein translocase subunit SecA [Rheinheimera sp. SA_1] | 101936 | Rheinheimera sp. SA_1 [Bacteria] | 2 |
| 75 | WP_070048472.1 | preprotein translocase subunit SecA [Rheinheimera salexigens] | 102491 | Rheinheimera salexigens [Bacteria] | 1 |
| 76 | WP_008218371.1 | biopolymer transporter ExbD [Rheinheimera nanhaiensis] | 14892 | Rheinheimera nanhaiensis [Bacteria] | 7 |
| 76 | WP_166839892.1 | biopolymer transporter ExbD [Rheinheimera pleomorphica] | 14847 | [] | 3 |
| 77 | TXH94010.1 | outer membrane channel protein TolC [Rheinheimera sp.] | 46750 | Rheinheimera sp. [Bacteria] | 7 |
| 77 | WP_127025788.1 | outer membrane channel protein TolC [Rheinheimera sp. LHK132] | 48481 | Rheinheimera sp. LHK132 [Bacteria] | 2 |
| 78 | WP_027670474.1 | CTP synthase [Rheinheimera baltica] | 60182 | Rheinheimera baltica [Bacteria] | 7 |
| 79 | WP_008224462.1 | MULTISPECIES: 30S ribosomal protein S13 [Rheinheimera] | 13248 | Rheinheimera nanhaiensis [Bacteria] | 7 |
| 80 | WP_092789703.1 | adenylosuccinate synthase [Rheinheimera pacifica] | 46887 | Rheinheimera pacifica [Bacteria] | 7 |
| 81 | WP_097113082.1 | 50S ribosomal protein L22 [Rheinheimera tuosuensis] | 12070 | Rheinheimera tuosuensis [Bacteria] | 7 |
| 82 | WP_019677717.1 | MULTISPECIES: 50S ribosomal protein L4 [Chromatiaceae] | 22155 | Rheinheimera perlucida;Arsukibacterium ikkense [Bacteria] | 7 |
| 83 | WP_134057555.1 | 50S ribosomal protein L9 [Rheinheimera aquimaris] | 15646 | Rheinheimera aquimaris [Bacteria] | 7 |
| 83 | WP_031568570.1 | 50S ribosomal protein L9 [Pararheinheimera texasensis] | 15630 | Rheinheimera sp.;Pararheinheimera texasensis [Bacteria] | 2 |
| 83 | WP_097111462.1 | 50S ribosomal protein L9 [Rheinheimera tuosuensis] | 15806 | Rheinheimera tuosuensis [Bacteria] | 1 |
| 83 | WP_008899547.1 | 50S ribosomal protein L9 [Rheinheimera sp. A13L] | 15709 | Rheinheimera sp. A13L [Bacteria] | 1 |
| 84 | WP_068232999.1 | glutamate--ammonia ligase [Rheinheimera sp. EpRS3] | 51938 | Rheinheimera sp. EpRS3 [Bacteria] | 7 |
| 84 | WP_166837124.1 | glutamate--ammonia ligase [Rheinheimera pleomorphica] | 51993 | [] | 1 |
| 85 | WP_027669736.1 | translation initiation factor IF-2 [Rheinheimera baltica] | 97851 | Rheinheimera baltica [Bacteria] | 7 |

| | | | | | |
|---|---|---|---|---|---|
| 85 | WP_008900380.1 | translation initiation factor IF-2 [Rheinheimera sp. A13L] | 97993 | Rheinheimera sp. A13L [Bacteria] | 4 |
| 85 | WP_070050210.1 | translation initiation factor IF-2 [Rheinheimera salexigens] | 98006 | Rheinheimera salexigens [Bacteria] | 2 |
| 85 | WP_008223871.1 | translation initiation factor IF-2 [Rheinheimera nanhaiensis] | 97865 | Rheinheimera nanhaiensis [Bacteria] | 1 |
| 85 | WP_127699051.1 | translation initiation factor IF-2 [Rheinheimera riviphila] | 97687 | Rheinheimera sp. KYPC3 [Bacteria] | 1 |
| 86 | WP_166839873.1 | adenylosuccinate lyase [Rheinheimera pleomorphica] | 51158 | [] | 7 |
| 87 | WP_097110285.1 | aspartate/tyrosine/aromatic aminotransferase [Rheinheimera tuosuensis] | 44064 | Rheinheimera tuosuensis [Bacteria] | 6 |
| 88 | WP_008219881.1 | MULTISPECIES: rod shape-determining protein [Rheinheimera] | 37088 | Rheinheimera nanhaiensis;Rheinheimera sp. EpRS3;Rheinheimera aquimaris;Rheinheimera pacifica;Gammaproteobacteria bacterium HGW-Gammaproteobacteria-15;Rheinheimera baltica [Bacteria] | 6 |
| 89 | WP_068232166.1 | MULTISPECIES: two-component system response regulator ArcA [Rheinheimera] | 26922 | Rheinheimera pacifica;Gammaproteobacteria bacterium HGW-Gammaproteobacteria-15;Rheinheimera sp. EpRS3 [Bacteria] | 6 |
| 90 | WP_040555383.1 | ATP-dependent zinc metalloprotease FtsH [Rheinheimera sp. A13L] | 69919 | [] | 6 |
| 90 | WP_177172250.1 | ATP-dependent zinc metalloprotease FtsH [Rheinheimera pacifica] | 70079 | [] | 3 |
| 90 | WP_068062834.1 | ATP-dependent zinc metalloprotease FtsH [Rheinheimera sp. SA_1] | 70048 | [] | 2 |
| 91 | WP_019674403.1 | glyceraldehyde-3-phosphate dehydrogenase [Rheinheimera perlucida] | 53746 | Rheinheimera perlucida [Bacteria] | 6 |
| 92 | WP_068064397.1 | ribosome recycling factor [Rheinheimera sp. SA_1] | 20716 | Rheinheimera sp. SA_1 [Bacteria] | 6 |

| | | | | | |
|---|---|---|---|---|---|
| 92 | WP_097112024.1 | ribosome recycling factor [Rheinheimera tuosuensis] | 20591 | Rheinheimera tuosuensis [Bacteria] | 2 |
| 92 | WP_053425487.1 | ribosome recycling factor [Rheinheimera tangshanensis] | 20663 | Pararheinheimera tangshanensis;Rheinheimera sp. KL1 [Bacteria] | 2 |
| 93 | WP_097112565.1 | M3 family metallopeptidase [Rheinheimera tuosuensis] | 80912 | Rheinheimera tuosuensis [Bacteria] | 6 |
| 93 | WP_068227550.1 | M3 family metallopeptidase [Rheinheimera sp. EpRS3] | 81257 | Rheinheimera sp. EpRS3 [Bacteria] | 2 |
| 93 | WP_019677318.1 | M3 family metallopeptidase [Rheinheimera perlucida] | 80469 | Rheinheimera perlucida [Bacteria] | 1 |
| 94 | WP_019675735.1 | HslU--HslV peptidase ATPase subunit [Rheinheimera perlucida] | 49921 | Rheinheimera perlucida [Bacteria] | 6 |
| 94 | WP_134051903.1 | HslU--HslV peptidase ATPase subunit [Rheinheimera aquimaris] | 49588 | Rheinheimera aquimaris [Bacteria] | 2 |
| 95 | WP_134053430.1 | F0F1 ATP synthase subunit B [Rheinheimera aquimaris] | 17368 | Rheinheimera aquimaris [Bacteria] | 6 |
| 96 | WP_097109613.1 | DNA topoisomerase (ATP-hydrolyzing) subunit A [Rheinheimera tuosuensis] | 98962 | Rheinheimera tuosuensis [Bacteria] | 6 |
| 97 | WP_026349552.1 | elongation factor G [Rheinheimera perlucida] | 77837 | Rheinheimera perlucida [Bacteria] | 6 |
| 98 | WP_127700002.1 | phosphate ABC transporter substrate-binding protein PstS family protein [Rheinheimera riviphila] | 35055 | Rheinheimera sp. KYPC3 [Bacteria] | 6 |
| 99 | WP_019676213.1 | MULTISPECIES: F0F1 ATP synthase subunit gamma [Rheinheimera] | 31824 | Rheinheimera perlucida;Rheinheimera tuosuensis [Bacteria] | 6 |
| 99 | ALZ74695.1 | ATP F0F1 synthase subunit gamma [Rheinheimera sp. F8] | 31672 | Rheinheimera sp. F8 [Bacteria] | 1 |
| 100 | WP_173502166.1 | homogentisate 1,2-dioxygenase [Rheinheimera sp. YQF-2] | 43945 | [] | 6 |
| 100 | WP_070048858.1 | homogentisate 1,2-dioxygenase [Rheinheimera salexigens] | 43176 | Rheinheimera salexigens [Bacteria] | 1 |
| 101 | WP_027672163.1 | MULTISPECIES: 30S ribosomal protein S11 [Rheinheimera] | 13859 | Rheinheimera baltica;Rheinheimera aquimaris [Bacteria] | 6 |

| | | | | | |
|---|---|---|---|---|---|
| 102 | WP_008223024.1 | 2-methylcitrate synthase [Rheinheimera nanhaiensis] | 41750 | Rheinheimera nanhaiensis [Bacteria] | 6 |
| 102 | WP_097109815.1 | 2-methylcitrate synthase [Rheinheimera tuosuensis] | 42024 | Rheinheimera tuosuensis [Bacteria] | 1 |
| 103 | WP_166839379.1 | Vi polysaccharide biosynthesis UDP-N-acetylglucosamine C-6 dehydrogenase TviB, partial [Rheinheimera pleomorphica] | 41279 | [] | 6 |
| 104 | WP_097113079.1 | 30S ribosomal protein S8 [Rheinheimera tuosuensis] | 13924 | Rheinheimera tuosuensis [Bacteria] | 6 |
| 105 | WP_092793231.1 | Na(+)-translocating NADH-quinone reductase subunit A [Rheinheimera pacifica] | 47536 | Rheinheimera pacifica [Bacteria] | 6 |
| 106 | WP_092790116.1 | TonB-dependent siderophore receptor [Rheinheimera pacifica] | 75044 | Rheinheimera pacifica [Bacteria] | 6 |
| 107 | WP_040552631.1 | 50S ribosomal protein L18 [Rheinheimera nanhaiensis] | 12690 | Rheinheimera nanhaiensis [Bacteria] | 6 |
| 108 | WP_070049453.1 | methionine adenosyltransferase [Rheinheimera salexigens] | 42051 | Rheinheimera salexigens [Bacteria] | 5 |
| 109 | ALZ75678.1 | glutamine--fructose-6-phosphate aminotransferase [Rheinheimera sp. F8] | 66225 | Rheinheimera sp. F8 [Bacteria] | 5 |
| 109 | WP_068237017.1 | glutamine--fructose-6-phosphate transaminase (isomerizing) [Rheinheimera sp. EpRS3] | 65759 | Rheinheimera sp. EpRS3 [Bacteria] | 1 |
| 110 | WP_019677718.1 | MULTISPECIES: 50S ribosomal protein L23 [Chromatiaceae] | 10987 | Rheinheimera perlucida;Arsukibacterium sp. MJ3;Rheinheimera tuosuensis [Bacteria] | 5 |
| 111 | WP_019677364.1 | isovaleryl-CoA dehydrogenase [Rheinheimera perlucida] | 42765 | Rheinheimera perlucida [Bacteria] | 5 |
| 112 | WP_068234274.1 | MULTISPECIES: 30S ribosomal protein S16 [Rheinheimera] | 9267 | Rheinheimera pacifica;Gammaproteobacteria bacterium HGW-Gammaproteobacteria-15;Rheinheimera sp. EpRS3 [Bacteria] | 5 |
| 113 | WP_019675952.1 | inorganic diphosphatase [Rheinheimera perlucida] | 19564 | Rheinheimera perlucida [Bacteria] | 5 |

| | | | | | |
|---|---|---|---|---|---|
| 114 | WP_127688195.1 | transcription termination/antitermination protein NusA [Rheinheimera sp. YQF-1] | 55317 | Rheinheimera sp. YQF-1 [Bacteria] | 5 |
| 114 | WP_070050211.1 | transcription termination/antitermination protein NusA [Rheinheimera salexigens] | 55511 | Rheinheimera salexigens [Bacteria] | 1 |
| 115 | WP_127700708.1 | FtsH protease activity modulator HflK [Rheinheimera riviphila] | 42454 | Rheinheimera sp. KYPC3 [Bacteria] | 2 |
| 116 | WP_134057576.1 | FtsH protease activity modulator HflK [Rheinheimera aquimaris] | 42550 | Rheinheimera aquimaris [Bacteria] | 5 |
| 117 | WP_068233191.1 | M13 family peptidase [Rheinheimera sp. EpRS3] | 76738 | Rheinheimera sp. EpRS3 [Bacteria] | 5 |
| 118 | WP_027670345.1 | NADH:ubiquinone reductase (Na(+)-transporting) subunit F [Rheinheimera baltica] | 46050 | Rheinheimera baltica [Bacteria] | 5 |
| 119 | WP_019677083.1 | phosphoribosylformylglycinamidine synthase [Rheinheimera perlucida] | 141210 | Rheinheimera perlucida [Bacteria] | 5 |
| 119 | TXH96169.1 | phosphoribosylformylglycinamidine synthase [Rheinheimera sp.] | 141505 | Rheinheimera sp. [Bacteria] | 4 |
| 119 | WP_173502552.1 | phosphoribosylformylglycinamidine synthase [Rheinheimera sp. YQF-2] | 140904 | [] | 1 |
| 120 | WP_027669828.1 | 2,3,4,5-tetrahydropyridine-2,6-dicarboxylate N-succinyltransferase [Rheinheimera baltica] | 29506 | Rheinheimera baltica [Bacteria] | 5 |
| 121 | WP_097109631.1 | DUF533 domain-containing protein [Rheinheimera tuosuensis] | 25194 | Rheinheimera tuosuensis [Bacteria] | 5 |
| 122 | WP_092793093.1 | elongation factor Ts [Rheinheimera pacifica] | 30509 | Rheinheimera pacifica [Bacteria] | 5 |
| 122 | WP_097112023.1 | elongation factor Ts [Rheinheimera tuosuensis] | 30774 | Rheinheimera tuosuensis [Bacteria] | 2 |
| 123 | WP_070049446.1 | MotA/TolQ/ExbB proton channel family protein [Rheinheimera salexigens] | 49579 | Rheinheimera salexigens [Bacteria] | 5 |
| 124 | WP_134053830.1 | chemotaxis protein CheW [Rheinheimera aquimaris] | 18156 | Rheinheimera aquimaris [Bacteria] | 5 |
| 124 | WP_019676457.1 | MULTISPECIES: chemotaxis protein CheW [Rheinheimera] | 18101 | Rheinheimera perlucida;Rheinheimera tuosuensis [Bacteria] | 1 |
| 125 | WP_173500107.1 | Fe/S-dependent 2-methylisocitrate dehydratase AcnD [Rheinheimera sp. YQF-2] | 94754 | [] | 5 |
| 126 | WP_134059710.1 | 50S ribosomal protein L15 [Rheinheimera aquimaris] | 14887 | Rheinheimera aquimaris [Bacteria] | 5 |

| | | | | | |
|---|---|---|---|---|---|
| 126 | WP_019677734.1 | MULTISPECIES: 50S ribosomal protein L15 [Rheinheimera] | 14946 | Rheinheimera perlucida;Rheinheimera tuosuensis [Bacteria] | 2 |
| 127 | WP_019676516.1 | amidohydrolase [Rheinheimera perlucida] | 47134 | Rheinheimera perlucida [Bacteria] | 5 |
| 128 | WP_027669935.1 | 30S ribosomal protein S9 [Rheinheimera baltica] | 14700 | Rheinheimera baltica [Bacteria] | 5 |
| 128 | WP_070048178.1 | 30S ribosomal protein S9 [Rheinheimera salexigens] | 14714 | Rheinheimera salexigens [Bacteria] | 1 |
| 129 | WP_008897591.1 | 4-hydroxy-tetrahydrodipicolinate synthase [Rheinheimera sp. A13L] | 34427 | Rheinheimera sp. A13L [Bacteria] | 5 |
| 129 | WP_166837796.1 | 4-hydroxy-tetrahydrodipicolinate synthase [Rheinheimera pleomorphica] | 34075 | [] | 1 |
| 130 | WP_132585516.1 | 50S ribosomal protein L28 [Rheinheimera sp. D18] | 8941 | Rheinheimera sp. D18 [Bacteria] | 5 |
| 131 | WP_092794608.1 | phosphoglucosamine mutase [Rheinheimera pacifica] | 48314 | Rheinheimera pacifica [Bacteria] | 5 |
| 132 | WP_134052371.1 | glycine C-acetyltransferase [Rheinheimera aquimaris] | 43289 | Rheinheimera aquimaris [Bacteria] | 5 |
| 132 | WP_070048317.1 | glycine C-acetyltransferase [Rheinheimera salexigens] | 43390 | Rheinheimera salexigens [Bacteria] | 1 |
| 133 | WP_092791416.1 | CoA transferase subunit A [Rheinheimera pacifica] | 25367 | Rheinheimera pacifica [Bacteria] | 5 |
| 134 | WP_068228941.1 | serine hydrolase [Rheinheimera sp. EpRS3] | 42562 | Rheinheimera sp. EpRS3 [Bacteria] | 5 |
| 135 | WP_019676917.1 | thioredoxin TrxA [Rheinheimera perlucida] | 11950 | Rheinheimera perlucida [Bacteria] | 5 |
| 136 | WP_019675926.1 | RNA chaperone Hfq [Rheinheimera perlucida] | 9613 | Rheinheimera perlucida [Bacteria] | 5 |
| 137 | WP_173502496.1 | 50S ribosomal protein L25/general stress protein Ctc [Rheinheimera sp. YQF-2] | 21975 | [] | 5 |
| 138 | WP_097112099.1 | phage shock protein PspA [Rheinheimera tuosuensis] | 25513 | Rheinheimera tuosuensis [Bacteria] | 4 |
| 139 | WP_132583565.1 | methylisocitrate lyase [Rheinheimera sp. D18] | 31917 | Rheinheimera sp. D18 [Bacteria] | 4 |
| 140 | WP_008218940.1 | MULTISPECIES: 30S ribosomal protein S18 [Gammaproteobacteria] | 8760 | Rheinheimera perlucida;Arsukibacterium sp. MJ3;Rheinheimera nanhaiensis;Alishewanella sp. HH-ZS;Rheinheimera aquimaris;Rheinheimera | 4 |

| | | | | | |
|---|---|---|---|---|---|
| | | | | tuosuensis;Alishewanella aestuarii;Alishewanella sp. WH16-1;Alishewanella jeotgali;Rheinheimera sp. D18;Arsukibacterium ikkense;Rheinheimera baltica;Alishewanella sp. 34-51-39;Alishewanella agri [Bacteria] | |
| 141 | WP_092794667.1 | molecular chaperone DnaJ [Rheinheimera pacifica] | 41219 | Rheinheimera pacifica;Gammaproteobacteria bacterium HGW-Gammaproteobacteria-15 [Bacteria] | 4 |
| 142 | WP_068233486.1 | oligopeptidase A [Rheinheimera sp. EpRS3] | 76537 | Rheinheimera sp. EpRS3 [Bacteria] | 4 |
| 142 | WP_008218629.1 | oligopeptidase A [Rheinheimera nanhaiensis] | 76456 | Rheinheimera nanhaiensis [Bacteria] | 1 |
| 143 | WP_097112523.1 | ribosome biogenesis GTPase Der [Rheinheimera tuosuensis] | 54469 | Rheinheimera tuosuensis [Bacteria] | 4 |
| 144 | WP_173500784.1 | asparagine--tRNA ligase [Rheinheimera sp. YQF-2] | 52645 | [] | 4 |
| 145 | WP_019677783.1 | MULTISPECIES: 50S ribosomal protein L10 [Chromatiaceae] | 17637 | Rheinheimera perlucida;Arsukibacterium ikkense [Bacteria] | 4 |
| 146 | WP_173501637.1 | 50S ribosomal protein L27 [Rheinheimera sp. YQF-2] | 9163 | [] | 4 |
| 147 | WP_173500517.1 | chemotaxis protein CheW [Rheinheimera sp. YQF-2] | 76736 | [] | 4 |
| 147 | WP_147904378.1 | chemotaxis protein CheA [Rheinheimera tangshanensis] | 79363 | Pararheinheimera tangshanensis [Bacteria] | 2 |
| 148 | WP_068234836.1 | acetyl-CoA C-acyltransferase [Rheinheimera sp. EpRS3] | 40451 | Rheinheimera sp. EpRS3 [Bacteria] | 4 |
| 149 | TXH97542.1 | single-stranded DNA-binding protein [Rheinheimera sp.] | 24382 | Rheinheimera sp. [Bacteria] | 4 |
| 150 | WP_097111927.1 | lysine--tRNA ligase [Rheinheimera tuosuensis] | 57881 | Rheinheimera tuosuensis [Bacteria] | 4 |
| 151 | WP_134055938.1 | CoA-acylating methylmalonate-semialdehyde dehydrogenase [Rheinheimera aquimaris] | 54244 | Rheinheimera aquimaris [Bacteria] | 4 |
| 152 | WP_092790720.1 | cysteine synthase A [Rheinheimera pacifica] | 34252 | Rheinheimera pacifica [Bacteria] | 4 |

| | | | | | |
|---|---|---|---|---|---|
| 152 | WP_127687360.1 | cysteine synthase A [Rheinheimera sp. YQF-1] | 34305 | Rheinheimera sp. YQF-1 [Bacteria] | 1 |
| 153 | WP_019675269.1 | MULTISPECIES: 50S ribosomal protein L13 [Rheinheimera] | 15856 | Rheinheimera perlucida;Rheinheimera tuosuensis [Bacteria] | 4 |
| 154 | WP_027670166.1 | MULTISPECIES: cold-shock protein [Gammaproteobacteria] | 7499 | Rheinheimera salexigens;Rheinheimera sp. EpRS3;Rheinheimera pacifica;Gammaproteobacteria bacterium HGW-Gammaproteobacteria-15;Rheinheimera baltica;Rheinheimera sp. D18 [Bacteria] | 4 |
| 155 | ALZ74478.1 | acetyl-CoA carboxylase subunit beta [Rheinheimera sp. F8] | 32339 | Rheinheimera sp. F8 [Bacteria] | 4 |
| 156 | WP_097110595.1 | cytochrome c4 [Rheinheimera tuosuensis] | 22291 | Rheinheimera tuosuensis [Bacteria] | 4 |
| 157 | WP_147903602.1 | methyl-accepting chemotaxis protein [Rheinheimera tangshanensis] | 71496 | Pararheinheimera tangshanensis [Bacteria] | 4 |
| 157 | WP_070048193.1 | methyl-accepting chemotaxis protein [Rheinheimera salexigens] | 71910 | Rheinheimera salexigens [Bacteria] | 1 |
| 157 | WP_164731887.1 | methyl-accepting chemotaxis protein [Rheinheimera sp. LHK132] | 51956 | [] | 1 |
| 158 | TXH96059.1 | IscS subfamily cysteine desulfurase [Rheinheimera sp.] | 44780 | Rheinheimera sp. [Bacteria] | 4 |
| 158 | WP_127697210.1 | IscS subfamily cysteine desulfurase [Rheinheimera riviphila] | 44698 | Rheinheimera sp. KYPC3 [Bacteria] | 2 |
| 159 | WP_134055321.1 | phenylalanine--tRNA ligase subunit alpha [Rheinheimera aquimaris] | 37126 | Rheinheimera aquimaris [Bacteria] | 4 |
| 160 | TXH98634.1 | phosphoenolpyruvate synthase [Rheinheimera sp.] | 86978 | Rheinheimera sp. [Bacteria] | 4 |
| 160 | WP_019674242.1 | phosphoenolpyruvate synthase [Rheinheimera perlucida] | 87284 | Rheinheimera perlucida [Bacteria] | 1 |
| 161 | WP_173501361.1 | DNA starvation/stationary phase protection protein [Rheinheimera sp. YQF-2] | 17288 | [] | 4 |

| | | | | | |
|---|---|---|---|---|---|
| 162 | WP_134057739.1 | preprotein translocase subunit YajC [Rheinheimera aquimaris] | 12144 | Rheinheimera aquimaris [Bacteria] | 4 |
| 163 | WP_166838036.1 | redox-regulated ATPase YchF [Rheinheimera pleomorphica] | 39691 | [] | 4 |
| 164 | WP_068060656.1 | protease modulator HflC [Rheinheimera sp. SA_1] | 32852 | Rheinheimera sp. SA_1 [Bacteria] | 4 |
| 165 | WP_166837579.1 | phage tail sheath subtilisin-like domain-containing protein [Rheinheimera pleomorphica] | 57247 | [] | 4 |
| 165 | WP_068229263.1 | phage tail sheath family protein [Rheinheimera sp. EpRS3] | 57249 | Gammaproteobacteria bacterium HGW-Gammaproteobacteria-15;Rheinheimera sp. EpRS3 [Bacteria] | 1 |
| 166 | WP_134055319.1 | phenylalanine--tRNA ligase subunit beta [Rheinheimera aquimaris] | 86981 | Rheinheimera aquimaris [Bacteria] | 4 |
| 166 | WP_127021878.1 | phenylalanine--tRNA ligase subunit beta [Rheinheimera sp. LHK132] | 86625 | Rheinheimera sp. LHK132 [Bacteria] | 1 |
| 167 | WP_027669669.1 | fumarylacetoacetate hydrolase family protein [Rheinheimera baltica] | 36891 | Rheinheimera baltica [Bacteria] | 4 |
| 167 | ALZ75097.1 | 2-keto-4-pentenoate hydratase [Rheinheimera sp. F8] | 36909 | Rheinheimera sp. F8 [Bacteria] | 1 |
| 168 | WP_166840840.1 | ribonuclease E [Rheinheimera pleomorphica] | 113046 | [] | 4 |
| 168 | WP_019675613.1 | ribonuclease E [Rheinheimera perlucida] | 117131 | Rheinheimera perlucida [Bacteria] | 2 |
| 169 | WP_008218160.1 | acyl-CoA dehydrogenase family protein [Rheinheimera nanhaiensis] | 42493 | Rheinheimera nanhaiensis [Bacteria] | 3 |
| 170 | WP_166838445.1 | biosynthetic arginine decarboxylase [Rheinheimera pleomorphica] | 70598 | [] | 3 |
| 171 | WP_070047941.1 | BON domain-containing protein [Rheinheimera salexigens] | 20396 | Rheinheimera salexigens [Bacteria] | 3 |
| 172 | TXH94460.1 | glycine cleavage system aminomethyltransferase GcvT [Rheinheimera sp.] | 38766 | Rheinheimera sp. [Bacteria] | 3 |
| 173 | WP_092792334.1 | histidine ammonia-lyase [Rheinheimera pacifica] | 54644 | Rheinheimera pacifica [Bacteria] | 3 |
| 174 | WP_166837800.1 | TonB-dependent receptor [Rheinheimera pleomorphica] | 109782 | [] | 3 |
| 175 | WP_027672818.1 | tellurite resistance TerB family protein [Rheinheimera baltica] | 24376 | Rheinheimera baltica [Bacteria] | 3 |

| | | | | | |
|---|---|---|---|---|---|
| 176 | WP_097112466.1 | ATP-dependent protease subunit HslV [Rheinheimera tuosuensis] | 18605 | Rheinheimera tuosuensis [Bacteria] | 3 |
| 177 | WP_134053470.1 | DNA topoisomerase (ATP-hydrolyzing) subunit B [Rheinheimera aquimaris] | 90319 | Rheinheimera aquimaris [Bacteria] | 3 |
| 178 | WP_026349132.1 | ABC-F family ATPase [Rheinheimera perlucida] | 59543 | Rheinheimera perlucida [Bacteria] | 3 |
| 179 | WP_166839610.1 | type I glyceraldehyde-3-phosphate dehydrogenase [Rheinheimera pleomorphica] | 36660 | [] | 3 |
| 179 | WP_132583515.1 | type I glyceraldehyde-3-phosphate dehydrogenase [Rheinheimera sp. D18] | 36952 | Rheinheimera sp. D18 [Bacteria] | 1 |
| 180 | WP_127698667.1 | ATP-dependent RNA helicase RhlB [Rheinheimera riviphila] | 47516 | Rheinheimera sp. KYPC3 [Bacteria] | 3 |
| 180 | WP_127019191.1 | ATP-dependent RNA helicase RhlB [Rheinheimera sp. LHK132] | 47622 | Rheinheimera sp. LHK132 [Bacteria] | 1 |
| 181 | WP_019675525.1 | asparagine synthase B [Rheinheimera perlucida] | 62860 | Rheinheimera perlucida [Bacteria] | 3 |
| 182 | WP_097111410.1 | phosphate signaling complex protein PhoU [Rheinheimera tuosuensis] | 27129 | Rheinheimera tuosuensis [Bacteria] | 3 |
| 183 | WP_173501474.1 | cell division protein FtsZ [Rheinheimera sp. YQF-2] | 40638 | [] | 3 |
| 184 | WP_027672038.1 | beta-ketoacyl-ACP synthase I [Rheinheimera baltica] | 42447 | Rheinheimera baltica [Bacteria] | 3 |
| 185 | WP_019677724.1 | MULTISPECIES: 50S ribosomal protein L29 [Rheinheimera] | 7061 | Rheinheimera perlucida;Rheinheimera tuosuensis [Bacteria] | 3 |
| 186 | WP_134057609.1 | co-chaperone GroES [Rheinheimera aquimaris] | 10369 | Rheinheimera aquimaris [Bacteria] | 3 |
| 187 | WP_008223663.1 | S9 family peptidase [Rheinheimera nanhaiensis] | 79165 | Rheinheimera nanhaiensis [Bacteria] | 3 |
| 188 | WP_027672159.1 | MULTISPECIES: 50S ribosomal protein L30 [Rheinheimera] | 6651 | Rheinheimera nanhaiensis;Rheinheimera sp. EpRS3;Rheinheimera aquimaris;Rheinheimera pacifica;Gammaproteobacteria bacterium HGW-Gammaproteobacteria-15;Rheinheimera baltica [Bacteria] | 3 |

| | | | | | |
|---|---|---|---|---|---|
| 189 | WP_097110231.1 | septum site-determining protein MinD [Rheinheimera tuosuensis] | 29380 | Rheinheimera tuosuensis [Bacteria] | 3 |
| 190 | WP_019675696.1 | aspartate aminotransferase family protein [Rheinheimera perlucida] | 44163 | Rheinheimera perlucida [Bacteria] | 3 |
| 190 | WP_068232787.1 | aspartate aminotransferase family protein [Rheinheimera sp. EpRS3] | 43732 | Rheinheimera sp. EpRS3 [Bacteria] | 2 |
| 190 | WP_134051993.1 | aspartate aminotransferase family protein [Rheinheimera aquimaris] | 43581 | Rheinheimera aquimaris [Bacteria] | 1 |
| 191 | WP_173500655.1 | fumarate hydratase [Rheinheimera sp. YQF-2] | 54949 | [] | 3 |
| 192 | WP_097110582.1 | glycoside hydrolase family 65 protein [Rheinheimera tuosuensis] | 85975 | Rheinheimera tuosuensis [Bacteria] | 3 |
| 193 | WP_092788892.1 | S9 family peptidase [Rheinheimera pacifica] | 75731 | Rheinheimera pacifica [Bacteria] | 3 |
| 194 | WP_127699261.1 | phosphoglycerate kinase [Rheinheimera riviphila] | 41032 | Rheinheimera sp. KYPC3 [Bacteria] | 3 |
| 195 | WP_097112077.1 | amidophosphoribosyltransferase [Rheinheimera tuosuensis] | 55710 | Rheinheimera tuosuensis [Bacteria] | 3 |
| 195 | WP_134056265.1 | amidophosphoribosyltransferase [Rheinheimera aquimaris] | 55585 | Rheinheimera aquimaris [Bacteria] | 1 |
| 196 | WP_097109879.1 | DUF1993 domain-containing protein [Rheinheimera tuosuensis] | 18815 | Rheinheimera tuosuensis [Bacteria] | 3 |
| 197 | WP_019677118.1 | nitrogen regulatory protein P-II [Rheinheimera perlucida] | 12694 | Rheinheimera perlucida [Bacteria] | 3 |
| 198 | WP_027670493.1 | recombinase RecA [Rheinheimera baltica] | 37261 | Rheinheimera baltica [Bacteria] | 3 |
| 199 | WP_019675421.1 | efflux RND transporter permease subunit [Rheinheimera perlucida] | 111702 | Rheinheimera perlucida [Bacteria] | 3 |
| 200 | WP_019677723.1 | MULTISPECIES: 50S ribosomal protein L16 [Rheinheimera] | 15573 | Rheinheimera perlucida;Rheinheimera tuosuensis [Bacteria] | 3 |
| 201 | WP_008898337.1 | PA2169 family four-helix-bundle protein [Rheinheimera sp. A13L] | 17316 | Rheinheimera sp. A13L [Bacteria] | 3 |
| 202 | WP_092792206.1 | succinylglutamate-semialdehyde dehydrogenase [Rheinheimera pacifica] | 52741 | Rheinheimera pacifica [Bacteria] | 3 |
| 203 | WP_132581375.1 | RNA polymerase sigma factor RpoD [Rheinheimera sp. D18] | 70324 | Rheinheimera sp. D18 [Bacteria] | 3 |

| | | | | | |
|---|---|---|---|---|---|
| 204 | WP_132585294.1 | bifunctional phosphoribosylaminoimidazolecarboxamide formyltransferase/IMP cyclohydrolase [Rheinheimera sp. D18] | 56731 | Rheinheimera sp. D18 [Bacteria] | 3 |
| 204 | WP_166839091.1 | bifunctional phosphoribosylaminoimidazolecarboxamide formyltransferase/IMP cyclohydrolase [Rheinheimera pleomorphica] | 56627 | [] | 1 |
| 205 | WP_019675986.1 | glutamate-1-semialdehyde 2,1-aminomutase [Rheinheimera perlucida] | 45390 | Rheinheimera perlucida [Bacteria] | 3 |
| 206 | WP_166837168.1 | malate dehydrogenase [Rheinheimera pleomorphica] | 45151 | [] | 3 |
| 207 | WP_068227543.1 | branched-chain amino acid aminotransferase [Rheinheimera sp. EpRS3] | 35758 | Rheinheimera sp. EpRS3 [Bacteria] | 3 |
| 208 | WP_097110579.1 | TonB-dependent receptor [Rheinheimera tuosuensis] | 82335 | Rheinheimera tuosuensis [Bacteria] | 3 |
| 209 | WP_132585357.1 | phosphate regulon transcriptional regulator PhoB [Rheinheimera sp. D18] | 26054 | Rheinheimera sp. D18 [Bacteria] | 3 |
| 210 | WP_173500441.1 | 3-oxoacid CoA-transferase subunit B [Rheinheimera sp. YQF-2] | 23279 | [] | 3 |
| 211 | WP_068067651.1 | MULTISPECIES: ubiquinol-cytochrome c reductase iron-sulfur subunit [Rheinheimera] | 21458 | Rheinheimera sp. KYPC3;Rheinheimera sp. SA_1 [Bacteria] | 3 |
| 212 | WP_134058318.1 | ribonuclease G [Rheinheimera aquimaris] | 55262 | Rheinheimera aquimaris [Bacteria] | 3 |
| 213 | WP_008898787.1 | type I-F CRISPR-associated protein Csy3 [Rheinheimera sp. A13L] | 36460 | Rheinheimera sp. A13L [Bacteria] | 3 |
| 214 | WP_166838050.1 | tyrosine--tRNA ligase [Rheinheimera pleomorphica] | 44729 | [] | 3 |
| 215 | WP_019675936.1 | 30S ribosomal protein S6 [Rheinheimera perlucida] | 15373 | Rheinheimera perlucida [Bacteria] | 3 |
| 216 | WP_092790145.1 | M13 family metallopeptidase [Rheinheimera pacifica] | 77268 | Rheinheimera pacifica [Bacteria] | 3 |
| 217 | WP_008899095.1 | MULTISPECIES: 50S ribosomal protein L17 [Chromatiaceae] | 15045 | Pararheinheimera tangshanensis;Rheinheimera sp. A13L;Rheinheimera sp. YQF- | 3 |

| | | | | | |
|---|---|---|---|---|---|
| | | | | 1;Pararheinheimera mesophila;Rheinheimera sp. KL1 [Bacteria] | |
| 218 | WP_173502004.1 | phosphoenolpyruvate carboxylase [Rheinheimera sp. YQF-2] | 97412 | [] | 3 |
| 218 | WP_166838099.1 | phosphoenolpyruvate carboxylase [Rheinheimera pleomorphica] | 97479 | [] | 1 |
| 219 | WP_092792039.1 | thioredoxin-disulfide reductase [Rheinheimera pacifica] | 34083 | Rheinheimera pacifica [Bacteria] | 2 |
| 220 | WP_166839293.1 | UDP-N-acetylglucosamine 1-carboxyvinyltransferase [Rheinheimera pleomorphica] | 44675 | [] | 2 |
| 221 | WP_092796070.1 | peptidylprolyl isomerase SurA [Rheinheimera pacifica] | 48299 | Rheinheimera pacifica [Bacteria] | 2 |
| 222 | WP_070049147.1 | carboxypeptidase family protein [Rheinheimera salexigens] | 42901 | Rheinheimera salexigens [Bacteria] | 2 |
| 223 | WP_097111416.1 | protein translocase subunit SecD [Rheinheimera tuosuensis] | 66815 | Rheinheimera tuosuensis [Bacteria] | 2 |
| 224 | WP_132581292.1 | 50S ribosomal protein L21 [Rheinheimera sp. D18] | 11399 | Rheinheimera sp. D18 [Bacteria] | 2 |
| 225 | WP_173502769.1 | 50S ribosomal protein L30 [Rheinheimera sp. YQF-2] | 6732 | [] | 2 |
| 226 | WP_068235207.1 | MULTISPECIES: 50S ribosomal protein L20 [Rheinheimera] | 13581 | Rheinheimera pacifica;Gammaproteobacteria bacterium HGW-Gammaproteobacteria-15;Rheinheimera sp. EpRS3 [Bacteria] | 2 |
| 227 | WP_127022056.1 | S41 family peptidase [Rheinheimera sp. LHK132] | 120174 | Rheinheimera sp. LHK132 [Bacteria] | 2 |
| 228 | WP_019676023.1 | elongation factor 4 [Rheinheimera perlucida] | 66757 | Rheinheimera perlucida [Bacteria] | 2 |
| 228 | WP_008223360.1 | elongation factor 4 [Rheinheimera nanhaiensis] | 66742 | Rheinheimera nanhaiensis [Bacteria] | 1 |
| 229 | WP_134057042.1 | Grx4 family monothiol glutaredoxin [Rheinheimera aquimaris] | 12746 | Rheinheimera aquimaris [Bacteria] | 2 |

| | | | | | |
|---|---|---|---|---|---|
| 229 | WP_027672031.1 | Grx4 family monothiol glutaredoxin [Rheinheimera baltica] | 12772 | Rheinheimera baltica [Bacteria] | 1 |
| 230 | WP_019676763.1 | M28 family peptidase [Rheinheimera perlucida] | 63340 | Rheinheimera perlucida [Bacteria] | 2 |
| 231 | WP_068228370.1 | outer membrane protein assembly factor BamB [Rheinheimera sp. EpRS3] | 42359 | Rheinheimera sp. EpRS3 [Bacteria] | 2 |
| 232 | WP_019674867.1 | acetyl-CoA carboxylase carboxyl transferase subunit alpha [Rheinheimera perlucida] | 35533 | Rheinheimera perlucida [Bacteria] | 2 |
| 233 | WP_166838286.1 | bifunctional UDP-N-acetylglucosamine diphosphorylase/glucosamine-1-phosphate N-acetyltransferase GlmU [Rheinheimera pleomorphica] | 48323 | [] | 2 |
| 234 | TXH95347.1 | elongation factor P [Rheinheimera sp.] | 20862 | Rheinheimera sp. [Bacteria] | 2 |
| 235 | WP_097112727.1 | RNA-binding protein [Rheinheimera tuosuensis] | 17550 | Rheinheimera tuosuensis [Bacteria] | 2 |
| 236 | WP_097110209.1 | glycine cleavage system aminomethyltransferase GcvT [Rheinheimera tuosuensis] | 38723 | Rheinheimera tuosuensis [Bacteria] | 2 |
| 236 | WP_134053976.1 | glycine cleavage system aminomethyltransferase GcvT [Rheinheimera aquimaris] | 38771 | Rheinheimera aquimaris [Bacteria] | 1 |
| 237 | WP_027672370.1 | glycine cleavage system protein GcvH [Rheinheimera baltica] | 13751 | Rheinheimera baltica [Bacteria] | 2 |
| 238 | WP_170948926.1 | S41 family peptidase [Rheinheimera tuosuensis] | 120813 | [] | 2 |
| 239 | WP_027670420.1 | orotate phosphoribosyltransferase [Rheinheimera baltica] | 23539 | Rheinheimera baltica [Bacteria] | 2 |
| 240 | WP_068065240.1 | alpha-D-glucose phosphate-specific phosphoglucomutase [Rheinheimera sp. SA_1] | 59033 | Rheinheimera sp. SA_1 [Bacteria] | 2 |
| 241 | WP_027669615.1 | type IV pilus twitching motility protein PilT [Rheinheimera baltica] | 38798 | Rheinheimera baltica [Bacteria] | 2 |
| 242 | WP_097112896.1 | cytochrome c oxidase subunit II [Rheinheimera tuosuensis] | 39650 | Rheinheimera tuosuensis [Bacteria] | 2 |
| 243 | WP_068067263.1 | aspartate carbamoyltransferase [Rheinheimera sp. SA_1] | 37457 | Rheinheimera sp. SA_1 [Bacteria] | 2 |
| 244 | TXH97699.1 | argininosuccinate synthase [Rheinheimera sp.] | 44802 | Rheinheimera sp. [Bacteria] | 2 |
| 245 | WP_092792807.1 | alpha-ketoacid dehydrogenase subunit beta [Rheinheimera pacifica] | 35910 | Rheinheimera pacifica [Bacteria] | 2 |

| | | | | | |
|---|---|---|---|---|---|
| 246 | WP_019675824.1 | serine hydrolase [Rheinheimera perlucida] | 42704 | Rheinheimera perlucida [Bacteria] | 2 |
| 247 | WP_134058424.1 | RNA-binding protein [Rheinheimera aquimaris] | 18130 | Rheinheimera aquimaris [Bacteria] | 2 |
| 248 | WP_173500924.1 | TonB-dependent receptor [Rheinheimera sp. YQF-2] | 101509 | [] | 2 |
| 248 | WP_097112045.1 | TonB-dependent receptor [Rheinheimera tuosuensis] | 95894 | Rheinheimera tuosuensis [Bacteria] | 1 |
| 248 | ALZ74505.1 | hypothetical protein ATY27_01175 [Rheinheimera sp. F8] | 107330 | Rheinheimera sp. F8 [Bacteria] | 1 |
| 249 | WP_008899940.1 | cytochrome bc complex cytochrome b subunit [Rheinheimera sp. A13L] | 48939 | Rheinheimera sp. A13L [Bacteria] | 2 |
| 250 | WP_134056313.1 | carbonate dehydratase [Rheinheimera aquimaris] | 25360 | Rheinheimera aquimaris [Bacteria] | 2 |
| 251 | WP_174978733.1 | alanine--tRNA ligase [Rheinheimera pleomorphica] | 95026 | [] | 2 |
| 252 | WP_127686946.1 | lipoyl synthase [Rheinheimera sp. YQF-1] | 37309 | Rheinheimera sp. YQF-1 [Bacteria] | 2 |
| 253 | WP_127685712.1 | UDP-glucose/GDP-mannose dehydrogenase family protein [Rheinheimera sp. YQF-1] | 49694 | Rheinheimera sp. YQF-1 [Bacteria] | 2 |
| 254 | WP_132585100.1 | protease modulator HflC [Rheinheimera sp. D18] | 32873 | Rheinheimera sp. D18 [Bacteria] | 2 |
| 254 | WP_147902987.1 | protease modulator HflC [Rheinheimera tangshanensis] | 33157 | Pararheinheimera tangshanensis [Bacteria] | 1 |
| 255 | WP_173501310.1 | 2-oxo acid dehydrogenase subunit E2 [Rheinheimera sp. YQF-2] | 55983 | [] | 2 |
| 256 | WP_166839439.1 | ferric iron uptake transcriptional regulator [Rheinheimera pleomorphica] | 16714 | [] | 2 |
| 256 | WP_097109700.1 | ferric iron uptake transcriptional regulator [Rheinheimera tuosuensis] | 16736 | Rheinheimera tuosuensis [Bacteria] | 1 |
| 257 | WP_092791972.1 | glutathione synthase [Rheinheimera pacifica] | 35467 | Rheinheimera pacifica [Bacteria] | 2 |
| 257 | WP_070049455.1 | glutathione synthase [Rheinheimera salexigens] | 35053 | Rheinheimera salexigens [Bacteria] | 1 |
| 257 | WP_173499740.1 | glutathione synthase [Rheinheimera sp. YQF-2] | 35341 | [] | 1 |
| 258 | WP_097112457.1 | urocanate hydratase [Rheinheimera tuosuensis] | 60663 | Rheinheimera tuosuensis [Bacteria] | 2 |
| 259 | WP_019674787.1 | 4a-hydroxytetrahydrobiopterin dehydratase [Rheinheimera perlucida] | 12995 | Rheinheimera perlucida [Bacteria] | 2 |
| 260 | WP_019677363.1 | methylcrotonoyl-CoA carboxylase [Rheinheimera perlucida] | 58397 | Rheinheimera perlucida [Bacteria] | 2 |

| | | | | | |
|---|---|---|---|---|---|
| 261 | WP_053423769.1 | adenylate kinase [Rheinheimera tangshanensis] | 23583 | Pararheinheimera tangshanensis;Rheinheimera sp. KL1 [Bacteria] | 2 |
| 261 | TXH95784.1 | adenylate kinase [Rheinheimera sp.] | 23439 | Rheinheimera sp. [Bacteria] | 1 |
| 262 | WP_127687266.1 | transketolase [Rheinheimera sp. YQF-1] | 72067 | Rheinheimera sp. YQF-1 [Bacteria] | 2 |
| 263 | WP_070049511.1 | MoxR family ATPase [Rheinheimera salexigens] | 34864 | Rheinheimera salexigens [Bacteria] | 2 |
| 264 | WP_070049983.1 | peptidoglycan-associated lipoprotein Pal [Rheinheimera salexigens] | 19639 | Rheinheimera salexigens [Bacteria] | 2 |
| 265 | WP_097111185.1 | fructose-bisphosphate aldolase class II [Rheinheimera tuosuensis] | 38458 | Rheinheimera tuosuensis [Bacteria] | 2 |
| 266 | WP_173501863.1 | nucleotide exchange factor GrpE [Rheinheimera sp. YQF-2] | 20593 | [] | 2 |
| 267 | ALZ74450.1 | translocation protein TolB [Rheinheimera sp. F8] | 49782 | Rheinheimera sp. F8 [Bacteria] | 2 |
| 268 | WP_008218637.1 | peroxiredoxin [Rheinheimera nanhaiensis] | 16726 | Rheinheimera nanhaiensis [Bacteria] | 2 |
| 269 | WP_134057654.1 | arginine/lysine/ornithine decarboxylase [Rheinheimera aquimaris] | 85002 | Rheinheimera aquimaris [Bacteria] | 2 |
| 269 | WP_070048423.1 | arginine/lysine/ornithine decarboxylase [Rheinheimera salexigens] | 85110 | Rheinheimera salexigens [Bacteria] | 1 |
| 270 | WP_097111978.1 | serine hydrolase [Rheinheimera tuosuensis] | 42809 | Rheinheimera tuosuensis [Bacteria] | 2 |
| 271 | WP_027672914.1 | 3-deoxy-7-phosphoheptulonate synthase class II [Rheinheimera baltica] | 50807 | Rheinheimera baltica [Bacteria] | 2 |
| 272 | WP_068231084.1 | organic hydroperoxide resistance protein [Rheinheimera sp. EpRS3] | 14264 | Rheinheimera sp. EpRS3 [Bacteria] | 2 |
| 273 | WP_031570995.1 | HTH-type transcriptional regulator CysB [Pararheinheimera texasensis] | 36476 | Rheinheimera sp.;Pararheinheimera texasensis [Bacteria] | 2 |
| 274 | WP_008219722.1 | MULTISPECIES: 50S ribosomal protein L33 [Gammaproteobacteria] | 6056 | Pararheinheimera mesophila;Rheinheimera sp. KL1;Alishewanella sp. 32-51-5;Rheinheimera perlucida;Alishewanella jeotgali;Rheinheimera pacifica;Rheinheimera salexigens;Rheinheimera nanhaiensis;Rheinheimera sp. | 2 |

| | | | | | |
|---|---|---|---|---|---|
| | | | | EpRS3;Rheinheimera aquimaris;Rheinheimera sp. LHK132;Rheinheimera sp. A13L;Pararheinheimera tangshanensis;Alishewanella aestuarii;Alishewanella sp. WH16-1;Rheinheimera sp. YQF-1;Alkalimonas amylolytica;Gammaproteobacteria bacterium HGW-Gammaproteobacteria-15;Rheinheimera baltica;Arsukibacterium sp. MJ3;Arsukibacterium ikkense;Rheinheimera tuosuensis;Alishewanella agri;Alishewanella sp. HH-ZS;Rheinheimera sp. D18 [Bacteria] | |
| 275 | WP_173502698.1 | TonB-dependent receptor [Rheinheimera sp. YQF-2] | 79806 | [] | 2 |
| 276 | WP_008223040.1 | beta-ketoacyl-ACP synthase II [Rheinheimera nanhaiensis] | 43272 | Rheinheimera nanhaiensis [Bacteria] | 2 |
| 277 | WP_127023916.1 | DUF3450 domain-containing protein [Rheinheimera sp. LHK132] | 28443 | Rheinheimera sp. LHK132 [Bacteria] | 2 |
| 278 | WP_019676236.1 | DNA polymerase III subunit beta [Rheinheimera perlucida] | 41024 | Rheinheimera perlucida [Bacteria] | 2 |
| 279 | WP_046520585.1 | MULTISPECIES: YbaB/EbfC family nucleoid-associated protein [Chromatiaceae] | 11844 | Pararheinheimera tangshanensis;Rheinheimera sp. LHK132;Pararheinheimera mesophila [Bacteria] | 2 |
| 280 | WP_097109656.1 | bifunctional 3-hydroxydecanoyl-ACP dehydratase/trans-2-decenoyl-ACP isomerase [Rheinheimera tuosuensis] | 18709 | Rheinheimera tuosuensis [Bacteria] | 2 |
| 281 | WP_132581837.1 | carbamoyl-phosphate synthase large subunit [Rheinheimera sp. D18] | 117759 | Rheinheimera sp. D18 [Bacteria] | 2 |

| | | | | | |
|---|---|---|---|---|---|
| 282 | TXH97517.1 | S9 family peptidase [Rheinheimera sp.] | 70448 | Rheinheimera sp. [Bacteria] | 2 |
| 283 | WP_097110236.1 | long-chain-fatty-acid--CoA ligase FadD [Rheinheimera tuosuensis] | 61167 | Rheinheimera tuosuensis [Bacteria] | 2 |
| 284 | WP_173502569.1 | membrane protein insertase YidC [Rheinheimera sp. YQF-2] | 61127 | [] | 2 |
| 285 | WP_008217876.1 | class 1 fructose-bisphosphatase [Rheinheimera nanhaiensis] | 35423 | Rheinheimera nanhaiensis [Bacteria] | 2 |
| 286 | ALZ77269.1 | acetylornithine aminotransferase [Rheinheimera sp. F8] | 43382 | Rheinheimera sp. F8 [Bacteria] | 2 |
| 287 | WP_132586156.1 | type II secretion system secretin GspD [Rheinheimera sp. D18] | 76929 | Rheinheimera sp. D18 [Bacteria] | 2 |
| 288 | WP_008897788.1 | aminoacyl-histidine dipeptidase [Rheinheimera sp. A13L] | 52645 | Rheinheimera sp. A13L [Bacteria] | 2 |
| 289 | TXH93891.1 | acetate--CoA ligase [Rheinheimera sp.] | 71950 | Rheinheimera sp. [Bacteria] | 2 |
| 289 | WP_097109829.1 | acetate--CoA ligase [Rheinheimera tuosuensis] | 72157 | Rheinheimera tuosuensis [Bacteria] | 1 |
| 290 | WP_019674651.1 | UDP-N-acetyl-D-mannosamine dehydrogenase [Rheinheimera perlucida] | 45499 | Rheinheimera perlucida [Bacteria] | 2 |
| 291 | WP_166838722.1 | protein TolQ [Rheinheimera pleomorphica] | 24831 | [] | 2 |
| 292 | WP_097112070.1 | aspartate-semialdehyde dehydrogenase [Rheinheimera tuosuensis] | 36783 | Rheinheimera tuosuensis [Bacteria] | 2 |
| 293 | WP_026349364.1 | Tol-Pal system protein TolB [Rheinheimera perlucida] | 49362 | Rheinheimera perlucida [Bacteria] | 2 |
| 294 | WP_070050819.1 | MotA/TolQ/ExbB proton channel family protein [Rheinheimera salexigens] | 19464 | Rheinheimera salexigens [Bacteria] | 2 |
| 295 | WP_097109672.1 | quinone-dependent dihydroorotate dehydrogenase [Rheinheimera tuosuensis] | 36534 | Rheinheimera tuosuensis [Bacteria] | 2 |
| 296 | WP_068062114.1 | cell division protein FtsA [Rheinheimera sp. SA_1] | 44678 | Rheinheimera sp. SA_1 [Bacteria] | 2 |
| 297 | WP_127698383.1 | glycine--tRNA ligase subunit alpha [Rheinheimera riviphila] | 35302 | Rheinheimera sp. KYPC3 [Bacteria] | 2 |
| 298 | WP_147903685.1 | S9 family peptidase [Rheinheimera tangshanensis] | 75204 | Pararheinheimera tangshanensis [Bacteria] | 2 |
| 299 | WP_008220344.1 | glutamate-5-semialdehyde dehydrogenase [Rheinheimera nanhaiensis] | 45146 | Rheinheimera nanhaiensis [Bacteria] | 2 |

| | | | | | |
|---|---|---|---|---|---|
| 300 | WP_097111926.1 | DUF883 family protein [Rheinheimera tuosuensis] | 11372 | Rheinheimera tuosuensis [Bacteria] | 2 |
| 301 | WP_166839617.1 | cytochrome-c oxidase, cbb3-type subunit III [Rheinheimera pleomorphica] | 36086 | [] | 2 |
| 302 | WP_070050185.1 | ATP-binding protein [Rheinheimera salexigens] | 88597 | Rheinheimera salexigens [Bacteria] | 2 |
| 303 | WP_097110947.1 | phosphoribosylaminoimidazolesuccinocarboxamide synthase [Rheinheimera tuosuensis] | 26903 | Rheinheimera tuosuensis [Bacteria] | 2 |
| 304 | WP_019675470.1 | phosphoglycerate dehydrogenase [Rheinheimera perlucida] | 44925 | Rheinheimera perlucida [Bacteria] | 1 |
| 305 | WP_070048755.1 | 23S rRNA pseudouridine(1911/1915/1917) synthase RluD [Rheinheimera salexigens] | 36246 | Rheinheimera salexigens [Bacteria] | 1 |
| 306 | WP_068234596.1 | MULTISPECIES: flagellar motor switch protein FliN [Rheinheimera] | 14216 | Rheinheimera pacifica;Gammaproteobacteria bacterium HGW-Gammaproteobacteria-15;Rheinheimera sp. EpRS3 [Bacteria] | 1 |
| 307 | WP_068065319.1 | ribonucleoside-diphosphate reductase subunit alpha [Rheinheimera sp. SA_1] | 86331 | Rheinheimera sp. SA_1 [Bacteria] | 1 |
| 308 | WP_070049821.1 | chemotaxis response regulator CheY [Rheinheimera salexigens] | 14042 | Rheinheimera salexigens [Bacteria] | 1 |
| 309 | TXH98801.1 | methionyl-tRNA formyltransferase [Rheinheimera sp.] | 34520 | Rheinheimera sp. [Bacteria] | 1 |
| 310 | WP_046559227.1 | MULTISPECIES: preprotein translocase subunit SecY [Chromatiaceae] | 48442 | Rheinheimera tuosuensis;Arsukibacterium ikkense [Bacteria] | 1 |
| 311 | WP_097112818.1 | bifunctional demethylmenaquinone methyltransferase/2-methoxy-6-polyprenyl-1,4-benzoquinol methylase UbiE [Rheinheimera tuosuensis] | 27867 | Rheinheimera tuosuensis [Bacteria] | 1 |
| 312 | WP_027669342.1 | flotillin family protein [Rheinheimera baltica] | 63753 | Rheinheimera baltica [Bacteria] | 1 |
| 313 | WP_166837091.1 | TIGR04219 family outer membrane beta-barrel protein [Rheinheimera pleomorphica] | 27122 | [] | 1 |
| 314 | WP_019677637.1 | Si-specific NAD(P)(+) transhydrogenase [Rheinheimera perlucida] | 52437 | Rheinheimera perlucida [Bacteria] | 1 |

| | | | | | |
|---|---|---|---|---|---|
| 315 | WP_019674227.1 | S41 family peptidase [Rheinheimera perlucida] | 119407 | Rheinheimera perlucida [Bacteria] | 1 |
| 316 | WP_019675353.1 | outer membrane protein assembly factor BamD [Rheinheimera perlucida] | 28410 | Rheinheimera perlucida [Bacteria] | 1 |
| 317 | WP_166838811.1 | acetyl-CoA carboxylase carboxyl transferase subunit alpha [Rheinheimera pleomorphica] | 35514 | [] | 1 |
| 318 | WP_027672413.1 | hypothetical protein [Rheinheimera baltica] | 11596 | Rheinheimera baltica [Bacteria] | 1 |
| 319 | WP_097111943.1 | mandelate racemase/muconate lactonizing enzyme family protein [Rheinheimera tuosuensis] | 44017 | Rheinheimera tuosuensis [Bacteria] | 1 |
| 320 | ALZ75429.1 | tryptophan halogenase [Rheinheimera sp. F8] | 56986 | Rheinheimera sp. F8 [Bacteria] | 1 |
| 321 | WP_027669592.1 | 4-hydroxy-tetrahydrodipicolinate synthase [Rheinheimera baltica] | 34952 | Rheinheimera baltica [Bacteria] | 1 |
| 322 | WP_083256570.1 | ribosome maturation factor RimM [Rheinheimera salexigens] | 19966 | Rheinheimera salexigens [Bacteria] | 1 |
| 323 | WP_147905362.1 | carboxypeptidase [Rheinheimera tangshanensis] | 58030 | Pararheinheimera tangshanensis [Bacteria] | 1 |
| 324 | WP_019674568.1 | bifunctional GNAT family N-acetyltransferase/carbon-nitrogen hydrolase family protein [Rheinheimera perlucida] | 58938 | Rheinheimera perlucida [Bacteria] | 1 |
| 325 | WP_127025682.1 | TIGR00153 family protein [Rheinheimera sp. LHK132] | 25800 | Rheinheimera sp. LHK132 [Bacteria] | 1 |
| 326 | WP_127024581.1 | aspartate-semialdehyde dehydrogenase [Rheinheimera sp. LHK132] | 36979 | Rheinheimera sp. LHK132 [Bacteria] | 1 |
| 327 | WP_127698990.1 | 3-phosphoshikimate 1-carboxyvinyltransferase [Rheinheimera riviphila] | 46570 | Rheinheimera sp. KYPC3 [Bacteria] | 1 |
| 328 | WP_170948962.1 | prolyl oligopeptidase family serine peptidase [Rheinheimera tuosuensis] | 87412 | [] | 1 |
| 329 | WP_070049179.1 | protease HtpX [Rheinheimera salexigens] | 30935 | Rheinheimera salexigens [Bacteria] | 1 |
| 330 | WP_134053428.1 | F0F1 ATP synthase subunit delta [Rheinheimera aquimaris] | 19108 | Rheinheimera aquimaris [Bacteria] | 1 |
| 331 | WP_019675953.1 | class 1 fructose-bisphosphatase [Rheinheimera perlucida] | 35314 | Rheinheimera perlucida [Bacteria] | 1 |
| 332 | WP_008221515.1 | bifunctional 4-hydroxy-2-oxoglutarate aldolase/2-dehydro-3-deoxy-phosphogluconate aldolase [Rheinheimera nanhaiensis] | 22589 | Rheinheimera nanhaiensis [Bacteria] | 1 |

| | | | | | |
|---|---|---|---|---|---|
| 333 | WP_027671199.1 | bifunctional demethylmenaquinone methyltransferase/2-methoxy-6-polyprenyl-1,4-benzoquinol methylase UbiE [Rheinheimera baltica] | 27754 | Rheinheimera baltica [Bacteria] | 1 |
| 334 | WP_166839071.1 | histidine--tRNA ligase [Rheinheimera pleomorphica] | 47389 | [] | 1 |
| 335 | WP_070048405.1 | phosphoribosylamine--glycine ligase [Rheinheimera salexigens] | 44835 | Rheinheimera salexigens [Bacteria] | 1 |
| 336 | WP_019677274.1 | S9 family peptidase [Rheinheimera perlucida] | 71348 | Rheinheimera perlucida [Bacteria] | 1 |
| 337 | WP_040551545.1 | S-(hydroxymethyl)glutathione dehydrogenase/class III alcohol dehydrogenase [Rheinheimera nanhaiensis] | 40223 | Rheinheimera nanhaiensis [Bacteria] | 1 |
| 338 | WP_097109919.1 | EAL domain-containing protein [Rheinheimera tuosuensis] | 73171 | Rheinheimera tuosuensis [Bacteria] | 1 |
| 339 | WP_134051818.1 | trans-2-enoyl-CoA reductase family protein [Rheinheimera aquimaris] | 43571 | Rheinheimera aquimaris [Bacteria] | 1 |
| 340 | WP_127688037.1 | EAL domain-containing protein [Rheinheimera sp. YQF-1] | 113355 | Rheinheimera sp. YQF-1 [Bacteria] | 1 |
| 341 | WP_027670597.1 | TonB-dependent receptor [Rheinheimera baltica] | 109866 | Rheinheimera baltica [Bacteria] | 1 |
| 342 | WP_173502751.1 | arginine--tRNA ligase [Rheinheimera sp. YQF-2] | 64317 | [] | 1 |
| 343 | WP_092793556.1 | ketoacyl-ACP synthase III [Rheinheimera pacifica] | 34760 | Rheinheimera pacifica [Bacteria] | 1 |
| 344 | WP_092789282.1 | YicC family protein [Rheinheimera pacifica] | 32803 | Rheinheimera pacifica [Bacteria] | 1 |
| 345 | WP_097109552.1 | methionine--tRNA ligase [Rheinheimera tuosuensis] | 76665 | Rheinheimera tuosuensis [Bacteria] | 1 |
| 346 | WP_019674751.1 | TonB-dependent receptor [Rheinheimera perlucida] | 66941 | Rheinheimera perlucida [Bacteria] | 1 |
| 347 | WP_127025267.1 | leucine--tRNA ligase [Rheinheimera sp. LHK132] | 97454 | Rheinheimera sp. LHK132 [Bacteria] | 1 |
| 348 | WP_097109635.1 | hypothetical protein [Rheinheimera tuosuensis] | 46919 | Rheinheimera tuosuensis [Bacteria] | 1 |
| 349 | WP_070050706.1 | amidohydrolase family protein [Rheinheimera salexigens] | 112903 | Rheinheimera salexigens [Bacteria] | 1 |
| 350 | WP_019676511.1 | polyphosphate kinase 1 [Rheinheimera perlucida] | 82973 | Rheinheimera perlucida [Bacteria] | 1 |

| | | | | | |
|---|---|---|---|---|---|
| 351 | WP_053423287.1 | Rsd/AlgQ family anti-sigma factor [Rheinheimera tangshanensis] | 18485 | Pararheinheimera tangshanensis;Rheinheimera sp. KL1 [Bacteria] | 1 |
| 352 | WP_092796196.1 | TonB-dependent receptor [Rheinheimera pacifica] | 113656 | Rheinheimera pacifica [Bacteria] | 1 |
| 353 | WP_068234709.1 | SDR family oxidoreductase [Rheinheimera sp. EpRS3] | 36825 | Rheinheimera sp. EpRS3 [Bacteria] | 1 |
| 354 | WP_070048109.1 | DUF1801 domain-containing protein [Rheinheimera salexigens] | 15000 | Rheinheimera salexigens [Bacteria] | 1 |
| 355 | WP_166839890.1 | TonB family protein [Rheinheimera pleomorphica] | 22120 | [] | 1 |
| 356 | WP_178089525.1 | phosphoribosylformylglycinamidine cyclo-ligase [Rheinheimera sp. YQF-2] | 39413 | [] | 1 |
| 357 | WP_166837794.1 | 4-hydroxyproline epimerase [Rheinheimera pleomorphica] | 36327 | [] | 1 |
| 358 | TXH94245.1 | BON domain-containing protein [Rheinheimera sp.] | 19946 | Rheinheimera sp. [Bacteria] | 1 |
| 359 | WP_027672243.1 | MULTISPECIES: 50S ribosomal protein L35 [Rheinheimera] | 7405 | Rheinheimera perlucida;Rheinheimera tuosuensis;Rheinheimera sp. EpRS3;Rheinheimera aquimaris;Rheinheimera pacifica;Gammaproteobacteria bacterium HGW-Gammaproteobacteria-15;Rheinheimera baltica [Bacteria] | 1 |
| 360 | TXH93871.1 | 3,4-dihydroxy-2-butanone-4-phosphate synthase [Rheinheimera sp.] | 40595 | Rheinheimera sp. [Bacteria] | 1 |
| 361 | WP_173502185.1 | ATP-dependent protease ATP-binding subunit ClpX [Rheinheimera sp. YQF-2] | 47489 | [] | 1 |
| 362 | WP_178089464.1 | GNAT family N-acetyltransferase [Rheinheimera sp. YQF-2] | 17025 | [] | 1 |
| 363 | WP_068236877.1 | type II secretion system major pseudopilin GspG [Rheinheimera sp. EpRS3] | 16679 | Rheinheimera sp. EpRS3 [Bacteria] | 1 |
| 364 | ALZ75427.1 | TonB-dependent receptor [Rheinheimera sp. F8] | 111736 | Rheinheimera sp. F8 [Bacteria] | 1 |
| 365 | WP_134051735.1 | ATP-dependent RNA helicase SrmB [Rheinheimera aquimaris] | 45899 | Rheinheimera aquimaris [Bacteria] | 1 |

| | | | | | |
|---|---|---|---|---|---|
| 366 | WP_008222562.1 | TonB-dependent receptor [Rheinheimera nanhaiensis] | 82184 | Rheinheimera nanhaiensis [Bacteria] | 1 |
| 367 | WP_008222127.1 | MULTISPECIES: phage tail protein [Rheinheimera] | 16929 | Rheinheimera nanhaiensis [Bacteria] | 1 |
| 368 | WP_127697683.1 | UDP-glucose/GDP-mannose dehydrogenase family protein [Rheinheimera riviphila] | 49124 | Rheinheimera sp. KYPC3 [Bacteria] | 1 |
| 369 | WP_008223878.1 | 30S ribosomal protein S15 [Rheinheimera nanhaiensis] | 10061 | Rheinheimera nanhaiensis [Bacteria] | 1 |
| 370 | TXH94227.1 | UDP-N-acetylmuramate--L-alanine ligase [Rheinheimera sp.] | 52333 | Rheinheimera sp. [Bacteria] | 1 |
| 371 | WP_068231434.1 | succinate dehydrogenase, hydrophobic membrane anchor protein [Rheinheimera sp. EpRS3] | 12785 | Rheinheimera sp. EpRS3 [Bacteria] | 1 |
| 372 | WP_068230110.1 | TonB-dependent receptor [Rheinheimera sp. EpRS3] | 65745 | Rheinheimera sp. EpRS3 [Bacteria] | 1 |
| 373 | WP_027672901.1 | S41 family peptidase [Rheinheimera baltica] | 121912 | Rheinheimera baltica [Bacteria] | 1 |
| 374 | WP_134052692.1 | cell division protein ZapE [Rheinheimera aquimaris] | 42692 | Rheinheimera aquimaris [Bacteria] | 1 |
| 375 | WP_132582483.1 | peptidylprolyl isomerase [Rheinheimera sp. D18] | 18089 | Rheinheimera sp. D18 [Bacteria] | 1 |
| 376 | WP_127687319.1 | S9 family peptidase [Rheinheimera sp. YQF-1] | 80357 | Rheinheimera sp. YQF-1 [Bacteria] | 1 |
| 377 | WP_092791876.1 | efflux RND transporter permease subunit [Rheinheimera pacifica] | 118079 | Rheinheimera pacifica [Bacteria] | 1 |
| 378 | WP_173501645.1 | 4-hydroxythreonine-4-phosphate dehydrogenase PdxA [Rheinheimera sp. YQF-2] | 35386 | [] | 1 |
| 379 | WP_068066361.1 | phosphoenolpyruvate--protein phosphotransferase [Rheinheimera sp. SA_1] | 84514 | Rheinheimera sp. SA_1 [Bacteria] | 1 |
| 380 | WP_068066140.1 | transcription antitermination factor NusB [Rheinheimera sp. SA_1] | 15599 | Rheinheimera sp. SA_1 [Bacteria] | 1 |
| 381 | WP_027671519.1 | beta-aspartyl-peptidase [Rheinheimera baltica] | 41474 | Rheinheimera baltica [Bacteria] | 1 |
| 382 | WP_097111034.1 | acyl-CoA dehydrogenase [Rheinheimera tuosuensis] | 84673 | Rheinheimera tuosuensis [Bacteria] | 1 |
| 383 | WP_027671521.1 | TonB-dependent receptor [Rheinheimera baltica] | 105153 | Rheinheimera baltica [Bacteria] | 1 |
| 384 | KOO57451.1 | acetyl-CoA carboxylase subunit alpha [Rheinheimera sp. KL1] | 35540 | [] | 1 |

| | | | | | |
|---|---|---|---|---|---|
| 385 | WP_068230554.1 | DUF4097 family beta strand repeat protein [Rheinheimera sp. EpRS3] | 34074 | Rheinheimera sp. EpRS3 [Bacteria] | 1 |
| 386 | WP_037055692.1 | TonB family protein [Rheinheimera baltica] | 21387 | [] | 1 |
| 387 | WP_166835973.1 | signal recognition particle protein [Rheinheimera pleomorphica] | 49447 | [] | 1 |
| 388 | WP_097111131.1 | response regulator transcription factor [Rheinheimera tuosuensis] | 25667 | Rheinheimera tuosuensis [Bacteria] | 1 |
| 389 | WP_008219734.1 | MULTISPECIES: ribonuclease PH [Rheinheimera] | 25938 | Rheinheimera nanhaiensis;Rheinheimera aquimaris [Bacteria] | 1 |
| 390 | WP_147903958.1 | valine--tRNA ligase [Rheinheimera tangshanensis] | 108804 | Pararheinheimera tangshanensis [Bacteria] | 1 |
| 391 | WP_019676700.1 | carboxypeptidase regulatory-like domain-containing protein [Rheinheimera perlucida] | 114519 | Rheinheimera perlucida [Bacteria] | 1 |
| 392 | WP_068229786.1 | proline--tRNA ligase [Rheinheimera sp. EpRS3] | 64040 | Rheinheimera sp. EpRS3 [Bacteria] | 1 |
| 393 | WP_147903944.1 | DNA topoisomerase IV subunit A [Rheinheimera tangshanensis] | 83901 | Pararheinheimera tangshanensis [Bacteria] | 1 |
| 394 | WP_173499275.1 | aminopeptidase PepB [Rheinheimera sp. YQF-2] | 45390 | [] | 1 |
| 395 | WP_097109571.1 | NAD(P)H nitroreductase [Rheinheimera tuosuensis] | 20220 | Rheinheimera tuosuensis [Bacteria] | 1 |
| 396 | WP_008900479.1 | arginine N-succinyltransferase [Rheinheimera sp. A13L] | 38144 | Rheinheimera sp. A13L [Bacteria] | 1 |
| 397 | WP_134056651.1 | RNA polymerase sigma factor RpoE [Rheinheimera aquimaris] | 21720 | Rheinheimera aquimaris [Bacteria] | 1 |
| 398 | WP_173501792.1 | carboxypeptidase [Rheinheimera sp. YQF-2] | 56944 | [] | 1 |
| 399 | WP_070049210.1 | 3-phosphoserine/phosphohydroxythreonine transaminase [Rheinheimera salexigens] | 40094 | Rheinheimera salexigens [Bacteria] | 1 |
| 400 | WP_008220244.1 | chorismate mutase [Rheinheimera nanhaiensis] | 42973 | Rheinheimera nanhaiensis [Bacteria] | 1 |
| 401 | WP_092793639.1 | 3-oxoacyl-ACP reductase FabG [Rheinheimera pacifica] | 25881 | Rheinheimera pacifica [Bacteria] | 1 |
| 402 | TXH95464.1 | translation initiation factor IF-3 [Rheinheimera sp.] | 20792 | Rheinheimera sp. [Bacteria] | 1 |
| 403 | WP_132581954.1 | xanthine phosphoribosyltransferase [Rheinheimera sp. D18] | 17537 | Rheinheimera sp. D18 [Bacteria] | 1 |

| | | | | | |
|---|---|---|---|---|---|
| 404 | WP_127699279.1 | ATP-dependent Clp endopeptidase proteolytic subunit ClpP [Rheinheimera riviphila] | 22508 | Rheinheimera sp. KYPC3 [Bacteria] | 1 |
| 405 | WP_008220880.1 | LPS export ABC transporter ATP-binding protein [Rheinheimera nanhaiensis] | 26495 | Rheinheimera nanhaiensis [Bacteria] | 1 |
| 406 | WP_092790384.1 | DUF481 domain-containing protein [Rheinheimera pacifica] | 26823 | Rheinheimera pacifica [Bacteria] | 1 |
| 407 | WP_008221586.1 | integration host factor subunit beta [Rheinheimera nanhaiensis] | 10733 | Rheinheimera nanhaiensis [Bacteria] | 1 |
| 408 | WP_008218627.1 | glutathione-disulfide reductase [Rheinheimera nanhaiensis] | 48578 | Rheinheimera nanhaiensis [Bacteria] | 1 |
| 409 | WP_068232357.1 | 3-hydroxyacyl-ACP dehydratase FabZ [Rheinheimera sp. EpRS3] | 17162 | Rheinheimera sp. EpRS3 [Bacteria] | 1 |
| 410 | WP_027671253.1 | DEAD/DEAH box helicase family protein [Rheinheimera baltica] | 122174 | Rheinheimera baltica [Bacteria] | 1 |
| 411 | WP_008220406.1 | N-succinylarginine dihydrolase [Rheinheimera nanhaiensis] | 49756 | Rheinheimera nanhaiensis [Bacteria] | 1 |
| 412 | WP_127685126.1 | phosphatidylserine decarboxylase [Rheinheimera sp. YQF-1] | 31341 | Rheinheimera sp. YQF-1 [Bacteria] | 1 |
| 413 | WP_019676229.1 | YbhB/YbcL family Raf kinase inhibitor-like protein [Rheinheimera perlucida] | 17643 | Rheinheimera perlucida [Bacteria] | 1 |
| 414 | WP_068229467.1 | OsmC family protein [Rheinheimera sp. EpRS3] | 15506 | Rheinheimera sp. EpRS3 [Bacteria] | 1 |
| 415 | WP_008222163.1 | transcriptional repressor LexA [Rheinheimera nanhaiensis] | 22636 | [] | 1 |
| 416 | WP_092793851.1 | DNA translocase FtsK 4TM domain-containing protein [Rheinheimera pacifica] | 89477 | Rheinheimera pacifica;Gammaproteobacteria bacterium HGW-Gammaproteobacteria-15 [Bacteria] | 1 |
| 417 | WP_134053937.1 | hypothetical protein [Rheinheimera aquimaris] | 75196 | Rheinheimera aquimaris [Bacteria] | 1 |
| 418 | WP_008223049.1 | MULTISPECIES: 50S ribosomal protein L32 [Rheinheimera] | 6203 | Rheinheimera nanhaiensis;Rheinheimera sp. EpRS3;Rheinheimera aquimaris;Rheinheimera pacifica;Gammaproteobacteria bacterium HGW- | 1 |

| | | | | | |
|---|---|---|---|---|---|
| | | | | Gammaproteobacteria-15;Rheinheimera baltica;Rheinheimera sp. D18 [Bacteria] | |
| 419 | WP_068237935.1 | ribonucleoside-diphosphate reductase subunit alpha [Rheinheimera sp. EpRS3] | 86618 | Rheinheimera sp. EpRS3 [Bacteria] | 1 |
| 420 | WP_097109681.1 | carboxy terminal-processing peptidase [Rheinheimera tuosuensis] | 75969 | Rheinheimera tuosuensis [Bacteria] | 1 |
| 421 | WP_070049327.1 | cytochrome-c oxidase, cbb3-type subunit II [Rheinheimera salexigens] | 23027 | Rheinheimera salexigens [Bacteria] | 1 |
| 422 | WP_097111151.1 | UDP-glucose/GDP-mannose dehydrogenase family protein [Rheinheimera tuosuensis] | 48306 | Rheinheimera tuosuensis [Bacteria] | 1 |
| 423 | WP_019676987.1 | MULTISPECIES: DUF2750 domain-containing protein [Chromatiaceae] | 13406 | Rheinheimera perlucida;Arsukibacterium ikkense [Bacteria] | 1 |
| 424 | TXH98583.1 | bifunctional 23S rRNA (guanine(2069)-N(7))-methyltransferase RlmK/23S rRNA (guanine(2445)-N(2))-methyltransferase RlmL [Rheinheimera sp.] | 80272 | Rheinheimera sp. [Bacteria] | 1 |
| 425 | WP_132582609.1 | YajQ family cyclic di-GMP-binding protein [Rheinheimera sp. D18] | 18228 | Rheinheimera sp. D18 [Bacteria] | 1 |
| 426 | WP_097112507.1 | phosphoribosylamine--glycine ligase [Rheinheimera tuosuensis] | 45086 | Rheinheimera tuosuensis [Bacteria] | 1 |
| 427 | WP_127026272.1 | excinuclease ABC subunit UvrA [Rheinheimera sp. LHK132] | 104864 | Rheinheimera sp. LHK132 [Bacteria] | 1 |
| 428 | ALZ77049.1 | chain-length determining protein [Rheinheimera sp. F8] | 80316 | Rheinheimera sp. F8 [Bacteria] | 1 |
| 429 | WP_127019586.1 | ABC transporter ATP-binding protein [Rheinheimera sp. LHK132] | 31940 | Rheinheimera sp. LHK132 [Bacteria] | 1 |
| 430 | WP_166839441.1 | flavodoxin FldA [Rheinheimera pleomorphica] | 20116 | [] | 1 |
| 431 | WP_027672326.1 | hypothetical protein [Rheinheimera baltica] | 7916 | Rheinheimera baltica [Bacteria] | 1 |
| 432 | WP_008218129.1 | acetyl/propionyl/methylcrotonyl-CoA carboxylase subunit alpha [Rheinheimera nanhaiensis] | 72902 | Rheinheimera nanhaiensis [Bacteria] | 1 |

| | | | | | |
|---|---|---|---|---|---|
| 433 | WP_092789931.1 | SRPBCC domain-containing protein [Rheinheimera pacifica] | 16738 | Rheinheimera pacifica [Bacteria] | 1 |
| 434 | WP_092789362.1 | bacterioferritin [Rheinheimera pacifica] | 18174 | Rheinheimera pacifica [Bacteria] | 1 |
| 435 | WP_068059494.1 | sigma 54-interacting transcriptional regulator [Rheinheimera sp. SA_1] | 49967 | Rheinheimera sp. SA_1 [Bacteria] | 1 |
| 436 | WP_019676738.1 | integration host factor subunit alpha [Rheinheimera perlucida] | 10949 | Rheinheimera perlucida [Bacteria] | 1 |
| 437 | WP_068234858.1 | enoyl-CoA hydratase [Rheinheimera sp. EpRS3] | 27568 | Rheinheimera sp. EpRS3 [Bacteria] | 1 |
| 438 | WP_004335261.1 | MULTISPECIES: F0F1 ATP synthase subunit C [Gammaproteobacteria] | 8003 | Pseudoalteromonas sp. S410;Pseudoalteromonas sp. S1609;Pararheinheimera mesophila;Pseudoalteromonas sp. A25;Pseudoalteromonas sp. S3173;Pseudoalteromonas sp. S3260;Colwellia sp. MT41;Pseudoalteromonas phenolica;Pseudoalteromonas aurantia;Pseudoalteromonas aliena;Pseudoalteromonas marina;Pseudoalteromonas sp. TB64;Pseudoalteromonas sp. 13-15;Rheinheimera sp. KL1;Pseudoalteromonas sp. MSK9-3;Pseudoalteromonas sp. CO133X;Pseudoalteromonas sp. 10-33;Pseudoalteromonas sp. S4741;Pseudoalteromonas sp. S409;Pseudoalteromonas luteoviolacea;Pseudoalteromonas sp. H100;Pseudoalteromonas sp. ND6B;Pseudoalteromonas sp. S3431;Pararheinheimera tangshanensis;Pseudoalteromonas sp. S186;Pseudoalteromonas spiralis;Pseudoalteromonas sp. | 1 |

| | | | | | |
|---|---|---|---|---|---|
| | | | | CO109Y;Pseudoalteromonas sp. BSi20495;Pseudoalteromonas sp. DSM 26666;Pseudoalteromonas sp. 1_2015MBL_MicDiv;Colwellia sp. MT2012;Pseudoalteromonas sp. S407;Pseudoalteromonas sp. S554;Rheinheimera sp. A13L;Colwellia marinimaniae;Pseudoalteromonas sp. S408;Pseudoalteromonas sp. A2;Pseudoalteromonas fuliginea;Pseudoalteromonas sp. PAMC 22718;Pseudoalteromonas sp. S4491;Pseudoalteromonas sp. SCSIO_11900;Pseudoalteromonas citrea;Pseudoalteromonas lipolytica;Rheinheimera sp. YQF-1;Pseudoalteromonas shioyasakiensis;Pseudoalteromonas sp. S558;Pseudoalteromonas sp. FUC4;Pseudoalteromonas sp. PAB 2.2;Pseudoalteromonas sp. UCD-33C;Pseudoalteromonas sp. SK20;Pseudoalteromonas tetraodonis;Pseudoalteromonas porphyrae;Pseudoalteromonas distincta;Pseudoalteromonas sp. S4488;Pseudoalteromonas sp. MEBiC 03607;Pseudoalteromonas sp. MQS005;Pseudoalteromonas donghaensis;Pseudoalteromonas sp. ECSMB14103;Pseudoalteromonas arabiensis;Pseudoalteromonas sp. S4389;Pseudoalteromonas sp. S185;Colwellia | |

| | | | | | |
|---|---|---|---|---|---|
| | | | | sp.;Pseudoalteromonas sp. BSi20480;Pseudoalteromonas sp. CO302Y;Pseudoalteromonas sp. H71;Pseudoalteromonas sp. SM9913;Pseudoalteromonas sp. Bsw20308 [Bacteria] | |
| 439 | WP_166839070.1 | tetratricopeptide repeat protein [Rheinheimera pleomorphica] | 22928 | [] | 1 |
| 440 | WP_026349298.1 | elongation factor P [Rheinheimera perlucida] | 21055 | Rheinheimera perlucida [Bacteria] | 1 |
| 441 | WP_097111270.1 | Glu/Leu/Phe/Val dehydrogenase [Rheinheimera tuosuensis] | 37435 | Rheinheimera tuosuensis [Bacteria] | 1 |
| 442 | WP_166837877.1 | efflux RND transporter permease subunit [Rheinheimera pleomorphica] | 114334 | [] | 1 |
| 443 | WP_070050903.1 | Tol-Pal system protein TolB [Rheinheimera salexigens] | 49288 | Rheinheimera salexigens [Bacteria] | 1 |
| 444 | WP_127023565.1 | 2-hydroxyacid dehydrogenase [Rheinheimera sp. LHK132] | 36282 | Rheinheimera sp. LHK132 [Bacteria] | 1 |
| 445 | TXH96336.1 | transcription elongation factor GreA [Rheinheimera sp.] | 17452 | Rheinheimera sp. [Bacteria] | 1 |
| 446 | WP_008218782.1 | class I SAM-dependent methyltransferase [Rheinheimera nanhaiensis] | 30051 | Rheinheimera nanhaiensis [Bacteria] | 1 |
| 447 | WP_097111411.1 | TIGR00153 family protein [Rheinheimera tuosuensis] | 25663 | Rheinheimera tuosuensis [Bacteria] | 1 |
| 448 | WP_092791626.1 | LysM peptidoglycan-binding domain-containing protein [Rheinheimera pacifica] | 59685 | Rheinheimera pacifica [Bacteria] | 1 |
| 449 | WP_068064079.1 | phosphoglycerate dehydrogenase [Rheinheimera sp. SA_1] | 44686 | Rheinheimera sp. SA_1 [Bacteria] | 1 |
| 450 | WP_068069550.1 | S9 family peptidase [Rheinheimera sp. SA_1] | 72097 | Rheinheimera sp. SA_1 [Bacteria] | 1 |
| 451 | WP_147905520.1 | diaminopimelate decarboxylase [Rheinheimera tangshanensis] | 45578 | Pararheinheimera tangshanensis [Bacteria] | 1 |
| 452 | WP_019676263.1 | 5-(carboxyamino)imidazole ribonucleotide mutase [Rheinheimera perlucida] | 16643 | Rheinheimera perlucida [Bacteria] | 1 |
| 453 | WP_127686236.1 | PrkA family serine protein kinase [Rheinheimera sp. YQF-1] | 73969 | Rheinheimera sp. YQF-1 [Bacteria] | 1 |

| | | | | | |
|---|---|---|---|---|---|
| 454 | WP_092792885.1 | polysaccharide biosynthesis tyrosine autokinase [Rheinheimera pacifica] | 79927 | Rheinheimera pacifica [Bacteria] | 1 |
| 455 | WP_019676452.1 | chemotaxis response regulator protein-glutamate methylesterase [Rheinheimera perlucida] | 41247 | Rheinheimera perlucida [Bacteria] | 1 |
| 456 | WP_019676425.1 | UDP-N-acetylglucosamine 4,6-dehydratase [Rheinheimera perlucida] | 45225 | Rheinheimera perlucida [Bacteria] | 1 |
| 457 | WP_127701180.1 | pyridoxal phosphate-dependent aminotransferase [Rheinheimera riviphila] | 45479 | Rheinheimera sp. KYPC3 [Bacteria] | 1 |
| 458 | WP_019675156.1 | ribonuclease E inhibitor RraB [Rheinheimera perlucida] | 14253 | Rheinheimera perlucida [Bacteria] | 1 |
| 459 | WP_178089506.1 | S-(hydroxymethyl)glutathione dehydrogenase/class III alcohol dehydrogenase [Rheinheimera sp. YQF-2] | 40189 | [] | 1 |
| 460 | WP_197490007.1 | TonB-dependent receptor [Rheinheimera sp. SA_1] | 112523 | [] | 1 |
| 461 | WP_027672934.1 | ABC transporter ATP-binding protein [Rheinheimera baltica] | 68169 | Rheinheimera baltica [Bacteria] | 1 |
| 462 | WP_127025361.1 | electron transfer flavoprotein subunit beta/FixA family protein [Rheinheimera sp. LHK132] | 26524 | Rheinheimera sp. LHK132 [Bacteria] | 1 |
| 463 | WP_070048168.1 | outer membrane lipid asymmetry maintenance protein MlaD [Rheinheimera salexigens] | 16326 | Rheinheimera salexigens [Bacteria] | 1 |
| 464 | WP_166839307.1 | RNase adapter RapZ [Rheinheimera pleomorphica] | 32740 | [] | 1 |
| 465 | WP_166837967.1 | H-NS histone family protein [Rheinheimera pleomorphica] | 19823 | [] | 1 |
| 466 | WP_027669589.1 | aminopeptidase P family protein [Rheinheimera baltica] | 44724 | Rheinheimera baltica [Bacteria] | 1 |
| 467 | WP_166839291.1 | Do family serine endopeptidase [Rheinheimera pleomorphica] | 47778 | [] | 1 |
| 468 | WP_097112385.1 | stringent starvation protein A [Rheinheimera tuosuensis] | 24445 | Rheinheimera tuosuensis [Bacteria] | 1 |
| 469 | WP_097110567.1 | thiol:disulfide interchange protein DsbA/DsbL [Rheinheimera tuosuensis] | 24127 | Rheinheimera tuosuensis [Bacteria] | 1 |
| 470 | WP_166836103.1 | prolyl oligopeptidase family serine peptidase [Rheinheimera pleomorphica] | 75893 | [] | 1 |

| | | | | | |
|---|---|---|---|---|---|
| 471 | WP_008220213.1 | energy-dependent translational throttle protein EttA [Rheinheimera nanhaiensis] | 62418 | Rheinheimera nanhaiensis [Bacteria] | 1 |
| 472 | WP_132586598.1 | ribonuclease P protein component [Rheinheimera sp. D18] | 14124 | Rheinheimera sp. D18 [Bacteria] | 1 |
| 473 | WP_068228110.1 | PepSY domain-containing protein [Rheinheimera sp. EpRS3] | 52986 | Rheinheimera sp. EpRS3 [Bacteria] | 1 |
| 474 | WP_173499661.1 | PD40 domain-containing protein [Rheinheimera sp. YQF-2] | 119572 | [] | 1 |
| 475 | WP_070050125.1 | outer membrane protein assembly factor BamA [Rheinheimera salexigens] | 93177 | Rheinheimera salexigens [Bacteria] | 1 |
| 476 | WP_134052449.1 | bacterioferritin [Rheinheimera aquimaris] | 18202 | Rheinheimera aquimaris [Bacteria] | 1 |
| 477 | WP_092791691.1 | DUF885 domain-containing protein [Rheinheimera pacifica] | 69593 | Rheinheimera pacifica [Bacteria] | 1 |
| 478 | WP_008900393.1 | glutamine-hydrolyzing carbamoyl-phosphate synthase small subunit [Rheinheimera sp. A13L] | 40953 | Rheinheimera sp. A13L [Bacteria] | 1 |
| 479 | WP_097111610.1 | carbon storage regulator CsrA [Rheinheimera tuosuensis] | 7225 | Rheinheimera tuosuensis [Bacteria] | 1 |
| 480 | ALZ75967.1 | phosphate ABC transporter ATP-binding protein [Rheinheimera sp. F8] | 30454 | Rheinheimera sp. F8 [Bacteria] | 1 |
| 481 | WP_132581740.1 | LemA family protein [Rheinheimera sp. D18] | 21295 | Rheinheimera sp. D18 [Bacteria] | 1 |
| 482 | WP_166839421.1 | serine--tRNA ligase [Rheinheimera pleomorphica] | 47836 | [] | 1 |
| 483 | WP_097112364.1 | RNA polymerase factor sigma-54 [Rheinheimera tuosuensis] | 56151 | Rheinheimera tuosuensis [Bacteria] | 1 |
| 484 | WP_008900176.1 | M3 family metallopeptidase [Rheinheimera sp. A13L] | 80649 | Rheinheimera sp. A13L [Bacteria] | 1 |
| 485 | WP_166840959.1 | SDR family oxidoreductase [Rheinheimera pleomorphica] | 26825 | [] | 1 |
| 486 | WP_132580743.1 | Xaa-Pro dipeptidase [Rheinheimera sp. D18] | 50401 | Rheinheimera sp. D18 [Bacteria] | 1 |
| 487 | WP_127020883.1 | insulinase family protein [Rheinheimera sp. LHK132] | 104713 | Rheinheimera sp. LHK132 [Bacteria] | 1 |
| 488 | WP_173499327.1 | 2,3-bisphosphoglycerate-independent phosphoglycerate mutase [Rheinheimera sp. YQF-2] | 56262 | [] | 1 |
| 489 | WP_132583646.1 | glutamine--tRNA ligase [Rheinheimera sp. D18] | 63921 | Rheinheimera sp. D18 [Bacteria] | 1 |

| | | | | | |
|---|---|---|---|---|---|
| 490 | ALZ74377.1 | glutamate--tRNA ligase [Rheinheimera sp. F8] | 52571 | Rheinheimera sp. F8 [Bacteria] | 1 |
| 491 | WP_027672194.1 | uroporphyrinogen decarboxylase [Rheinheimera baltica] | 40044 | Rheinheimera baltica [Bacteria] | 1 |
| 492 | WP_008218731.1 | UDP-N-acetylmuramoyl-tripeptide--D-alanyl-D-alanine ligase [Rheinheimera nanhaiensis] | 48655 | Rheinheimera nanhaiensis [Bacteria] | 1 |
| 493 | WP_008900467.1 | MULTISPECIES: shikimate kinase AroK [Chromatiaceae] | 19403 | Rheinheimera sp. A13L;Pararheinheimera mesophila;Rheinheimera sp. KL1;Pararheinheimera tangshanensis;Rheinheimera sp. YQF-1;Rheinheimera sp. LHK132 [Bacteria] | 1 |
| 494 | WP_070047955.1 | cytochrome c5 family protein [Rheinheimera salexigens] | 13790 | Rheinheimera salexigens [Bacteria] | 1 |
| 495 | WP_173501899.1 | ribosome assembly RNA-binding protein YhbY [Rheinheimera sp. YQF-2] | 11006 | [] | 1 |
| 496 | WP_008899947.1 | Do family serine endopeptidase [Rheinheimera sp. A13L] | 47445 | Rheinheimera sp. A13L [Bacteria] | 1 |
| 497 | WP_082971757.1 | DEAD/DEAH box helicase [Rheinheimera sp. SA_1] | 50116 | Rheinheimera sp. SA_1 [Bacteria] | 1 |
| 498 | WP_173500455.1 | UTP--glucose-1-phosphate uridylyltransferase GalU [Rheinheimera sp. YQF-2] | 33010 | [] | 1 |
| 499 | WP_027672817.1 | S41 family peptidase [Rheinheimera baltica] | 119854 | Rheinheimera baltica [Bacteria] | 1 |
| 500 | WP_092792953.1 | pyruvate kinase [Rheinheimera pacifica] | 51445 | Rheinheimera pacifica [Bacteria] | 1 |
| 501 | WP_147903016.1 | signal recognition particle-docking protein FtsY [Rheinheimera tangshanensis] | 41566 | Pararheinheimera tangshanensis [Bacteria] | 1 |
| 502 | WP_019674440.1 | universal stress protein UspE [Rheinheimera perlucida] | 34882 | Rheinheimera perlucida [Bacteria] | 1 |
| 503 | WP_173499276.1 | RNA polymerase-binding protein DksA [Rheinheimera sp. YQF-2] | 17326 | [] | 1 |
| 504 | WP_031565234.1 | DUF413 domain-containing protein [Pararheinheimera texasensis] | 13623 | Rheinheimera sp.;Pararheinheimera texasensis [Bacteria] | 1 |
| 505 | WP_173502073.1 | cysteine--tRNA ligase [Rheinheimera sp. YQF-2] | 51235 | [] | 1 |

| | | | | | |
|---|---|---|---|---|---|
| 506 | WP_008218374.1 | tetratricopeptide repeat protein [Rheinheimera nanhaiensis] | 47585 | Rheinheimera nanhaiensis [Bacteria] | 1 |
| 507 | WP_026349527.1 | MULTISPECIES: GbsR/MarR family transcriptional regulator [Rheinheimera] | 21145 | Rheinheimera perlucida;Rheinheimera tuosuensis [Bacteria] | 1 |
| 508 | WP_134058707.1 | DUF885 domain-containing protein [Rheinheimera aquimaris] | 66277 | Rheinheimera aquimaris [Bacteria] | 1 |
| 509 | WP_132586170.1 | BON domain-containing protein [Rheinheimera sp. D18] | 19095 | Rheinheimera sp. D18 [Bacteria] | 1 |
| 510 | WP_027670772.1 | FKBP-type peptidyl-prolyl cis-trans isomerase [Rheinheimera baltica] | 15454 | Rheinheimera baltica [Bacteria] | 1 |
| 511 | WP_068064372.1 | MULTISPECIES: acetyl-CoA carboxylase carboxyl transferase subunit alpha [Rheinheimera] | 35546 | Rheinheimera sp. KYPC3;Rheinheimera sp. SA_1 [Bacteria] | 1 |
| 512 | WP_173502545.1 | replicative DNA helicase [Rheinheimera sp. YQF-2] | 51087 | [] | 1 |
| 513 | WP_092789533.1 | histidine phosphatase family protein [Rheinheimera pacifica] | 18136 | Rheinheimera pacifica [Bacteria] | 1 |
| 514 | KOO58357.1 | chain-length determining protein [Rheinheimera sp. KL1] | 80268 | Rheinheimera sp. KL1 [Bacteria] | 1 |
| 515 | WP_068229883.1 | peptidylprolyl isomerase [Rheinheimera sp. EpRS3] | 18096 | Rheinheimera sp. EpRS3 [Bacteria] | 1 |
| 516 | WP_027670423.1 | dUTP diphosphatase [Rheinheimera baltica] | 16279 | Rheinheimera baltica [Bacteria] | 1 |
| 517 | WP_147903417.1 | branched-chain amino acid aminotransferase [Rheinheimera tangshanensis] | 35585 | Pararheinheimera tangshanensis [Bacteria] | 1 |
| 518 | WP_092791850.1 | PA2169 family four-helix-bundle protein [Rheinheimera pacifica] | 17280 | Rheinheimera pacifica [Bacteria] | 1 |
| 519 | WP_068230033.1 | maleylacetoacetate isomerase [Rheinheimera sp. EpRS3] | 24210 | Rheinheimera sp. EpRS3 [Bacteria] | 1 |
| 520 | WP_127686133.1 | acetyl-CoA carboxylase carboxyl transferase subunit alpha [Rheinheimera sp. YQF-1] | 35484 | Rheinheimera sp. YQF-1 [Bacteria] | 1 |
| 521 | ALZ74722.1 | hypothetical protein ATY27_02430 [Rheinheimera sp. F8] | 19209 | Rheinheimera sp. F8 [Bacteria] | 1 |
| 522 | WP_166839904.1 | transketolase [Rheinheimera pleomorphica] | 72285 | [] | 1 |

| | | | | | |
|---|---|---|---|---|---|
| 523 | WP_166837036.1 | PBP1A family penicillin-binding protein [Rheinheimera pleomorphica] | 96407 | [] | 1 |
| 524 | WP_192826704.1 | DUF1329 domain-containing protein [Rheinheimera sp. A13L] | 50658 | [] | 1 |
| 525 | WP_008899776.1 | MULTISPECIES: 30S ribosomal protein S21 [Rheinheimera] | 8539 | Pararheinheimera tangshanensis;Rheinheimera sp. A13L;Rheinheimera sp. YQF-1;Rheinheimera sp. F8;Rheinheimera sp. KL1 [Bacteria] | 1 |
| 526 | WP_170949057.1 | transcription termination/antitermination protein NusG [Rheinheimera tuosuensis] | 20425 | [] | 1 |
| 527 | TXH92975.1 | UDP-N-acetylmuramate:L-alanyl-gamma-D-glutamyl-meso-diaminopimelate ligase [Rheinheimera sp.] | 49028 | Rheinheimera sp. [Bacteria] | 1 |
| 528 | WP_147904908.1 | TonB-dependent receptor [Rheinheimera tangshanensis] | 100816 | Pararheinheimera tangshanensis [Bacteria] | 1 |
| 529 | WP_127700799.1 | TonB-dependent receptor [Rheinheimera riviphila] | 89917 | Rheinheimera sp. KYPC3 [Bacteria] | 1 |
| 530 | WP_092789077.1 | TonB-dependent receptor [Rheinheimera pacifica] | 108693 | Rheinheimera pacifica [Bacteria] | 1 |
| 531 | WP_127685973.1 | NADH:ubiquinone reductase (Na(+)-transporting) subunit B [Rheinheimera sp. YQF-1] | 44254 | Rheinheimera sp. YQF-1 [Bacteria] | 1 |
| 532 | KOO58461.1 | argininosuccinate lyase [Rheinheimera sp. KL1] | 50244 | [] | 1 |
| 533 | WP_068232364.1 | OmpH family outer membrane protein [Rheinheimera sp. EpRS3] | 19177 | Rheinheimera sp. EpRS3 [Bacteria] | 1 |
| 534 | WP_173499748.1 | DUF3450 family protein [Rheinheimera sp. YQF-2] | 29062 | [] | 1 |
| 535 | WP_008220004.1 | cytochrome c maturation protein CcmE [Rheinheimera nanhaiensis] | 18713 | Rheinheimera nanhaiensis [Bacteria] | 1 |
| 536 | WP_070049338.1 | pyridoxal phosphate-dependent aminotransferase [Rheinheimera salexigens] | 45481 | Rheinheimera salexigens [Bacteria] | 1 |
| 537 | WP_053424342.1 | phosphate ABC transporter ATP-binding protein [Rheinheimera tangshanensis] | 31110 | Pararheinheimera tangshanensis;Rheinheimera sp. KL1 [Bacteria] | 1 |
| 538 | WP_134055868.1 | bifunctional GNAT family N-acetyltransferase/carbon-nitrogen hydrolase family protein [Rheinheimera aquimaris] | 59010 | Rheinheimera aquimaris [Bacteria] | 1 |

| | | | | | |
|---|---|---|---|---|---|
| 539 | WP_019677070.1 | BON domain-containing protein [Rheinheimera perlucida] | 20253 | Rheinheimera perlucida [Bacteria] | 1 |
| 540 | WP_173501308.1 | thiamine pyrophosphate-dependent dehydrogenase E1 component subunit alpha [Rheinheimera sp. YQF-2] | 43907 | [] | 1 |
| 541 | WP_068235194.1 | threonine--tRNA ligase [Rheinheimera sp. EpRS3] | 72745 | Rheinheimera sp. EpRS3 [Bacteria] | 1 |
| | | | | Total (with parcimony) | 2663 |

| *Table S6: List of proteins identified for SS13 isolate using the database created from whole genome sequencing.* | | | | |
|---|---|---|---|---|
| RAWfile | ['Q14963_SS13.raw'] | | | |
| DATpath | \\susa\Data-Mascot\20230614 | | | |
| DATfile | F484206.dat | | | |
| DBs | DB_CL_SS13_genome | | | |
| DB fasta(s) | C:/inetpub/mascot/sequence/DB_CL/DB_CL_SS13_genome/current/DB_CL_SS13_genome_01.fasta | | | |
| Number_of_spectra | 14637 | | | |
| Mascot pvalue (FDR=0.0100) | 0,0199 | | | |
| Number_of_PSMs | 7487 | | | |
| | | | | |
| **Protein group** | **Accession** | **Description** | **Molecular Weight (Da)** | **Spectral count** |
| 1 | KIAPNMCA_00081 | hypothetical protein | 50227,48 | 491 |
| 2 | KIAPNMCA_04618 | 60 kDa chaperonin | 31517,6 | 147 |
| 3 | KIAPNMCA_05496 | Vitamin B12 transporter BtuB | 79246,45 | 115 |
| 4 | KIAPNMCA_02205 | Alanine dehydrogenase | 39502,65 | 85 |
| 5 | KIAPNMCA_01774 | Vitamin B12 transporter BtuB | 56587,37 | 79 |
| 6 | KIAPNMCA_00914 | Chaperone protein DnaK | 57884,82 | 72 |
| 7 | KIAPNMCA_04995 | DNA-directed RNA polymerase subunit beta' | 154956,6 | 70 |
| 8 | KIAPNMCA_04990 | Elongation factor Tu | 27350,24 | 61 |
| 9 | KIAPNMCA_04996 | DNA-directed RNA polymerase subunit beta | 150537,09 | 61 |
| 10 | KIAPNMCA_01169 | hypothetical protein | 84848,43 | 59 |
| 11 | KIAPNMCA_01391 | Pyruvate dehydrogenase E1 component | 73976,48 | 56 |
| 12 | KIAPNMCA_01836 | Succinate--CoA ligase [ADP-forming] subunit beta | 41857,8 | 51 |
| 13 | KIAPNMCA_01389 | Dihydrolipoyl dehydrogenase | 50168,19 | 50 |
| 14 | KIAPNMCA_05497 | Vitamin B12 transporter BtuB | 29437,2 | 48 |

| | | | | |
|---|---|---|---|---|
| 15 | KIAPNMCA_00024 | Elongation factor G 1 | 77159,78 | 48 |
| 16 | KIAPNMCA_00294 | DNA-binding protein HU-beta | 8509,57 | 47 |
| 17 | KIAPNMCA_05353 | ATP synthase subunit beta | 50004,75 | 46 |
| 18 | KIAPNMCA_01776 | Vitamin B12 transporter BtuB | 33703,39 | 46 |
| 18 | KIAPNMCA_00499 | Vitamin B12 transporter BtuB | 42580,66 | 5 |
| 19 | KIAPNMCA_03723 | hypothetical protein | 11553,84 | 45 |
| 20 | KIAPNMCA_02603 | Isocitrate dehydrogenase [NADP] 2 | 81217,7 | 45 |
| 21 | KIAPNMCA_05127 | hypothetical protein | 48092,95 | 43 |
| 22 | KIAPNMCA_05356 | ATP synthase subunit alpha | 39984,83 | 42 |
| 23 | KIAPNMCA_03599 | Tol-Pal system protein TolQ | 42189,38 | 41 |
| 24 | KIAPNMCA_03601 | hypothetical protein | 26440,53 | 41 |
| 25 | KIAPNMCA_00470 | hypothetical protein | 112591,92 | 40 |
| 26 | KIAPNMCA_04617 | 60 kDa chaperonin | 11562,12 | 39 |
| 27 | KIAPNMCA_01840 | hypothetical protein | 62590,63 | 39 |
| 28 | KIAPNMCA_03813 | Chaperone protein HtpG | 41159,94 | 38 |
| 29 | KIAPNMCA_02497 | NAD-specific glutamate dehydrogenase | 75445,36 | 38 |
| 30 | KIAPNMCA_01835 | Dihydrolipoyllysine-residue succinyltransferase component of 2-oxoglutarate dehydrogenase complex | 38296,98 | 38 |
| 31 | KIAPNMCA_04385 | Outer membrane porin F | 39243,77 | 37 |
| 32 | KIAPNMCA_05947 | Outer membrane protein MIP | 26319,6 | 37 |
| 33 | KIAPNMCA_04956 | DNA-directed RNA polymerase subunit alpha | 36766,38 | 37 |
| 34 | KIAPNMCA_02367 | 30S ribosomal protein S1 | 46251,88 | 36 |
| 35 | KIAPNMCA_02499 | NAD-specific glutamate dehydrogenase | 58316,64 | 36 |
| 36 | KIAPNMCA_04407 | Malate dehydrogenase | 31464,65 | 36 |
| 37 | KIAPNMCA_00299 | Trigger factor | 48406,44 | 33 |
| 38 | KIAPNMCA_04616 | 60 kDa chaperonin | 14905,49 | 32 |
| 39 | KIAPNMCA_01834 | 2-oxoglutarate dehydrogenase E1 component | 105364,82 | 32 |
| 40 | KIAPNMCA_01323 | hypothetical protein | 33912,5 | 30 |
| 41 | KIAPNMCA_04981 | 50S ribosomal protein L4 | 22157,94 | 30 |

| | | | | |
|---|---|---|---|---|
| 42 | KIAPNMCA_04415 | Aconitate hydratase B | 72443,84 | 29 |
| 43 | KIAPNMCA_00205 | Efflux pump periplasmic linker BepF | 40298,04 | 29 |
| 44 | KIAPNMCA_04878 | Dipeptidyl carboxypeptidase | 81059,38 | 29 |
| 45 | KIAPNMCA_02984 | 4-hydroxyphenylpyruvate dioxygenase | 39937,37 | 29 |
| 46 | KIAPNMCA_04124 | Modulator of FtsH protease HflC | 32914,06 | 28 |
| 47 | KIAPNMCA_04983 | 50S ribosomal protein L3 | 16625,73 | 27 |
| 48 | KIAPNMCA_04979 | 50S ribosomal protein L2 | 30208,2 | 27 |
| 49 | KIAPNMCA_05717 | Vitamin B12 transporter BtuB | 37540,12 | 26 |
| 50 | KIAPNMCA_03730 | Peptidoglycan-associated lipoprotein | 19274,89 | 26 |
| 51 | KIAPNMCA_00543 | Bifunctional protein PutA | 66471,4 | 26 |
| 52 | KIAPNMCA_00293 | Peptidyl-prolyl cis-trans isomerase D | 71042,32 | 26 |
| 53 | KIAPNMCA_00295 | Lon protease | 59402,71 | 26 |
| 54 | KIAPNMCA_04957 | 30S ribosomal protein S4 | 23379,72 | 26 |
| 55 | KIAPNMCA_05346 | hypothetical protein | 88469,38 | 25 |
| 56 | KIAPNMCA_05568 | Transcription termination factor Rho | 47291,9 | 25 |
| 57 | KIAPNMCA_03825 | Vitamin B12 transporter BtuB | 51474,44 | 24 |
| 58 | KIAPNMCA_03826 | Vitamin B12 transporter BtuB | 28464,47 | 24 |
| 59 | KIAPNMCA_03931 | Enolase | 46131,47 | 24 |
| 60 | KIAPNMCA_05359 | ATP synthase subunit delta | 19390,24 | 24 |
| 61 | KIAPNMCA_01234 | GTP-binding protein TypA/BipA | 67840,51 | 24 |
| 62 | KIAPNMCA_01390 | Dihydrolipoyllysine-residue acetyltransferase component of pyruvate dehydrogenase complex | 58836,56 | 23 |
| 63 | KIAPNMCA_01250 | Protein-export protein SecB | 18189,16 | 23 |
| 64 | KIAPNMCA_00645 | Polyribonucleotide nucleotidyltransferase | 70125,54 | 23 |
| 65 | KIAPNMCA_01593 | Periplasmic pH-dependent serine endoprotease DegQ | 47735,24 | 22 |
| 66 | KIAPNMCA_01827 | Citrate synthase | 25777,93 | 22 |
| 67 | KIAPNMCA_01136 | Acetylornithine/succinyldiaminopimelate aminotransferase | 43824,47 | 22 |
| 68 | KIAPNMCA_00660 | ATP-dependent zinc metalloprotease FtsH | 71178,3 | 21 |
| 69 | KIAPNMCA_04399 | Chromosome partition protein Smc | 72352,36 | 21 |

| | | | | |
|---|---|---|---|---|
| 69 | KIAPNMCA_01552 | hypothetical protein | 58038,29 | 11 |
| 69 | KIAPNMCA_05529 | Methyl-accepting chemotaxis protein McpP | 39331,09 | 4 |
| 69 | KIAPNMCA_00755 | IS66 family transposase ISPsy43 | 22382,3 | 2 |
| 69 | KIAPNMCA_03775 | Methyl-accepting chemotaxis protein McpB | 44471,05 | 2 |
| 70 | KIAPNMCA_02027 | hypothetical protein | 28549,83 | 21 |
| 71 | KIAPNMCA_04997 | 50S ribosomal protein L7/L12 | 12245,5 | 21 |
| 72 | KIAPNMCA_00790 | Ribose-phosphate pyrophosphokinase | 34515,13 | 20 |
| 73 | KIAPNMCA_03594 | hypothetical protein | 30864,84 | 20 |
| 74 | KIAPNMCA_04966 | 50S ribosomal protein L6 | 18955,31 | 20 |
| 75 | KIAPNMCA_04999 | 50S ribosomal protein L1 | 24568,28 | 20 |
| 76 | KIAPNMCA_04972 | 50S ribosomal protein L14 | 13460,47 | 20 |
| 77 | KIAPNMCA_04976 | 30S ribosomal protein S3 | 26364,4 | 20 |
| 78 | KIAPNMCA_01411 | Protein translocase subunit SecA | 72460,08 | 20 |
| 79 | KIAPNMCA_04619 | 10 kDa chaperonin | 10252,52 | 20 |
| 80 | KIAPNMCA_01838 | Succinate--CoA ligase [ADP-forming] subunit alpha | 13918,13 | 20 |
| 81 | KIAPNMCA_05186 | Alkyl hydroperoxide reductase C | 9881,08 | 20 |
| 82 | KIAPNMCA_03802 | Superoxide dismutase [Fe] | 21512,58 | 20 |
| 83 | KIAPNMCA_04125 | hypothetical protein | 26522,81 | 20 |
| 84 | KIAPNMCA_00162 | Vitamin B12 transporter BtuB | 46429,17 | 20 |
| 85 | KIAPNMCA_00163 | Protein oar | 109859,82 | 20 |
| 86 | KIAPNMCA_01332 | Phosphate-binding protein PstS | 18956,74 | 20 |
| 87 | KIAPNMCA_03814 | Chaperone protein HtpG | 21157,72 | 19 |
| 88 | KIAPNMCA_04991 | Elongation factor Tu 2 | 9544,87 | 18 |
| 89 | KIAPNMCA_04964 | 30S ribosomal protein S5 | 17643,34 | 18 |
| 90 | KIAPNMCA_04159 | Outer membrane protein TolC | 32814,08 | 18 |
| 91 | KIAPNMCA_02115 | Phenylalanine--tRNA ligase beta subunit | 82497,23 | 18 |
| 92 | KIAPNMCA_04993 | 30S ribosomal protein S7 | 17705,48 | 18 |
| 93 | KIAPNMCA_05669 | Phosphoenolpyruvate carboxykinase (ATP) | 58937,45 | 18 |
| 94 | KIAPNMCA_00420 | 50S ribosomal protein L19 | 13441,31 | 18 |

| | | | | |
|---|---|---|---|---|
| 95 | KIAPNMCA_03857 | Outer membrane protein assembly factor BamA | 79229,98 | 18 |
| 96 | KIAPNMCA_03853 | Chaperone protein Skp | 13806,26 | 18 |
| 97 | KIAPNMCA_02106 | Outer membrane protein assembly factor BamC | 29196,94 | 17 |
| 98 | KIAPNMCA_01085 | Nucleoside diphosphate kinase | 15628,97 | 17 |
| 99 | KIAPNMCA_03868 | 30S ribosomal protein S2 | 15370,12 | 17 |
| 100 | KIAPNMCA_02983 | Homogentisate 1,2-dioxygenase | 43576,42 | 17 |
| 101 | KIAPNMCA_03967 | Phosphoribosylformylglycinamidine synthase | 86954,69 | 17 |
| 102 | KIAPNMCA_01331 | Phosphate-binding protein PstS | 14634,62 | 17 |
| 103 | KIAPNMCA_02957 | Serine hydroxymethyltransferase | 44016,34 | 16 |
| 104 | KIAPNMCA_04112 | 50S ribosomal protein L9 | 15691,45 | 16 |
| 105 | KIAPNMCA_03863 | Ribosome-recycling factor | 20538,91 | 16 |
| 106 | KIAPNMCA_02498 | NAD-specific glutamate dehydrogenase | 48500,83 | 16 |
| 107 | KIAPNMCA_00434 | Chaperone protein ClpB | 96521,77 | 16 |
| 108 | KIAPNMCA_00153 | 4-hydroxy-tetrahydrodipicolinate synthase | 27411,31 | 16 |
| 109 | KIAPNMCA_04096 | Alanine--tRNA ligase | 80071,06 | 16 |
| 110 | KIAPNMCA_05166 | Cytochrome c4 | 22541,2 | 15 |
| 111 | KIAPNMCA_00175 | hypothetical protein | 24783,04 | 15 |
| 112 | KIAPNMCA_03715 | Osmotically-inducible protein Y | 19752,43 | 15 |
| 113 | KIAPNMCA_04969 | 50S ribosomal protein L5 | 10432,43 | 15 |
| 114 | KIAPNMCA_03866 | Elongation factor Ts | 30666,96 | 15 |
| 115 | KIAPNMCA_00913 | Chaperone protein DnaJ | 41216,67 | 15 |
| 116 | KIAPNMCA_04998 | 50S ribosomal protein L10 | 17601,37 | 15 |
| 117 | KIAPNMCA_00201 | Asp/Glu-specific dipeptidyl-peptidase | 35580,03 | 15 |
| 118 | KIAPNMCA_01831 | Succinate dehydrogenase flavoprotein subunit | 53110,64 | 15 |
| 119 | KIAPNMCA_03731 | Tol-Pal system protein TolB | 49326,46 | 15 |
| 120 | KIAPNMCA_00649 | Translation initiation factor IF-2 | 61497,98 | 15 |
| 121 | KIAPNMCA_00891 | 50S ribosomal protein L25 | 21883,53 | 15 |
| 122 | KIAPNMCA_04418 | Neutral endopeptidase | 64644,32 | 14 |
| 123 | KIAPNMCA_04929 | Cell shape-determining protein MreB | 37072,11 | 14 |

| | | | | |
|---|---|---|---|---|
| 124 | KIAPNMCA_01590 | 50S ribosomal protein L13 | 15859,47 | 14 |
| 125 | KIAPNMCA_04115 | 30S ribosomal protein S6 | 14592,45 | 14 |
| 126 | KIAPNMCA_03098 | UDP-N-acetyl-D-glucosamine 6-dehydrogenase | 46974,6 | 14 |
| 127 | KIAPNMCA_02961 | Cold shock-like protein CspLA | 7712,92 | 14 |
| 128 | KIAPNMCA_04405 | 50S ribosomal protein L21 | 11464,18 | 14 |
| 129 | KIAPNMCA_02982 | hypothetical protein | 37081,45 | 14 |
| 130 | KIAPNMCA_01872 | Ribonuclease E | 76758,81 | 14 |
| 131 | KIAPNMCA_00021 | hypothetical protein | 19097,86 | 14 |
| 132 | KIAPNMCA_03979 | GMP synthase [glutamine-hydrolyzing] | 58751,08 | 14 |
| 133 | KIAPNMCA_04729 | Oligopeptidase A | 67207,5 | 14 |
| 134 | KIAPNMCA_03932 | CTP synthase | 60192,31 | 14 |
| 135 | KIAPNMCA_02607 | Adenylosuccinate lyase | 51111,36 | 14 |
| 136 | KIAPNMCA_00500 | Vitamin B12 transporter BtuB | 59186,26 | 14 |
| 137 | KIAPNMCA_00493 | Aerobic respiration control protein ArcA | 19949,7 | 14 |
| 138 | KIAPNMCA_04971 | 50S ribosomal protein L24 | 11570,38 | 13 |
| 139 | KIAPNMCA_03875 | 2,3,4,5-tetrahydropyridine-2,6-dicarboxylate N-succinyltransferase | 27621,97 | 13 |
| 140 | KIAPNMCA_05284 | Glutamine--fructose-6-phosphate aminotransferase [isomerizing] | 53859,86 | 13 |
| 141 | KIAPNMCA_01075 | Outer membrane protein assembly factor BamB | 43152,32 | 13 |
| 142 | KIAPNMCA_05352 | ATP synthase epsilon chain | 9669,06 | 13 |
| 143 | KIAPNMCA_04962 | 50S ribosomal protein L15 | 14889,28 | 13 |
| 144 | KIAPNMCA_01828 | Citrate synthase | 22568,1 | 13 |
| 145 | KIAPNMCA_00101 | Glycine dehydrogenase (decarboxylating) | 39106,18 | 13 |
| 146 | KIAPNMCA_04624 | Biotin carboxylase | 49265,21 | 13 |
| 147 | KIAPNMCA_04992 | Elongation factor G | 77682,85 | 13 |
| 148 | KIAPNMCA_01833 | Succinate dehydrogenase iron-sulfur subunit | 27216,53 | 13 |
| 149 | KIAPNMCA_03338 | UDP-glucose 6-dehydrogenase | 49551,26 | 13 |
| 150 | KIAPNMCA_04393 | Chaperone SurA | 42746,11 | 13 |
| 151 | KIAPNMCA_02732 | Flagellin | 28700,92 | 13 |
| 151 | KIAPNMCA_02735 | Flagellin | 28554,86 | 1 |

| | | | | |
|---|---|---|---|---|
| 152 | KIAPNMCA_05360 | ATP synthase subunit b | 17415,42 | 13 |
| 153 | KIAPNMCA_03350 | hypothetical protein | 23045,79 | 13 |
| 154 | KIAPNMCA_03346 | hypothetical protein | 81599,5 | 12 |
| 155 | KIAPNMCA_05718 | Fe(3+) dicitrate transport protein FecA | 23477,41 | 12 |
| 156 | KIAPNMCA_05126 | Vitamin B12 transporter BtuB | 36694,63 | 12 |
| 157 | KIAPNMCA_00202 | Asp/Glu-specific dipeptidyl-peptidase | 23387,84 | 12 |
| 158 | KIAPNMCA_00438 | Outer membrane protein assembly factor BamD | 32746,71 | 12 |
| 159 | KIAPNMCA_01456 | Protein translocase subunit SecD | 36262,82 | 12 |
| 160 | KIAPNMCA_01125 | Phosphoribosylamine--glycine ligase | 45279,08 | 12 |
| 161 | KIAPNMCA_01286 | hypothetical protein | 43324,48 | 12 |
| 162 | KIAPNMCA_00296 | Lon protease | 31914,64 | 12 |
| 163 | KIAPNMCA_00317 | hypothetical protein | 20366,83 | 11 |
| 164 | KIAPNMCA_05895 | hypothetical protein | 100798,37 | 11 |
| 165 | KIAPNMCA_05000 | 50S ribosomal protein L11 | 14990 | 11 |
| 166 | KIAPNMCA_00034 | Dipeptidyl-peptidase 5 | 75604,99 | 11 |
| 167 | KIAPNMCA_01325 | Phosphate import ATP-binding protein PstB 3 | 32646,72 | 11 |
| 168 | KIAPNMCA_04017 | Protein GrpE | 19475,74 | 11 |
| 169 | KIAPNMCA_04122 | Adenylosuccinate synthetase | 47259,55 | 11 |
| 170 | KIAPNMCA_04978 | 30S ribosomal protein S19 | 10434,72 | 11 |
| 171 | KIAPNMCA_00652 | Transcription termination/antitermination protein NusA | 55425,27 | 11 |
| 172 | KIAPNMCA_00930 | Outer membrane protein W | 24678,73 | 11 |
| 173 | KIAPNMCA_03982 | Inosine-5'-monophosphate dehydrogenase | 24905,04 | 11 |
| 174 | KIAPNMCA_00545 | Bifunctional protein PutA | 38582,01 | 11 |
| 175 | KIAPNMCA_03602 | Fructose-bisphosphate aldolase | 38643,42 | 11 |
| 176 | KIAPNMCA_03105 | FKBP-type 22 kDa peptidyl-prolyl cis-trans isomerase | 21880,98 | 11 |
| 177 | KIAPNMCA_03597 | Biopolymer transport protein ExbD | 14931,81 | 10 |
| 178 | KIAPNMCA_01883 | Acyl carrier protein | 8664,29 | 10 |
| 179 | KIAPNMCA_00071 | hypothetical protein | 10794,52 | 10 |
| 180 | KIAPNMCA_01471 | hypothetical protein | 21545,01 | 10 |

| | | | | |
|---|---|---|---|---|
| 181 | KIAPNMCA_04967 | 30S ribosomal protein S8 | 13951,54 | 10 |
| 182 | KIAPNMCA_03349 | hypothetical protein | 18846,31 | 10 |
| 183 | KIAPNMCA_04980 | 50S ribosomal protein L23 | 10988,95 | 10 |
| 184 | KIAPNMCA_04276 | hypothetical protein | 74862,74 | 10 |
| 185 | KIAPNMCA_00663 | Lysine--tRNA ligase | 57935,36 | 10 |
| 186 | KIAPNMCA_02202 | Cold shock-like protein CspA | 7484,78 | 10 |
| 187 | KIAPNMCA_00618 | D-alanyl-D-alanine carboxypeptidase DacC | 23457,96 | 10 |
| 188 | KIAPNMCA_02375 | DNA gyrase subunit A | 87645,62 | 10 |
| 189 | KIAPNMCA_04129 | RNA-binding protein Hfq | 9556,92 | 10 |
| 190 | KIAPNMCA_00728 | Na(+)-translocating NADH-quinone reductase subunit A | 32195,95 | 10 |
| 191 | KIAPNMCA_03902 | Prolyl endopeptidase | 48238,1 | 10 |
| 192 | KIAPNMCA_01839 | Vitamin B12 transporter BtuB | 22709,77 | 10 |
| 193 | KIAPNMCA_02305 | DNA protection during starvation protein | 17287,93 | 10 |
| 194 | KIAPNMCA_04324 | Tricorn protease | 60168,04 | 10 |
| 195 | KIAPNMCA_00544 | Bifunctional protein PutA | 36633,87 | 10 |
| 196 | KIAPNMCA_03607 | S-adenosylmethionine synthase | 41999,16 | 10 |
| 197 | KIAPNMCA_03791 | 3-oxoacyl-[acyl-carrier-protein] synthase 1 | 42362,06 | 10 |
| 198 | KIAPNMCA_01236 | Glutamine synthetase | 32381,35 | 10 |
| 199 | KIAPNMCA_05915 | Dipeptidyl aminopeptidase BIII | 74432,04 | 10 |
| 200 | KIAPNMCA_00099 | Glycine dehydrogenase (decarboxylating) | 21321,42 | 9 |
| 201 | KIAPNMCA_02929 | Vitamin B12 transporter BtuB | 17760,12 | 9 |
| 202 | KIAPNMCA_02542 | Ferric uptake regulation protein | 16401,47 | 9 |
| 203 | KIAPNMCA_04965 | 50S ribosomal protein L18 | 12718,86 | 9 |
| 204 | KIAPNMCA_00658 | Phosphoglucosamine mutase | 48600,06 | 9 |
| 205 | KIAPNMCA_00082 | Secretin XpsD | 73284,45 | 9 |
| 206 | KIAPNMCA_01212 | ATP-dependent protease ATPase subunit HslU | 24674,82 | 9 |
| 207 | KIAPNMCA_00206 | Multidrug resistance protein MdtF | 49631,37 | 9 |
| 208 | KIAPNMCA_05372 | ATP-dependent RNA helicase DeaD | 28441,97 | 9 |
| 209 | KIAPNMCA_00982 | Fructose-1,6-bisphosphatase class 1 | 20933,55 | 9 |

| | | | | |
|---|---|---|---|---|
| 210 | KIAPNMCA_00154 | 4-hydroxy-tetrahydrodipicolinate synthase | 9305,73 | 9 |
| 211 | KIAPNMCA_01775 | hypothetical protein | 12575,16 | 9 |
| 212 | KIAPNMCA_04160 | Outer membrane protein TolC | 13123,96 | 9 |
| 213 | KIAPNMCA_03189 | hypothetical protein | 28089,47 | 9 |
| 214 | KIAPNMCA_05703 | Neutral endopeptidase | 66982,35 | 9 |
| 215 | KIAPNMCA_01589 | 30S ribosomal protein S9 | 14627,86 | 9 |
| 216 | KIAPNMCA_05185 | Alkyl hydroperoxide reductase C | 9441,76 | 9 |
| 217 | KIAPNMCA_02126 | hypothetical protein | 83783,18 | 9 |
| 218 | KIAPNMCA_05840 | hypothetical protein | 57284,05 | 9 |
| 219 | KIAPNMCA_02998 | Phage shock protein A | 21799,31 | 9 |
| 220 | KIAPNMCA_02415 | Tricorn protease | 84671,57 | 9 |
| 221 | KIAPNMCA_02882 | Peptidyl-prolyl cis-trans isomerase B | 18191 | 9 |
| 222 | KIAPNMCA_01308 | N-acetylcysteine deacetylase | 47096,2 | 9 |
| 223 | KIAPNMCA_04914 | Branched-chain-amino-acid transaminase 1 | 29175,81 | 9 |
| 224 | KIAPNMCA_05107 | Thiol:disulfide interchange protein DsbA | 23878,25 | 9 |
| 225 | KIAPNMCA_02672 | Chemotaxis protein CheA | 77479,48 | 9 |
| 226 | KIAPNMCA_04959 | 30S ribosomal protein S13 | 13232,39 | 9 |
| 227 | KIAPNMCA_04977 | 50S ribosomal protein L22 | 12124,76 | 8 |
| 228 | KIAPNMCA_03811 | Adenylate kinase | 23608,18 | 8 |
| 229 | KIAPNMCA_03595 | hypothetical protein | 16211,58 | 8 |
| 230 | KIAPNMCA_04682 | 50S ribosomal protein L28 | 8982,77 | 8 |
| 231 | KIAPNMCA_05893 | Protein QmcA | 29495,49 | 8 |
| 232 | KIAPNMCA_02920 | Adenine deaminase | 71381,38 | 8 |
| 233 | KIAPNMCA_02928 | Vitamin B12 transporter BtuB | 48794,88 | 8 |
| 234 | KIAPNMCA_05355 | ATP synthase gamma chain | 16690,96 | 8 |
| 235 | KIAPNMCA_04188 | RNA polymerase sigma factor RpoD | 70368,63 | 8 |
| 236 | KIAPNMCA_05357 | ATP synthase subunit alpha | 7804,2 | 8 |
| 237 | KIAPNMCA_01868 | Acetyl-coenzyme A synthetase | 44229,1 | 8 |
| 238 | KIAPNMCA_02664 | Chemotaxis protein CheW | 15481,99 | 8 |

| | | | | |
|---|---|---|---|---|
| 239 | KIAPNMCA_03867 | 30S ribosomal protein S2 | 10922,56 | 8 |
| 240 | KIAPNMCA_01324 | Phosphate-specific transport system accessory protein PhoU | 26956,88 | 8 |
| 241 | KIAPNMCA_05400 | Glycine--tRNA ligase beta subunit | 45760,36 | 8 |
| 242 | KIAPNMCA_05654 | Secretin GspD | 76568,72 | 8 |
| 243 | KIAPNMCA_00100 | Glycine dehydrogenase (decarboxylating) | 43175,45 | 8 |
| 244 | KIAPNMCA_01896 | 2-methylcitrate dehydratase (2-methyl-trans-aconitate forming) | 94822,54 | 8 |
| 244 | KIAPNMCA_01060 | Aconitate hydratase A | 98972,36 | 1 |
| 245 | KIAPNMCA_01211 | ATP-dependent protease ATPase subunit HslU | 24534,88 | 8 |
| 246 | KIAPNMCA_01210 | ATP-dependent protease subunit HslV | 18617,74 | 8 |
| 247 | KIAPNMCA_00983 | Inorganic pyrophosphatase | 19564,96 | 8 |
| 248 | KIAPNMCA_05572 | Superoxide dismutase [Cu-Zn] | 18430,94 | 8 |
| 249 | KIAPNMCA_05373 | ATP-dependent RNA helicase DeaD | 32467,05 | 8 |
| 250 | KIAPNMCA_00053 | hypothetical protein | 68866,12 | 8 |
| 251 | KIAPNMCA_01458 | Sec translocon accessory complex subunit YajC | 11237,12 | 8 |
| 252 | KIAPNMCA_04792 | Dipeptidyl-peptidase 5 | 75750,78 | 8 |
| 253 | KIAPNMCA_02414 | Tricorn protease | 38181,59 | 8 |
| 254 | KIAPNMCA_00907 | Carbamoyl-phosphate synthase large chain | 118542,99 | 8 |
| 255 | KIAPNMCA_01164 | L-arabonate dehydratase | 40766,46 | 8 |
| 256 | KIAPNMCA_00638 | hypothetical protein | 13392,94 | 8 |
| 257 | KIAPNMCA_03039 | hypothetical protein | 8642,48 | 7 |
| 258 | KIAPNMCA_02402 | Phosphoenolpyruvate synthase | 87311,98 | 7 |
| 259 | KIAPNMCA_05157 | hypothetical protein | 26636,38 | 7 |
| 260 | KIAPNMCA_03816 | Nucleoid-associated protein YbaB | 13291,67 | 7 |
| 261 | KIAPNMCA_04634 | Bifunctional purine biosynthesis protein PurH | 56659,99 | 7 |
| 262 | KIAPNMCA_05358 | ATP synthase subunit alpha | 7686,83 | 7 |
| 263 | KIAPNMCA_04144 | hypothetical protein | 63634,64 | 7 |
| 264 | KIAPNMCA_05137 | Alpha,alpha-trehalose phosphorylase | 55283,63 | 7 |
| 265 | KIAPNMCA_04963 | 50S ribosomal protein L30 | 6660,69 | 7 |
| 266 | KIAPNMCA_00131 | Septum site-determining protein MinD | 29549,47 | 7 |

| | | | | |
|---|---|---|---|---|
| 267 | KIAPNMCA_01149 | Alpha-ketoglutaric semialdehyde dehydrogenase | 54789,81 | 7 |
| 268 | KIAPNMCA_03190 | Beta-barrel assembly-enhancing protease | 34414,81 | 7 |
| 269 | KIAPNMCA_03824 | hypothetical protein | 13168,49 | 7 |
| 270 | KIAPNMCA_05498 | Phosphoenolpyruvate carboxylase | 62866,82 | 7 |
| 271 | KIAPNMCA_01895 | 2-methylcitrate synthase | 31879,41 | 7 |
| 272 | KIAPNMCA_02377 | Ribonucleoside-diphosphate reductase 1 subunit alpha | 86088,9 | 7 |
| 273 | KIAPNMCA_00729 | Enoyl-[acyl-carrier-protein] reductase [NADH] | 43630,25 | 7 |
| 274 | KIAPNMCA_04970 | 50S ribosomal protein L5 | 6051,29 | 7 |
| 275 | KIAPNMCA_01093 | Cysteine desulfurase IscS | 44765,99 | 7 |
| 276 | KIAPNMCA_02339 | hypothetical protein | 99829,49 | 7 |
| 277 | KIAPNMCA_02518 | Lipoamide acyltransferase component of branched-chain alpha-keto acid dehydrogenase complex | 30154,76 | 7 |
| 278 | KIAPNMCA_01516 | hypothetical protein | 50613,42 | 7 |
| 279 | KIAPNMCA_02907 | Aspartate aminotransferase | 19932,08 | 7 |
| 280 | KIAPNMCA_02466 | Tyrocidine synthase 3 | 335925 | 7 |
| 281 | KIAPNMCA_03734 | Tol-Pal system protein TolQ | 24888,16 | 7 |
| 282 | KIAPNMCA_03980 | Inosine-5'-monophosphate dehydrogenase | 9840,99 | 7 |
| 283 | KIAPNMCA_05388 | Membrane protein insertase YidC | 28018,08 | 7 |
| 284 | KIAPNMCA_03606 | Transketolase 1 | 55454,18 | 7 |
| 285 | KIAPNMCA_05569 | Thioredoxin 1 | 11935,19 | 7 |
| 286 | KIAPNMCA_02572 | Cysteine synthase A | 27889,72 | 7 |
| 287 | KIAPNMCA_01507 | hypothetical protein | 26899,88 | 7 |
| 288 | KIAPNMCA_01851 | Elongation factor P | 20891,35 | 6 |
| 289 | KIAPNMCA_03611 | Glutathione synthetase | 35843,5 | 6 |
| 290 | KIAPNMCA_03256 | Acyl-CoA dehydrogenase | 42448,14 | 6 |
| 291 | KIAPNMCA_00764 | Vitamin B12 transporter BtuB | 89376,9 | 6 |
| 292 | KIAPNMCA_01205 | NADP-dependent malic enzyme | 45472,61 | 6 |
| 293 | KIAPNMCA_04118 | Ribonuclease R | 66088,16 | 6 |
| 294 | KIAPNMCA_05769 | Peroxiredoxin OsmC | 15701,76 | 6 |

| | | | | |
|---|---|---|---|---|
| 295 | KIAPNMCA_02548 | hypothetical protein | 83014,92 | 6 |
| 296 | KIAPNMCA_00036 | Fumarate hydratase class I, anaerobic | 55145,2 | 6 |
| 297 | KIAPNMCA_03789 | Aspartate-semialdehyde dehydrogenase 2 | 36995,86 | 6 |
| 298 | KIAPNMCA_03261 | Acyl-CoA dehydrogenase | 42817,52 | 6 |
| 299 | KIAPNMCA_01361 | Vitamin B12 transporter BtuB | 79861,73 | 6 |
| 300 | KIAPNMCA_04982 | 50S ribosomal protein L3 | 5017,82 | 6 |
| 301 | KIAPNMCA_00103 | Aminomethyltransferase | 15982,39 | 6 |
| 302 | KIAPNMCA_01595 | UDP-N-acetylglucosamine 1-carboxyvinyltransferase | 44719,4 | 6 |
| 303 | KIAPNMCA_02274 | hypothetical protein | 42945,9 | 6 |
| 304 | KIAPNMCA_02273 | Glyceraldehyde-3-phosphate dehydrogenase-like protein | 39515,61 | 6 |
| 305 | KIAPNMCA_05873 | hypothetical protein | 27973,64 | 6 |
| 306 | KIAPNMCA_02989 | Putative pterin-4-alpha-carbinolamine dehydratase | 13003,6 | 6 |
| 307 | KIAPNMCA_01134 | N-succinylglutamate 5-semialdehyde dehydrogenase | 52862,08 | 6 |
| 308 | KIAPNMCA_01870 | hypothetical protein | 26072,2 | 6 |
| 309 | KIAPNMCA_00208 | Multidrug resistance protein MexB | 36612,39 | 6 |
| 310 | KIAPNMCA_03993 | hypothetical protein | 21653,16 | 6 |
| 311 | KIAPNMCA_00651 | Translation initiation factor IF-2 | 11634,23 | 6 |
| 312 | KIAPNMCA_00150 | Alpha-ketoglutaric semialdehyde dehydrogenase 2 | 51155,9 | 6 |
| 313 | KIAPNMCA_03274 | putative succinyl-CoA:3-ketoacid coenzyme A transferase subunit A | 17092,88 | 6 |
| 314 | KIAPNMCA_01415 | Cell division protein FtsZ | 40009,67 | 6 |
| 315 | KIAPNMCA_02587 | hypothetical protein | 13304,03 | 6 |
| 316 | KIAPNMCA_05570 | ATP-dependent RNA helicase RhlB | 47669,5 | 6 |
| 317 | KIAPNMCA_02908 | Aspartate aminotransferase | 14288,33 | 6 |
| 318 | KIAPNMCA_00604 | Leucine--tRNA ligase | 51413,05 | 6 |
| 319 | KIAPNMCA_01841 | Glutamine--tRNA ligase | 64000,66 | 6 |
| 320 | KIAPNMCA_02637 | UDP-N-acetylglucosamine 2-epimerase | 42012,4 | 6 |
| 321 | KIAPNMCA_03360 | 50S ribosomal protein L20 | 13538,55 | 6 |
| 322 | KIAPNMCA_02593 | Thioredoxin reductase | 34213,1 | 6 |

| | | | | |
|---|---|---|---|---|
| 323 | KIAPNMCA_04515 | hypothetical protein | 14625,35 | 6 |
| 324 | KIAPNMCA_04279 | Cytosol non-specific dipeptidase | 43628,16 | 6 |
| 325 | KIAPNMCA_04955 | 50S ribosomal protein L17 | 15224,03 | 6 |
| 326 | KIAPNMCA_00915 | Chaperone protein DnaK | 5644,95 | 6 |
| 327 | KIAPNMCA_01333 | Glyceraldehyde-3-phosphate dehydrogenase 3 | 33866,55 | 6 |
| 328 | KIAPNMCA_01330 | hypothetical protein | 55002,84 | 6 |
| 329 | KIAPNMCA_05433 | Peptide deformylase | 21667,41 | 5 |
| 330 | KIAPNMCA_03596 | hypothetical protein | 22430,75 | 5 |
| 331 | KIAPNMCA_03598 | Tol-Pal system protein TolQ | 19745,4 | 5 |
| 332 | KIAPNMCA_04487 | Cytosol aminopeptidase | 47143,13 | 5 |
| 333 | KIAPNMCA_02921 | hypothetical protein | 34764,81 | 5 |
| 334 | KIAPNMCA_05599 | hypothetical protein | 48696,69 | 5 |
| 335 | KIAPNMCA_02952 | 6,7-dimethyl-8-ribityllumazine synthase | 16150,57 | 5 |
| 336 | KIAPNMCA_00987 | hypothetical protein | 27552,82 | 5 |
| 337 | KIAPNMCA_03161 | hypothetical protein | 61761,67 | 5 |
| 338 | KIAPNMCA_01867 | Acetyl-coenzyme A synthetase | 19835,11 | 5 |
| 339 | KIAPNMCA_00459 | Dipeptidyl-peptidase 5 | 66034,94 | 5 |
| 340 | KIAPNMCA_03099 | UDP-N-acetylglucosamine 4-epimerase | 36122,17 | 5 |
| 341 | KIAPNMCA_05002 | Transcription termination/antitermination protein NusG | 7993,07 | 5 |
| 342 | KIAPNMCA_03864 | Uridylate kinase | 7968,1 | 5 |
| 343 | KIAPNMCA_01495 | 2-amino-3-ketobutyrate coenzyme A ligase | 14696,89 | 5 |
| 344 | KIAPNMCA_03347 | Polysialic acid transport protein KpsD | 19045,92 | 5 |
| 345 | KIAPNMCA_02433 | Glucose-1-phosphate adenylyltransferase | 45932,35 | 5 |
| 346 | KIAPNMCA_02435 | Protease 4 | 68261,38 | 5 |
| 347 | KIAPNMCA_04661 | hypothetical protein | 40691,97 | 5 |
| 348 | KIAPNMCA_04984 | 30S ribosomal protein S10 | 5184,84 | 5 |
| 349 | KIAPNMCA_04151 | DNA topoisomerase 4 subunit A | 46132,74 | 5 |
| 350 | KIAPNMCA_01894 | 2-methylisocitrate lyase | 32000,16 | 5 |
| 351 | KIAPNMCA_04939 | hypothetical protein | 19758,22 | 5 |

| | | | | |
|---|---|---|---|---|
| 352 | KIAPNMCA_00104 | Aminomethyltransferase | 23697,88 | 5 |
| 353 | KIAPNMCA_05347 | hypothetical protein | 33196,72 | 5 |
| 354 | KIAPNMCA_04123 | hypothetical protein | 44371,15 | 5 |
| 355 | KIAPNMCA_04126 | Modulator of FtsH protease HflK | 12631,45 | 5 |
| 356 | KIAPNMCA_04973 | 30S ribosomal protein S17 | 9610,03 | 5 |
| 357 | KIAPNMCA_04974 | 50S ribosomal protein L29 | 7085,94 | 5 |
| 358 | KIAPNMCA_00159 | Vitamin B12 transporter BtuB | 21075,4 | 5 |
| 359 | KIAPNMCA_01417 | Cell division protein FtsA | 32193,32 | 5 |
| 360 | KIAPNMCA_03740 | Aspartate--tRNA ligase | 65851,37 | 5 |
| 361 | KIAPNMCA_00939 | Glutamate-1-semialdehyde 2,1-aminomutase | 34144,38 | 5 |
| 362 | KIAPNMCA_03181 | hypothetical protein | 52344,36 | 5 |
| 363 | KIAPNMCA_03259 | Acetyl-CoA acetyltransferase | 40459,72 | 5 |
| 364 | KIAPNMCA_02017 | hypothetical protein | 72622,68 | 5 |
| 365 | KIAPNMCA_02304 | putative ABC transporter ATP-binding protein YbiT | 58393,73 | 5 |
| 366 | KIAPNMCA_01190 | Penicillin-binding protein 1A | 50726,73 | 5 |
| 367 | KIAPNMCA_02522 | Pyruvate dehydrogenase E1 component subunit alpha | 39397,61 | 5 |
| 368 | KIAPNMCA_01017 | CRISPR-associated protein Csy3 | 36427,68 | 5 |
| 369 | KIAPNMCA_05141 | Single-stranded DNA-binding protein | 22322,94 | 5 |
| 370 | KIAPNMCA_00423 | 30S ribosomal protein S16 | 9296,04 | 5 |
| 371 | KIAPNMCA_02436 | hypothetical protein | 32456,18 | 5 |
| 372 | KIAPNMCA_04490 | Valine--tRNA ligase | 108090,41 | 5 |
| 373 | KIAPNMCA_00492 | Regulator of RpoS | 9522,87 | 5 |
| 374 | KIAPNMCA_00893 | Ribosome-binding ATPase YchF | 39852,43 | 5 |
| 375 | KIAPNMCA_04958 | 30S ribosomal protein S11 | 13865,27 | 5 |
| 376 | KIAPNMCA_03992 | Imidazolonepropionase | 18985,03 | 5 |
| 377 | KIAPNMCA_03999 | Tyrosine--tRNA ligase | 44747,12 | 5 |
| 378 | KIAPNMCA_01586 | Ammonia monooxygenase gamma subunit | 27543,05 | 4 |
| 379 | KIAPNMCA_01150 | Acetylornithine/succinyldiaminopimelate aminotransferase | 78822,52 | 4 |
| 380 | KIAPNMCA_04029 | Efflux pump periplasmic linker BepF | 39079,22 | 4 |

| | | | | |
|---|---|---|---|---|
| 381 | KIAPNMCA_02918 | hypothetical protein | 22881,73 | 4 |
| 382 | KIAPNMCA_00257 | hypothetical protein | 12947,88 | 4 |
| 383 | KIAPNMCA_00910 | Alcohol dehydrogenase YqhD | 42246,92 | 4 |
| 384 | KIAPNMCA_00916 | Chaperone protein DnaK | 6221,14 | 4 |
| 385 | KIAPNMCA_03040 | hypothetical protein | 10140 | 4 |
| 386 | KIAPNMCA_00222 | Twitching mobility protein | 38777,24 | 4 |
| 387 | KIAPNMCA_02535 | Phosphoglucomutase | 59953,75 | 4 |
| 388 | KIAPNMCA_02427 | Asparagine--tRNA ligase | 24860,42 | 4 |
| 389 | KIAPNMCA_00092 | Type II secretion system protein G | 14805,54 | 4 |
| 390 | KIAPNMCA_01270 | Formyltetrahydrofolate deformylase | 18132,56 | 4 |
| 391 | KIAPNMCA_01074 | GTPase Der | 17191,91 | 4 |
| 392 | KIAPNMCA_03258 | Methylmalonate-semialdehyde dehydrogenase [acylating] | 31816,38 | 4 |
| 393 | KIAPNMCA_02211 | Serine--tRNA ligase | 51636,9 | 4 |
| 394 | KIAPNMCA_00116 | Glutamate--tRNA ligase | 37698,17 | 4 |
| 395 | KIAPNMCA_03603 | Phosphoglycerate kinase | 38981,4 | 4 |
| 396 | KIAPNMCA_01193 | Argininosuccinate synthase | 26972,83 | 4 |
| 397 | KIAPNMCA_05596 | Ubiquinone/menaquinone biosynthesis C-methyltransferase UbiE | 27852,41 | 4 |
| 398 | KIAPNMCA_02368 | 30S ribosomal protein S1 | 14239,71 | 4 |
| 399 | KIAPNMCA_03168 | hypothetical protein | 44804,08 | 4 |
| 400 | KIAPNMCA_02174 | Cbb3-type cytochrome c oxidase subunit CcoP2 | 36411,07 | 4 |
| 401 | KIAPNMCA_03095 | hypothetical protein | 101603,62 | 4 |
| 402 | KIAPNMCA_00132 | Cell division topological specificity factor | 10098,33 | 4 |
| 403 | KIAPNMCA_05001 | Transcription termination/antitermination protein NusG | 12456,38 | 4 |
| 404 | KIAPNMCA_02108 | Phosphoribosylaminoimidazole-succinocarboxamide synthase | 17007,87 | 4 |
| 405 | KIAPNMCA_02107 | Outer membrane protein assembly factor BamC | 11001,73 | 4 |
| 406 | KIAPNMCA_03785 | Acetyl-coenzyme A carboxylase carboxyl transferase subunit beta | 33444,01 | 4 |
| 407 | KIAPNMCA_00149 | putative peptidase | 42448,35 | 4 |
| 408 | KIAPNMCA_03276 | putative succinyl-CoA:3-ketoacid coenzyme A transferase subunit B | 9690,09 | 4 |

| | | | | |
|---|---|---|---|---|
| 409 | KIAPNMCA_02504 | putative protein | 36701,68 | 4 |
| 410 | KIAPNMCA_05169 | hypothetical protein | 13837,91 | 4 |
| 411 | KIAPNMCA_05160 | Imidazolonepropionase | 42264,51 | 4 |
| 412 | KIAPNMCA_03949 | Elongation factor 4 | 67419,13 | 4 |
| 413 | KIAPNMCA_01592 | Inner membrane protein YhcB | 16178,29 | 4 |
| 414 | KIAPNMCA_01598 | Intermembrane phospholipid transport system binding protein MlaC | 25267,23 | 4 |
| 415 | KIAPNMCA_02930 | hypothetical protein | 18215,55 | 4 |
| 416 | KIAPNMCA_00068 | hypothetical protein | 80142,05 | 4 |
| 417 | KIAPNMCA_00720 | Na(+)-translocating NADH-quinone reductase subunit F | 21954,23 | 4 |
| 418 | KIAPNMCA_05722 | Glutathione hydrolase-like YwrD proenzyme | 62160,42 | 4 |
| 419 | KIAPNMCA_02113 | Phenylalanine--tRNA ligase alpha subunit | 13265,12 | 4 |
| 420 | KIAPNMCA_00650 | hypothetical protein | 25702,5 | 4 |
| 421 | KIAPNMCA_04059 | Nitrogen regulatory protein P-II | 10981,67 | 4 |
| 422 | KIAPNMCA_01496 | 2-amino-3-ketobutyrate coenzyme A ligase | 30620,21 | 4 |
| 423 | KIAPNMCA_05935 | Biosynthetic arginine decarboxylase | 23106,91 | 4 |
| 424 | KIAPNMCA_01412 | Protein translocase subunit SecA | 30866,76 | 4 |
| 425 | KIAPNMCA_04752 | Vitamin B12 transporter BtuB | 86064,98 | 4 |
| 426 | KIAPNMCA_01832 | Succinate dehydrogenase flavoprotein subunit | 12988,38 | 4 |
| 427 | KIAPNMCA_01884 | 3-oxoacyl-[acyl-carrier-protein] synthase 2 | 46506,64 | 4 |
| 428 | KIAPNMCA_05663 | Type II secretion system protein L | 44589,08 | 4 |
| 429 | KIAPNMCA_00113 | D-3-phosphoglycerate dehydrogenase | 38682,2 | 4 |
| 430 | KIAPNMCA_01584 | Stringent starvation protein A | 14223,26 | 4 |
| 431 | KIAPNMCA_00501 | hypothetical protein | 7264,74 | 4 |
| 432 | KIAPNMCA_02906 | Aspartate aminotransferase | 9565,01 | 4 |
| 433 | KIAPNMCA_01227 | Urocanate hydratase | 57028,85 | 4 |
| 434 | KIAPNMCA_04045 | Organic hydroperoxide resistance protein OhrB | 14248,28 | 4 |
| 435 | KIAPNMCA_02990 | Phenylalanine-4-hydroxylase | 30655,53 | 4 |
| 436 | KIAPNMCA_04396 | Ribosomal RNA small subunit methyltransferase A | 30712,19 | 4 |
| 437 | KIAPNMCA_00300 | Gamma-glutamyl phosphate reductase | 45094,02 | 4 |

| | | | | |
|---|---|---|---|---|
| 438 | KIAPNMCA_01877 | 50S ribosomal protein L32 | 6189,19 | 4 |
| 439 | KIAPNMCA_01261 | hypothetical protein | 19813,58 | 4 |
| 440 | KIAPNMCA_04692 | hypothetical protein | 32834,77 | 4 |
| 441 | KIAPNMCA_05929 | Chaperedoxin | 31711,56 | 4 |
| 442 | KIAPNMCA_05957 | hypothetical protein | 18017,6 | 4 |
| 443 | KIAPNMCA_03075 | Asparagine synthetase [glutamine-hydrolyzing] 1 | 52429,64 | 4 |
| 444 | KIAPNMCA_03071 | hypothetical protein | 42959,47 | 4 |
| 445 | KIAPNMCA_05396 | DNA gyrase subunit B | 46222,5 | 4 |
| 446 | KIAPNMCA_00297 | ATP-dependent Clp protease ATP-binding subunit ClpX | 47281,57 | 4 |
| 447 | KIAPNMCA_05470 | Diaminopimelate decarboxylase | 45914,83 | 4 |
| 448 | KIAPNMCA_00261 | Toluene efflux pump membrane transporter TtgE | 21677,33 | 4 |
| 449 | KIAPNMCA_03217 | Polyketide synthase PksN | 100097,57 | 4 |
| 450 | KIAPNMCA_05692 | Vitamin B12 transporter BtuB | 102090,43 | 4 |
| 451 | KIAPNMCA_02216 | Modulator of FtsH protease YccA | 23676,52 | 3 |
| 452 | KIAPNMCA_03135 | hypothetical protein | 40243,05 | 3 |
| 453 | KIAPNMCA_00255 | hypothetical protein | 42124,04 | 3 |
| 454 | KIAPNMCA_04417 | Neutral endopeptidase | 13417,87 | 3 |
| 455 | KIAPNMCA_03729 | Cell division coordinator CpoB | 29135,95 | 3 |
| 456 | KIAPNMCA_02539 | Flavodoxin | 20049,65 | 3 |
| 457 | KIAPNMCA_04681 | 50S ribosomal protein L33 | 6056,35 | 3 |
| 458 | KIAPNMCA_00676 | Transaldolase B | 34422,83 | 3 |
| 459 | KIAPNMCA_01073 | GTPase Der | 28370,01 | 3 |
| 460 | KIAPNMCA_03103 | D-lactate dehydrogenase | 36526,93 | 3 |
| 461 | KIAPNMCA_02366 | Integration host factor subunit beta | 10682,68 | 3 |
| 462 | KIAPNMCA_03948 | Signal peptidase I | 34614,82 | 3 |
| 463 | KIAPNMCA_02084 | hypothetical protein | 34352,17 | 3 |
| 464 | KIAPNMCA_03765 | hypothetical protein | 63832,77 | 3 |
| 465 | KIAPNMCA_03162 | hypothetical protein | 22614,69 | 3 |
| 466 | KIAPNMCA_05135 | Beta-phosphoglucomutase | 17200 | 3 |

| | | | | |
|---|---|---|---|---|
| 467 | KIAPNMCA_00964 | 3-methyl-2-oxobutanoate hydroxymethyltransferase | 28152,52 | 3 |
| 468 | KIAPNMCA_03713 | hypothetical protein | 20134,01 | 3 |
| 469 | KIAPNMCA_00325 | Proline--tRNA ligase | 63839,82 | 3 |
| 470 | KIAPNMCA_02331 | Methionine--tRNA ligase | 41301,27 | 3 |
| 471 | KIAPNMCA_03780 | Amidophosphoribosyltransferase | 38092,63 | 3 |
| 472 | KIAPNMCA_00623 | Lipoyl synthase | 37170,08 | 3 |
| 473 | KIAPNMCA_03262 | Methylmalonyl-CoA carboxyltransferase 12S subunit | 58365,66 | 3 |
| 474 | KIAPNMCA_04304 | hypothetical protein | 34503,85 | 3 |
| 475 | KIAPNMCA_00603 | Leucine--tRNA ligase | 22392,3 | 3 |
| 476 | KIAPNMCA_00152 | 4-hydroxyproline 2-epimerase | 16043 | 3 |
| 477 | KIAPNMCA_03128 | hypothetical protein | 34786,36 | 3 |
| 478 | KIAPNMCA_02430 | Putative NAD(P)H nitroreductase YdjA | 20469,4 | 3 |
| 479 | KIAPNMCA_04400 | hypothetical protein | 13442,87 | 3 |
| 480 | KIAPNMCA_03580 | hypothetical protein | 17099,63 | 3 |
| 481 | KIAPNMCA_02610 | Leucine dehydrogenase | 37467,05 | 3 |
| 482 | KIAPNMCA_04938 | hypothetical protein | 17324,23 | 3 |
| 483 | KIAPNMCA_00061 | Fatty acid metabolism regulator protein | 27050,18 | 3 |
| 484 | KIAPNMCA_01189 | Penicillin-binding protein 1A | 21906,73 | 3 |
| 485 | KIAPNMCA_02374 | DNA gyrase subunit A | 10544,3 | 3 |
| 486 | KIAPNMCA_02373 | Phosphoserine aminotransferase | 26722,47 | 3 |
| 487 | KIAPNMCA_02272 | Glyceraldehyde-3-phosphate dehydrogenase-like protein | 11903,44 | 3 |
| 488 | KIAPNMCA_00412 | hypothetical protein | 42618,64 | 3 |
| 489 | KIAPNMCA_03232 | Glutamate-1-semialdehyde 2,1-aminomutase | 180066,91 | 3 |
| 490 | KIAPNMCA_01693 | hypothetical protein | 41825,54 | 3 |
| 491 | KIAPNMCA_00581 | Lon protease | 69349,86 | 3 |
| 492 | KIAPNMCA_01879 | 3-oxoacyl-[acyl-carrier-protein] synthase 3 protein 1 | 34911,58 | 3 |
| 493 | KIAPNMCA_02182 | Glyceraldehyde-3-phosphate dehydrogenase 1 | 20994,54 | 3 |
| 494 | KIAPNMCA_03705 | hypothetical protein | 62732,07 | 3 |
| 495 | KIAPNMCA_03996 | Imidazolonepropionase | 18600,76 | 3 |

| | | | | |
|---|---|---|---|---|
| 496 | KIAPNMCA_03997 | hypothetical protein | 109569,97 | 3 |
| 497 | KIAPNMCA_00207 | Multidrug resistance protein MdtC | 29380,21 | 3 |
| 498 | KIAPNMCA_04975 | 50S ribosomal protein L16 | 15547,39 | 3 |
| 499 | KIAPNMCA_00029 | S-(hydroxymethyl)glutathione dehydrogenase | 40231,39 | 3 |
| 500 | KIAPNMCA_02114 | Phenylalanine--tRNA ligase alpha subunit | 21674,92 | 3 |
| 501 | KIAPNMCA_03968 | Phosphoribosylformylglycinamidine synthase | 15856,93 | 3 |
| 502 | KIAPNMCA_01252 | putative protein YibN | 15587,35 | 3 |
| 503 | KIAPNMCA_05345 | hypothetical protein | 21266,94 | 3 |
| 504 | KIAPNMCA_00151 | putative protein | 18196,06 | 3 |
| 505 | KIAPNMCA_05934 | Biosynthetic arginine decarboxylase | 49135,85 | 3 |
| 506 | KIAPNMCA_00958 | Peptidase B | 45577,95 | 3 |
| 507 | KIAPNMCA_02341 | Isoleucine--tRNA ligase | 119185,49 | 3 |
| 508 | KIAPNMCA_01837 | Succinate--CoA ligase [ADP-forming] subunit alpha | 16752,66 | 3 |
| 509 | KIAPNMCA_00838 | hypothetical protein | 89837,73 | 3 |
| 510 | KIAPNMCA_00938 | Aspartate carbamoyltransferase catalytic subunit | 28559,43 | 3 |
| 511 | KIAPNMCA_03187 | UDP-N-acetyl-D-mannosamine dehydrogenase | 32740,45 | 3 |
| 512 | KIAPNMCA_02514 | RNA chaperone ProQ | 24734,22 | 3 |
| 513 | KIAPNMCA_00839 | hypothetical protein | 11037,41 | 3 |
| 514 | KIAPNMCA_01435 | Ribosomal RNA small subunit methyltransferase H | 11611,19 | 3 |
| 515 | KIAPNMCA_03838 | Acetyl-coenzyme A carboxylase carboxyl transferase subunit alpha | 17093,92 | 3 |
| 516 | KIAPNMCA_03833 | hypothetical protein | 32004,86 | 3 |
| 517 | KIAPNMCA_00503 | Isoaspartyl dipeptidase | 41273,75 | 3 |
| 518 | KIAPNMCA_03257 | Methylmalonate-semialdehyde dehydrogenase [acylating] | 22793,51 | 3 |
| 519 | KIAPNMCA_01426 | UDP-N-acetylmuramoyl-tripeptide--D-alanyl-D-alanine ligase | 16502,58 | 3 |
| 520 | KIAPNMCA_05026 | hypothetical protein | 53133,46 | 3 |
| 521 | KIAPNMCA_01588 | Ubiquinol-cytochrome c reductase iron-sulfur subunit | 21536,86 | 3 |
| 522 | KIAPNMCA_03483 | hypothetical protein | 50167,01 | 3 |
| 523 | KIAPNMCA_01228 | Histidine ammonia-lyase | 53978,96 | 3 |
| 524 | KIAPNMCA_04325 | Tricorn protease | 36250,08 | 3 |

| | | | | |
|---|---|---|---|---|
| 525 | KIAPNMCA_01123 | hypothetical protein | 19550,16 | 3 |
| 526 | KIAPNMCA_02923 | hypothetical protein | 112151,79 | 3 |
| 527 | KIAPNMCA_03981 | Inosine-5'-monophosphate dehydrogenase | 17752,78 | 3 |
| 528 | KIAPNMCA_03801 | Glutaredoxin 4 | 13058,57 | 3 |
| 529 | KIAPNMCA_03605 | Transketolase 1 | 16550,54 | 3 |
| 530 | KIAPNMCA_05419 | Xaa-Pro dipeptidase | 33590,19 | 3 |
| 531 | KIAPNMCA_03909 | Protein RecA | 32259,93 | 3 |
| 532 | KIAPNMCA_04089 | 30S ribosomal protein S20 | 9857,58 | 3 |
| 533 | KIAPNMCA_01069 | Inducible lysine decarboxylase | 10231,29 | 3 |
| 534 | KIAPNMCA_00723 | Na(+)-translocating NADH-quinone reductase subunit C | 19506,09 | 3 |
| 535 | KIAPNMCA_01168 | hypothetical protein | 6903,46 | 3 |
| 536 | KIAPNMCA_01163 | L-arabonate dehydratase | 25128,69 | 3 |
| 537 | KIAPNMCA_05952 | Soluble pyridine nucleotide transhydrogenase | 28749,53 | 3 |
| 538 | KIAPNMCA_02280 | 3-hydroxydecanoyl-[acyl-carrier-protein] dehydratase | 18764,55 | 3 |
| 539 | KIAPNMCA_04733 | Glutathione reductase | 29191,81 | 3 |
| 540 | KIAPNMCA_03083 | UDP-N-acetylbacillosamine transaminase | 42499,32 | 3 |
| 541 | KIAPNMCA_03089 | UDP-N-acetyl-2-amino-2-deoxy-D-glucuronate oxidase | 16549,38 | 3 |
| 542 | KIAPNMCA_00680 | D-galactonate dehydratase | 44057,08 | 3 |
| 543 | KIAPNMCA_01235 | Glutamine synthetase | 21967,63 | 3 |
| 544 | KIAPNMCA_04097 | Alanine--tRNA ligase | 12147,93 | 3 |
| 545 | KIAPNMCA_05394 | Beta sliding clamp | 28400,77 | 2 |
| 546 | KIAPNMCA_01070 | Biodegradative arginine decarboxylase | 54816,23 | 2 |
| 547 | KIAPNMCA_01587 | Cytochrome b | 32712,36 | 2 |
| 548 | KIAPNMCA_03337 | UDP-glucose 4-epimerase | 35280,89 | 2 |
| 549 | KIAPNMCA_02401 | Sodium/proton-dependent alanine carrier protein | 53915,15 | 2 |
| 550 | KIAPNMCA_05393 | Beta sliding clamp | 14358,44 | 2 |
| 551 | KIAPNMCA_04141 | Phosphatidylserine decarboxylase proenzyme | 31244,48 | 2 |
| 552 | KIAPNMCA_04028 | HTH-type transcriptional regulator BetI | 19338,42 | 2 |
| 553 | KIAPNMCA_02916 | Imidazolonepropionase | 12486,45 | 2 |

| | | | | |
|---|---|---|---|---|
| 554 | KIAPNMCA_04149 | Regulator of ribonuclease activity B | 14219,57 | 2 |
| 555 | KIAPNMCA_04022 | Outer membrane protein assembly factor BamE | 13146,78 | 2 |
| 556 | KIAPNMCA_00919 | Aminopeptidase YwaD | 39420,41 | 2 |
| 557 | KIAPNMCA_04416 | Aconitate hydratase B | 21460 | 2 |
| 558 | KIAPNMCA_01571 | hypothetical protein | 24438,98 | 2 |
| 559 | KIAPNMCA_03870 | Methionine aminopeptidase | 25966,25 | 2 |
| 560 | KIAPNMCA_03951 | Sigma-E factor regulatory protein RseB | 35237,1 | 2 |
| 561 | KIAPNMCA_03955 | ECF RNA polymerase sigma-E factor | 9202,64 | 2 |
| 562 | KIAPNMCA_01159 | D-xylose 1-dehydrogenase | 12898,67 | 2 |
| 563 | KIAPNMCA_05158 | Small-conductance mechanosensitive channel | 29643,04 | 2 |
| 564 | KIAPNMCA_01271 | Polyphosphate kinase | 43770,45 | 2 |
| 565 | KIAPNMCA_01277 | hypothetical protein | 28934,14 | 2 |
| 566 | KIAPNMCA_01071 | Biodegradative arginine decarboxylase | 18807,35 | 2 |
| 567 | KIAPNMCA_03823 | hypothetical protein | 9022,53 | 2 |
| 568 | KIAPNMCA_01195 | Ornithine carbamoyltransferase | 25952,77 | 2 |
| 569 | KIAPNMCA_02880 | Cysteine--tRNA ligase | 21937,33 | 2 |
| 570 | KIAPNMCA_01613 | RNase adapter protein RapZ | 24852,02 | 2 |
| 571 | KIAPNMCA_04185 | 30S ribosomal protein S21 | 5261,78 | 2 |
| 572 | KIAPNMCA_05354 | ATP synthase gamma chain | 14022,09 | 2 |
| 573 | KIAPNMCA_02953 | Riboflavin biosynthesis protein RibBA | 40593,9 | 2 |
| 574 | KIAPNMCA_02951 | Transcription antitermination protein NusB | 15782,52 | 2 |
| 575 | KIAPNMCA_05623 | Fe/S biogenesis protein NfuA | 21200,61 | 2 |
| 576 | KIAPNMCA_03164 | D-inositol-3-phosphate glycosyltransferase | 45022,65 | 2 |
| 577 | KIAPNMCA_04113 | 30S ribosomal protein S18 | 9398,97 | 2 |
| 578 | KIAPNMCA_05136 | Alpha,alpha-trehalose phosphorylase | 32849,45 | 2 |
| 579 | KIAPNMCA_03915 | RNA polymerase sigma factor RpoS | 37748,71 | 2 |
| 580 | KIAPNMCA_03898 | Xanthine phosphoribosyltransferase | 17566,95 | 2 |
| 581 | KIAPNMCA_00138 | Long-chain-fatty-acid--CoA ligase | 27768,41 | 2 |
| 582 | KIAPNMCA_02545 | Electron transfer flavoprotein subunit beta | 26771,62 | 2 |

| | | | | |
|---|---|---|---|---|
| 583 | KIAPNMCA_00331 | UDP-glucose 4-epimerase | 36975,56 | 2 |
| 584 | KIAPNMCA_04960 | Protein translocase subunit SecY | 30915,25 | 2 |
| 585 | KIAPNMCA_02176 | hypothetical protein | 22791,26 | 2 |
| 586 | KIAPNMCA_01079 | 4-hydroxy-3-methylbut-2-en-1-yl diphosphate synthase (flavodoxin) | 33732,85 | 2 |
| 587 | KIAPNMCA_04623 | Biotin carboxyl carrier protein of acetyl-CoA carboxylase | 16216,27 | 2 |
| 588 | KIAPNMCA_04908 | Metalloprotease PmbA | 48121,21 | 2 |
| 589 | KIAPNMCA_01362 | hypothetical protein | 52502,31 | 2 |
| 590 | KIAPNMCA_01667 | Protease 3 | 63151,16 | 2 |
| 591 | KIAPNMCA_00102 | Glycine cleavage system H protein | 13930,7 | 2 |
| 592 | KIAPNMCA_01591 | Cell division protein ZapE | 42462,59 | 2 |
| 593 | KIAPNMCA_00717 | FAD:protein FMN transferase | 37442,37 | 2 |
| 594 | KIAPNMCA_00719 | Na(+)-translocating NADH-quinone reductase subunit F | 9597,72 | 2 |
| 595 | KIAPNMCA_05401 | hypothetical protein | 11464,96 | 2 |
| 596 | KIAPNMCA_03755 | Phosphoribosylformylglycinamidine cyclo-ligase | 22891,43 | 2 |
| 597 | KIAPNMCA_04275 | hypothetical protein | 37616,75 | 2 |
| 598 | KIAPNMCA_04277 | Succinate-semialdehyde dehydrogenase [NADP(+)] GabD | 35506,37 | 2 |
| 599 | KIAPNMCA_03943 | Pyridoxine 5'-phosphate synthase | 26545,83 | 2 |
| 600 | KIAPNMCA_00899 | N-carbamoyl-D-amino acid hydrolase | 31514,6 | 2 |
| 601 | KIAPNMCA_04574 | Vitamin B12 transporter BtuB | 24910,82 | 2 |
| 602 | KIAPNMCA_05719 | Vitamin B12 transporter BtuB | 14558,47 | 2 |
| 603 | KIAPNMCA_01611 | Ribosome hibernation promoting factor | 11016,87 | 2 |
| 604 | KIAPNMCA_02207 | DNA translocase FtsK | 83547,6 | 2 |
| 605 | KIAPNMCA_01599 | Intermembrane phospholipid transport system binding protein MlaD | 16355,72 | 2 |
| 606 | KIAPNMCA_03865 | Uridylate kinase | 15112,84 | 2 |
| 607 | KIAPNMCA_01181 | Shikimate kinase 1 | 19409,07 | 2 |
| 608 | KIAPNMCA_00610 | Ribosomal silencing factor RsfS | 11838,08 | 2 |
| 609 | KIAPNMCA_05341 | Bifunctional protein GlmU | 20860,4 | 2 |
| 610 | KIAPNMCA_05340 | Bifunctional protein GlmU | 28854,7 | 2 |
| 611 | KIAPNMCA_02105 | 4-hydroxy-tetrahydrodipicolinate synthase | 31434,62 | 2 |

| | | | | |
|---|---|---|---|---|
| 612 | KIAPNMCA_02981 | Maleylpyruvate isomerase | 23994,29 | 2 |
| 613 | KIAPNMCA_01135 | Arginine N-succinyltransferase | 38037,26 | 2 |
| 614 | KIAPNMCA_03016 | Catalase-peroxidase | 14894,52 | 2 |
| 615 | KIAPNMCA_03150 | Lipoprotein-releasing system ATP-binding protein LolD | 25048,39 | 2 |
| 616 | KIAPNMCA_03158 | hypothetical protein | 37079,34 | 2 |
| 617 | KIAPNMCA_02183 | D-erythrose-4-phosphate dehydrogenase | 14971,87 | 2 |
| 618 | KIAPNMCA_05459 | DNA helicase II | 44305,69 | 2 |
| 619 | KIAPNMCA_05458 | DNA helicase II | 38916,52 | 2 |
| 620 | KIAPNMCA_05398 | Glycine--tRNA ligase alpha subunit | 35581,55 | 2 |
| 621 | KIAPNMCA_03975 | hypothetical protein | 15059,41 | 2 |
| 622 | KIAPNMCA_04222 | Tol-Pal system protein TolB | 24671,57 | 2 |
| 623 | KIAPNMCA_04003 | Oxaloacetate decarboxylase alpha chain | 56134,96 | 2 |
| 624 | KIAPNMCA_02873 | HTH-type transcriptional regulator CysB | 32222,82 | 2 |
| 625 | KIAPNMCA_01253 | 2,3-bisphosphoglycerate-independent phosphoglycerate mutase | 34460,28 | 2 |
| 626 | KIAPNMCA_02471 | Phthiocerol synthesis polyketide synthase type I PpsE | 74480,53 | 2 |
| 627 | KIAPNMCA_02335 | putative GST-like protein YibF | 22004,41 | 2 |
| 628 | KIAPNMCA_04094 | Translational regulator CsrA | 7334,8 | 2 |
| 629 | KIAPNMCA_01327 | hypothetical protein | 23624,56 | 2 |
| 630 | KIAPNMCA_01416 | Cell division protein FtsA | 13801,13 | 2 |
| 631 | KIAPNMCA_01179 | Vitamin B12 import ATP-binding protein BtuD | 47099,1 | 2 |
| 632 | KIAPNMCA_02584 | hypothetical protein | 19259,09 | 2 |
| 633 | KIAPNMCA_05420 | Xaa-Pro dipeptidase | 15671,94 | 2 |
| 634 | KIAPNMCA_02513 | Tail-specific protease | 75716,22 | 2 |
| 635 | KIAPNMCA_04924 | Ribonuclease G | 20671 | 2 |
| 636 | KIAPNMCA_01881 | 3-oxoacyl-[acyl-carrier-protein] reductase FabG | 5902,16 | 2 |
| 637 | KIAPNMCA_03839 | Acetyl-coenzyme A carboxylase carboxyl transferase subunit alpha | 20839,55 | 2 |
| 638 | KIAPNMCA_05704 | Neutral endopeptidase | 7677,9 | 2 |
| 639 | KIAPNMCA_05894 | hypothetical protein | 6493,54 | 2 |
| 640 | KIAPNMCA_02520 | 2-oxoisovalerate dehydrogenase subunit beta | 15208,8 | 2 |

| | | | | |
|---|---|---|---|---|
| 641 | KIAPNMCA_02606 | High frequency lysogenization protein HflD | 23033,72 | 2 |
| 642 | KIAPNMCA_02565 | Asparagine synthetase B [glutamine-hydrolyzing] | 14289,28 | 2 |
| 643 | KIAPNMCA_01420 | UDP-N-acetylmuramate--L-alanine ligase | 15798,16 | 2 |
| 644 | KIAPNMCA_01421 | UDP-N-acetylmuramate--L-alanine ligase | 29486,02 | 2 |
| 645 | KIAPNMCA_01056 | putative ABC transporter ATP-binding protein YknY | 23352,98 | 2 |
| 646 | KIAPNMCA_02519 | Dihydrolipoyllysine-residue acetyltransferase component of pyruvate dehydrogenase complex | 26230,59 | 2 |
| 647 | KIAPNMCA_05027 | hypothetical protein | 14246 | 2 |
| 648 | KIAPNMCA_03188 | Beta-barrel assembly-enhancing protease | 30436,86 | 2 |
| 649 | KIAPNMCA_00054 | hypothetical protein | 18278,12 | 2 |
| 650 | KIAPNMCA_03333 | UDP-Gal:alpha-D-GlcNAc-diphosphoundecaprenol beta-1,4-galactosyltransferase | 27820,28 | 2 |
| 651 | KIAPNMCA_04726 | Vitamin B12 import ATP-binding protein BtuD | 32580,21 | 2 |
| 652 | KIAPNMCA_01517 | hypothetical protein | 37634,32 | 2 |
| 653 | KIAPNMCA_04722 | hypothetical protein | 7263,74 | 2 |
| 654 | KIAPNMCA_03186 | UDP-N-acetyl-D-mannosamine dehydrogenase | 11894,34 | 2 |
| 655 | KIAPNMCA_01124 | hypothetical protein | 10418,24 | 2 |
| 656 | KIAPNMCA_03142 | Virulence transcriptional regulatory protein PhoP | 11032,89 | 2 |
| 657 | KIAPNMCA_02416 | Protease HtpX | 28967,32 | 2 |
| 658 | KIAPNMCA_03804 | hypothetical protein | 54029,26 | 2 |
| 659 | KIAPNMCA_00908 | Carbamoyl-phosphate synthase small chain | 40737,83 | 2 |
| 660 | KIAPNMCA_05749 | hypothetical protein | 17623,95 | 2 |
| 661 | KIAPNMCA_03732 | hypothetical protein | 33939,61 | 2 |
| 662 | KIAPNMCA_03960 | tRNA-modifying protein YgfZ | 36210,55 | 2 |
| 663 | KIAPNMCA_00230 | hypothetical protein | 29253,91 | 2 |
| 664 | KIAPNMCA_00785 | Peptide chain release factor RF1 | 41113,65 | 2 |
| 665 | KIAPNMCA_03961 | hypothetical protein | 19368,74 | 2 |
| 666 | KIAPNMCA_03963 | hypothetical protein | 11127,72 | 2 |
| 667 | KIAPNMCA_01206 | 50S ribosomal protein L31 | 8074,06 | 2 |

| | | | | |
|---|---|---|---|---|
| 668 | KIAPNMCA_02165 | Glutamate-pyruvate aminotransferase AlaA | 11165,62 | 2 |
| 669 | KIAPNMCA_02438 | hypothetical protein | 19303,84 | 2 |
| 670 | KIAPNMCA_01392 | hypothetical protein | 26205,24 | 2 |
| 671 | KIAPNMCA_04690 | Orotate phosphoribosyltransferase | 23558,2 | 2 |
| 672 | KIAPNMCA_04915 | Metalloprotease TldD | 51480,88 | 2 |
| 673 | KIAPNMCA_00644 | hypothetical protein | 4508,51 | 2 |
| 674 | KIAPNMCA_00646 | 30S ribosomal protein S15 | 10022,39 | 2 |
| 675 | KIAPNMCA_00161 | hypothetical protein | 10435,34 | 2 |
| 676 | KIAPNMCA_03363 | Translation initiation factor IF-3 | 12384,69 | 2 |
| 677 | KIAPNMCA_05924 | 3-beta-hydroxycholanate 3-dehydrogenase (NAD(+)) 1 | 26765,82 | 2 |
| 678 | KIAPNMCA_01468 | Signal recognition particle receptor FtsY | 32531,24 | 2 |
| 679 | KIAPNMCA_01461 | S-adenosylmethionine:tRNA ribosyltransferase-isomerase | 38248,81 | 2 |
| 680 | KIAPNMCA_02898 | GTP cyclohydrolase 1 | 24081,45 | 2 |
| 681 | KIAPNMCA_02899 | hypothetical protein | 13502,26 | 2 |
| 682 | KIAPNMCA_01829 | Succinate dehydrogenase cytochrome b556 subunit | 14030,64 | 2 |
| 683 | KIAPNMCA_02296 | Pyruvate kinase II | 46060,99 | 2 |
| 684 | KIAPNMCA_05953 | Soluble pyridine nucleotide transhydrogenase | 23475,31 | 2 |
| 685 | KIAPNMCA_02095 | NAD(P)H dehydrogenase (quinone) | 20444,54 | 2 |
| 686 | KIAPNMCA_00959 | RNA polymerase-binding transcription factor DksA | 17177,78 | 2 |
| 687 | KIAPNMCA_03087 | UDP-2-acetamido-2-deoxy-3-oxo-D-glucuronate aminotransferase | 32568,28 | 2 |
| 688 | KIAPNMCA_03085 | hypothetical protein | 19019,78 | 2 |
| 689 | KIAPNMCA_02571 | Cysteine synthase A | 6527,54 | 2 |
| 690 | KIAPNMCA_05606 | Cytochrome c6 | 15707,8 | 2 |
| 691 | KIAPNMCA_05371 | ATP-dependent RNA helicase DeaD | 8722,85 | 2 |
| 692 | KIAPNMCA_01077 | Histidine--tRNA ligase | 47121,31 | 2 |
| 693 | KIAPNMCA_02855 | hypothetical protein | 23528,88 | 2 |
| 694 | KIAPNMCA_01076 | hypothetical protein | 23070,64 | 2 |
| 695 | KIAPNMCA_03365 | Threonine--tRNA ligase | 23205,04 | 2 |
| 696 | KIAPNMCA_02496 | Dihydroorotate dehydrogenase (quinone) | 28168,29 | 2 |

| | | | | |
|---|---|---|---|---|
| 697 | KIAPNMCA_01483 | Protein LemA | 21291,01 | 2 |
| 698 | KIAPNMCA_02495 | Dihydroorotate dehydrogenase (quinone) | 10367,56 | 2 |
| 699 | KIAPNMCA_03212 | Surfactin synthase subunit 3 | 47331,36 | 2 |
| 700 | KIAPNMCA_03355 | Acyl-coenzyme A dehydrogenase | 75033,48 | 2 |
| 701 | KIAPNMCA_03059 | hypothetical protein | 11671,94 | 1 |
| 702 | KIAPNMCA_01151 | Beta-galactosidase | 98771,05 | 1 |
| 703 | KIAPNMCA_01156 | HTH-type transcriptional repressor NanR | 26951,73 | 1 |
| 704 | KIAPNMCA_03034 | hypothetical protein | 10778,31 | 1 |
| 705 | KIAPNMCA_02405 | Phospho-2-dehydro-3-deoxyheptonate aldolase | 27109,8 | 1 |
| 706 | KIAPNMCA_02404 | Phospho-2-dehydro-3-deoxyheptonate aldolase AroG | 11000,42 | 1 |
| 707 | KIAPNMCA_05557 | hypothetical protein | 43496,12 | 1 |
| 708 | KIAPNMCA_03330 | hypothetical protein | 44558,03 | 1 |
| 709 | KIAPNMCA_05250 | Vitamin B12 transporter BtuB | 32270,02 | 1 |
| 710 | KIAPNMCA_02913 | hypothetical protein | 28509,48 | 1 |
| 711 | KIAPNMCA_05431 | Methionyl-tRNA formyltransferase | 17888,3 | 1 |
| 712 | KIAPNMCA_05432 | Methionyl-tRNA formyltransferase | 17954,53 | 1 |
| 713 | KIAPNMCA_00436 | Ribosomal large subunit pseudouridine synthase D | 23713,27 | 1 |
| 714 | KIAPNMCA_01158 | hypothetical protein | 9234,57 | 1 |
| 715 | KIAPNMCA_02536 | Negative modulator of initiation of replication | 17547,15 | 1 |
| 716 | KIAPNMCA_05500 | Phosphoenolpyruvate carboxylase | 16753,77 | 1 |
| 717 | KIAPNMCA_03810 | Protein-glutamate methylesterase/protein-glutamine glutaminase | 37670,69 | 1 |
| 718 | KIAPNMCA_02426 | Asparagine--tRNA ligase | 26954,5 | 1 |
| 719 | KIAPNMCA_02673 | Protein phosphatase CheZ | 27544,94 | 1 |
| 720 | KIAPNMCA_05646 | Protein YhgF | 75346,66 | 1 |
| 721 | KIAPNMCA_02078 | hypothetical protein | 12982,91 | 1 |
| 722 | KIAPNMCA_04925 | Ribonuclease G | 6418,49 | 1 |
| 723 | KIAPNMCA_03592 | Regulatory protein AtoC | 39496,31 | 1 |
| 724 | KIAPNMCA_05481 | hypothetical protein | 26759,99 | 1 |
| 725 | KIAPNMCA_05167 | GTP-binding protein EngB | 15999,3 | 1 |

| | | | | |
|---|---|---|---|---|
| 726 | KIAPNMCA_00096 | UTP--glucose-1-phosphate uridylyltransferase | 32987,29 | 1 |
| 727 | KIAPNMCA_01457 | Protein translocase subunit SecD | 8369,43 | 1 |
| 728 | KIAPNMCA_01272 | Polyphosphate kinase | 37343,77 | 1 |
| 729 | KIAPNMCA_05283 | Glutamine--fructose-6-phosphate aminotransferase [isomerizing] | 14172,61 | 1 |
| 730 | KIAPNMCA_00673 | ATP-dependent RNA helicase SrmB | 46249,44 | 1 |
| 731 | KIAPNMCA_00179 | HTH-type transcriptional repressor RspR | 24955,35 | 1 |
| 732 | KIAPNMCA_01871 | hypothetical protein | 40654,42 | 1 |
| 733 | KIAPNMCA_03645 | Dipeptidyl aminopeptidase BIII | 72695,3 | 1 |
| 734 | KIAPNMCA_01860 | Cation/acetate symporter ActP | 42488,11 | 1 |
| 735 | KIAPNMCA_00739 | hypothetical protein | 20648,7 | 1 |
| 736 | KIAPNMCA_05556 | Macrolide export ATP-binding/permease protein MacB | 17266,98 | 1 |
| 737 | KIAPNMCA_02927 | Bifunctional chorismate mutase/prephenate dehydratase | 42923,26 | 1 |
| 738 | KIAPNMCA_01192 | Argininosuccinate lyase | 47782,86 | 1 |
| 739 | KIAPNMCA_00411 | hypothetical protein | 28522,18 | 1 |
| 740 | KIAPNMCA_03104 | hypothetical protein | 14678,12 | 1 |
| 741 | KIAPNMCA_01202 | hypothetical protein | 48669,43 | 1 |
| 742 | KIAPNMCA_00269 | hypothetical protein | 21074,82 | 1 |
| 743 | KIAPNMCA_01200 | N-acetyl-gamma-glutamyl-phosphate reductase | 28358,73 | 1 |
| 744 | KIAPNMCA_01201 | Bifunctional aspartokinase/homoserine dehydrogenase 2 | 37257,27 | 1 |
| 745 | KIAPNMCA_02442 | Ribosomal RNA large subunit methyltransferase K/L | 80309,97 | 1 |
| 746 | KIAPNMCA_02599 | ATP-dependent Clp protease ATP-binding subunit ClpA | 24033,05 | 1 |
| 747 | KIAPNMCA_05948 | Regulator of sigma D | 16000,89 | 1 |
| 748 | KIAPNMCA_04069 | hypothetical protein | 15023,6 | 1 |
| 749 | KIAPNMCA_02543 | L-amino acid N-acetyltransferase AaaT | 18334,26 | 1 |
| 750 | KIAPNMCA_05943 | putative ABC transporter ATP-binding protein YheS | 71106,9 | 1 |
| 751 | KIAPNMCA_02085 | hypothetical protein | 24938,98 | 1 |
| 752 | KIAPNMCA_03764 | hypothetical protein | 17552,87 | 1 |
| 753 | KIAPNMCA_01873 | Ribosomal large subunit pseudouridine synthase C | 32189,58 | 1 |
| 754 | KIAPNMCA_05657 | Type II secretion system protein G | 16002,18 | 1 |

| | | | | |
|---|---|---|---|---|
| 755 | KIAPNMCA_03160 | Iron-sulfur cluster carrier protein | 32141,97 | 1 |
| 756 | KIAPNMCA_03163 | hypothetical protein | 53486,54 | 1 |
| 757 | KIAPNMCA_02881 | Cysteine--tRNA ligase | 30199,86 | 1 |
| 758 | KIAPNMCA_02379 | Ribonucleoside-diphosphate reductase 1 subunit beta | 32409,21 | 1 |
| 759 | KIAPNMCA_01869 | hypothetical protein | 6177,18 | 1 |
| 760 | KIAPNMCA_04117 | Ribonuclease R | 18849,87 | 1 |
| 761 | KIAPNMCA_04111 | Replicative DNA helicase | 31985,07 | 1 |
| 762 | KIAPNMCA_04110 | Replicative DNA helicase | 20553,63 | 1 |
| 763 | KIAPNMCA_00453 | Arginine--tRNA ligase | 64324,03 | 1 |
| 764 | KIAPNMCA_02303 | KHG/KDPG aldolase | 22890,09 | 1 |
| 765 | KIAPNMCA_05767 | hypothetical protein | 21153,6 | 1 |
| 766 | KIAPNMCA_05766 | hypothetical protein | 51456,27 | 1 |
| 767 | KIAPNMCA_03581 | hypothetical protein | 7675,85 | 1 |
| 768 | KIAPNMCA_03090 | UDP-N-acetyl-2-amino-2-deoxy-D-glucuronate oxidase | 16346,48 | 1 |
| 769 | KIAPNMCA_00358 | hypothetical protein | 65424,04 | 1 |
| 770 | KIAPNMCA_00130 | Septum site-determining protein MinC | 17777,07 | 1 |
| 771 | KIAPNMCA_02109 | Phosphoribosylaminoimidazole-succinocarboxamide synthase | 12097,04 | 1 |
| 772 | KIAPNMCA_05616 | hypothetical protein | 19715,19 | 1 |
| 773 | KIAPNMCA_02100 | Aldose sugar dehydrogenase YliI | 40777,02 | 1 |
| 774 | KIAPNMCA_02102 | Peroxiredoxin Bcp | 14398,51 | 1 |
| 775 | KIAPNMCA_03784 | hypothetical protein | 6849,29 | 1 |
| 776 | KIAPNMCA_02860 | hypothetical protein | 29019,63 | 1 |
| 777 | KIAPNMCA_00158 | hypothetical protein | 9586,89 | 1 |
| 778 | KIAPNMCA_02845 | hypothetical protein | 19905,25 | 1 |
| 779 | KIAPNMCA_02847 | Phosphatidylglycerol--prolipoprotein diacylglyceryl transferase | 22895,35 | 1 |
| 780 | KIAPNMCA_02846 | Thymidylate synthase 2 | 31566,9 | 1 |
| 781 | KIAPNMCA_02843 | hypothetical protein | 31189,32 | 1 |
| 782 | KIAPNMCA_02849 | Phosphoenolpyruvate-dependent phosphotransferase system | 84067,63 | 1 |
| 783 | KIAPNMCA_01494 | L-threonine 3-dehydrogenase | 37227,7 | 1 |

| | | | | |
|---|---|---|---|---|
| 784 | KIAPNMCA_01320 | Phosphate regulon transcriptional regulatory protein PhoB | 7228,79 | 1 |
| 785 | KIAPNMCA_03267 | hypothetical protein | 21143,82 | 1 |
| 786 | KIAPNMCA_03264 | 2,3-dehydroadipyl-CoA hydratase | 29027,03 | 1 |
| 787 | KIAPNMCA_01911 | IS66 family transposase ISSpu21 | 10287,66 | 1 |
| 788 | KIAPNMCA_05099 | hypothetical protein | 20083,33 | 1 |
| 789 | KIAPNMCA_05516 | hypothetical protein | 76134,38 | 1 |
| 790 | KIAPNMCA_04745 | hypothetical protein | 8266,05 | 1 |
| 791 | KIAPNMCA_04150 | 1-acyl-sn-glycerol-3-phosphate acyltransferase | 27194,19 | 1 |
| 792 | KIAPNMCA_05839 | hypothetical protein | 17029,54 | 1 |
| 793 | KIAPNMCA_01596 | Acid stress protein IbaG | 9680,04 | 1 |
| 794 | KIAPNMCA_05391 | 50S ribosomal protein L34 | 5317,15 | 1 |
| 795 | KIAPNMCA_00712 | Recombination-associated protein RdgC | 33866,31 | 1 |
| 796 | KIAPNMCA_04784 | hypothetical protein | 52855,43 | 1 |
| 797 | KIAPNMCA_00139 | Long-chain-fatty-acid--CoA ligase | 28260,89 | 1 |
| 798 | KIAPNMCA_05405 | hypothetical protein | 27269,44 | 1 |
| 799 | KIAPNMCA_04404 | 50S ribosomal protein L27 | 5024,74 | 1 |
| 800 | KIAPNMCA_00844 | hypothetical protein | 23282,27 | 1 |
| 801 | KIAPNMCA_00929 | hypothetical protein | 42411,24 | 1 |
| 802 | KIAPNMCA_03138 | Osmoregulated proline transporter OpuE | 45676,72 | 1 |
| 803 | KIAPNMCA_03074 | Asparagine synthetase [glutamine-hydrolyzing] 1 | 17046,61 | 1 |
| 804 | KIAPNMCA_03198 | hypothetical protein | 18376,09 | 1 |
| 805 | KIAPNMCA_02509 | Aminopeptidase N | 70853,96 | 1 |
| 806 | KIAPNMCA_02505 | hypothetical protein | 42246,05 | 1 |
| 807 | KIAPNMCA_03197 | hypothetical protein | 29084,48 | 1 |
| 808 | KIAPNMCA_03058 | ATP-dependent RNA helicase RhlE | 39838,89 | 1 |
| 809 | KIAPNMCA_02502 | Ycf48-like protein | 28660,83 | 1 |
| 810 | KIAPNMCA_03072 | Imidazole glycerol phosphate synthase subunit HisF | 27746,65 | 1 |
| 811 | KIAPNMCA_01748 | Vitamin B12 transporter BtuB | 67842,83 | 1 |
| 812 | KIAPNMCA_03947 | Ribonuclease 3 | 24872 | 1 |

| | | | | |
|---|---|---|---|---|
| 813 | KIAPNMCA_05651 | 33 kDa chaperonin | 26512,56 | 1 |
| 814 | KIAPNMCA_04573 | hypothetical protein | 84105,01 | 1 |
| 815 | KIAPNMCA_05715 | hypothetical protein | 29276,01 | 1 |
| 816 | KIAPNMCA_02074 | hypothetical protein | 29581,44 | 1 |
| 817 | KIAPNMCA_05301 | Serine/threonine transporter SstT | 24702,64 | 1 |
| 818 | KIAPNMCA_00669 | Single-stranded-DNA-specific exonuclease RecJ | 47072,62 | 1 |
| 819 | KIAPNMCA_03149 | hypothetical protein | 91642,48 | 1 |
| 820 | KIAPNMCA_00662 | RNA-binding protein YhbY | 11027,28 | 1 |
| 821 | KIAPNMCA_04395 | 4-hydroxythreonine-4-phosphate dehydrogenase | 35421,78 | 1 |
| 822 | KIAPNMCA_01444 | hypothetical protein | 19988,56 | 1 |
| 823 | KIAPNMCA_05190 | Chromosome partition protein Smc | 141141,39 | 1 |
| 824 | KIAPNMCA_00425 | Signal recognition particle protein | 21891,84 | 1 |
| 825 | KIAPNMCA_04580 | hypothetical protein | 26900,19 | 1 |
| 826 | KIAPNMCA_03475 | putative protein | 16836,03 | 1 |
| 827 | KIAPNMCA_04582 | Inosose dehydratase | 37751,94 | 1 |
| 828 | KIAPNMCA_01440 | Penicillin-binding protein activator LpoA | 59739,73 | 1 |
| 829 | KIAPNMCA_03678 | hypothetical protein | 23377,77 | 1 |
| 830 | KIAPNMCA_03803 | hypothetical protein | 13019,31 | 1 |
| 831 | KIAPNMCA_04947 | Type 3 secretion system secretin | 52873,11 | 1 |
| 832 | KIAPNMCA_01624 | Dipeptidyl aminopeptidase BI | 23290,59 | 1 |
| 833 | KIAPNMCA_00259 | Efflux pump periplasmic linker BepF | 38317,59 | 1 |
| 834 | KIAPNMCA_01623 | Dipeptidyl aminopeptidase BI | 57481,9 | 1 |
| 835 | KIAPNMCA_01216 | Regulator of ribonuclease activity A | 17238,42 | 1 |
| 836 | KIAPNMCA_02372 | Phosphoserine aminotransferase | 7077,53 | 1 |
| 837 | KIAPNMCA_03237 | hypothetical protein | 10600,17 | 1 |
| 838 | KIAPNMCA_00582 | hypothetical protein | 17758,23 | 1 |
| 839 | KIAPNMCA_02278 | hypothetical protein | 28101,72 | 1 |
| 840 | KIAPNMCA_05441 | N5-carboxyaminoimidazole ribonucleotide mutase | 9178,04 | 1 |
| 841 | KIAPNMCA_04758 | 2-oxopent-4-enoate hydratase | 28481,73 | 1 |

| | | | | |
|---|---|---|---|---|
| 842 | KIAPNMCA_03133 | hypothetical protein | 46311,22 | 1 |
| 843 | KIAPNMCA_00069 | hypothetical protein | 27390,65 | 1 |
| 844 | KIAPNMCA_01691 | Bacterioferritin | 18485,44 | 1 |
| 845 | KIAPNMCA_01690 | Bacterioferritin | 18219,06 | 1 |
| 846 | KIAPNMCA_01694 | hypothetical protein | 11723,66 | 1 |
| 847 | KIAPNMCA_00804 | hypothetical protein | 8736,36 | 1 |
| 848 | KIAPNMCA_04728 | hypothetical protein | 17611,66 | 1 |
| 849 | KIAPNMCA_04686 | Deoxyuridine 5'-triphosphate nucleotidohydrolase | 16380,36 | 1 |
| 850 | KIAPNMCA_04712 | hypothetical protein | 34863,07 | 1 |
| 851 | KIAPNMCA_03159 | hypothetical protein | 61813,67 | 1 |
| 852 | KIAPNMCA_05674 | Inner membrane protein YqiK | 47982,54 | 1 |
| 853 | KIAPNMCA_02381 | Protein YceI | 20380,51 | 1 |
| 854 | KIAPNMCA_02719 | hypothetical protein | 8810,47 | 1 |
| 855 | KIAPNMCA_05125 | UvrABC system protein A | 83001,66 | 1 |
| 856 | KIAPNMCA_00727 | Na(+)-translocating NADH-quinone reductase subunit A | 9379,59 | 1 |
| 857 | KIAPNMCA_00725 | Na(+)-translocating NADH-quinone reductase subunit B | 15225,01 | 1 |
| 858 | KIAPNMCA_03057 | hypothetical protein | 6495,58 | 1 |
| 859 | KIAPNMCA_00626 | Adaptive-response sensory-kinase SasA | 71044,17 | 1 |
| 860 | KIAPNMCA_02655 | Cytochrome c-type biogenesis protein CcmE | 7996,53 | 1 |
| 861 | KIAPNMCA_03805 | hypothetical protein | 6879,51 | 1 |
| 862 | KIAPNMCA_05671 | hypothetical protein | 23943,4 | 1 |
| 863 | KIAPNMCA_03990 | Multidrug resistance protein MdtC | 116436,88 | 1 |
| 864 | KIAPNMCA_03991 | Multidrug resistance protein MdtA | 41532,1 | 1 |
| 865 | KIAPNMCA_00203 | Asp/Glu-specific dipeptidyl-peptidase | 8455,15 | 1 |
| 866 | KIAPNMCA_04380 | hypothetical protein | 52395,92 | 1 |
| 867 | KIAPNMCA_03974 | hypothetical protein | 7732,08 | 1 |
| 868 | KIAPNMCA_04223 | Dipeptidyl-peptidase 5 | 31172,5 | 1 |
| 869 | KIAPNMCA_05589 | hypothetical protein | 18785,45 | 1 |

| | | | | |
|---|---|---|---|---|
| 870 | KIAPNMCA_00980 | UDP-N-acetylmuramate--L-alanyl-gamma-D-glutamyl-meso-2,6-diaminoheptandioate ligase | 49380,11 | 1 |
| 871 | KIAPNMCA_00981 | Fructose-1,6-bisphosphatase class 1 | 10014,15 | 1 |
| 872 | KIAPNMCA_00984 | hypothetical protein | 84152,22 | 1 |
| 873 | KIAPNMCA_00988 | hypothetical protein | 25847,24 | 1 |
| 874 | KIAPNMCA_02370 | 3-phosphoshikimate 1-carboxyvinyltransferase | 46379,83 | 1 |
| 875 | KIAPNMCA_00091 | hypothetical protein | 61426,11 | 1 |
| 876 | KIAPNMCA_00319 | Carbonic anhydrase 2 | 19647 | 1 |
| 877 | KIAPNMCA_02877 | DNA topoisomerase 1 | 97693,81 | 1 |
| 878 | KIAPNMCA_02878 | N-succinylarginine dihydrolase | 37036,48 | 1 |
| 879 | KIAPNMCA_02332 | Methionine--tRNA ligase | 28975,9 | 1 |
| 880 | KIAPNMCA_00619 | D-alanyl-D-alanine carboxypeptidase DacC | 6894,7 | 1 |
| 881 | KIAPNMCA_03275 | putative succinyl-CoA:3-ketoacid coenzyme A transferase subunit B | 12967,46 | 1 |
| 882 | KIAPNMCA_04346 | hypothetical protein | 17672,56 | 1 |
| 883 | KIAPNMCA_04356 | Multidrug resistance protein MexB | 118525,11 | 1 |
| 884 | KIAPNMCA_01174 | Tryptophan--tRNA ligase | 20671,44 | 1 |
| 885 | KIAPNMCA_02777 | Chromosome partition protein Smc | 21945,89 | 1 |
| 886 | KIAPNMCA_01322 | Phosphate regulon transcriptional regulatory protein PhoB | 11349,81 | 1 |
| 887 | KIAPNMCA_03110 | Chorismate synthase | 39739,42 | 1 |
| 888 | KIAPNMCA_01672 | hypothetical protein | 8882,64 | 1 |
| 889 | KIAPNMCA_01671 | Vitamin B12 transporter BtuB | 16686,58 | 1 |
| 890 | KIAPNMCA_01670 | hypothetical protein | 28230,83 | 1 |
| 891 | KIAPNMCA_04994 | 30S ribosomal protein S12 | 13960,57 | 1 |
| 892 | KIAPNMCA_01830 | Succinate dehydrogenase hydrophobic membrane anchor subunit | 13187,07 | 1 |
| 893 | KIAPNMCA_02677 | Iron-sulfur cluster carrier protein | 24592,92 | 1 |
| 894 | KIAPNMCA_05530 | hypothetical protein | 13465,88 | 1 |
| 895 | KIAPNMCA_02675 | Chemotaxis protein CheY | 7895,92 | 1 |
| 896 | KIAPNMCA_05416 | Fatty acid oxidation complex subunit alpha | 77027,84 | 1 |

| | | | | |
|---|---|---|---|---|
| 897 | KIAPNMCA_05415 | 3-ketoacyl-CoA thiolase | 41022,67 | 1 |
| 898 | KIAPNMCA_03348 | hypothetical protein | 7087,63 | 1 |
| 899 | KIAPNMCA_03065 | Putative asparagine synthetase [glutamine-hydrolyzing] | 60862,07 | 1 |
| 900 | KIAPNMCA_03066 | Asparagine synthetase [glutamine-hydrolyzing] 1 | 11917,94 | 1 |
| 901 | KIAPNMCA_04727 | hypothetical protein | 26434,63 | 1 |
| 902 | KIAPNMCA_05387 | Membrane protein insertase YidC | 33358,3 | 1 |
| 903 | KIAPNMCA_00260 | Efflux pump membrane transporter BepE | 15883,14 | 1 |
| 904 | KIAPNMCA_01882 | 3-oxoacyl-[acyl-carrier-protein] reductase FabG | 17334,88 | 1 |
| 905 | KIAPNMCA_05597 | Diguanylate cyclase VdcA | 28559,96 | 1 |
| 906 | KIAPNMCA_00657 | Triosephosphate isomerase | 26875,74 | 1 |
| 907 | KIAPNMCA_05700 | Putative acid--amine ligase YjfC | 43693,77 | 1 |
| 908 | KIAPNMCA_05462 | hypothetical protein | 43167,55 | 1 |
| 909 | KIAPNMCA_05892 | hypothetical protein | 15978,54 | 1 |
| 910 | KIAPNMCA_02521 | 2-oxoisovalerate dehydrogenase subunit beta | 22209,14 | 1 |
| 911 | KIAPNMCA_03969 | Phosphoribosylformylglycinamidine synthase | 28395,94 | 1 |
| 912 | KIAPNMCA_01057 | hypothetical protein | 30527,98 | 1 |
| 913 | KIAPNMCA_01055 | hypothetical protein | 23607,61 | 1 |
| 914 | KIAPNMCA_01054 | hypothetical protein | 15650,44 | 1 |
| 915 | KIAPNMCA_05664 | Type II secretion system protein M | 18011,57 | 1 |
| 916 | KIAPNMCA_03336 | D-inositol-3-phosphate glycosyltransferase | 42764,47 | 1 |
| 917 | KIAPNMCA_05180 | Sulfite reductase [NADPH] flavoprotein alpha-component | 8489,35 | 1 |
| 918 | KIAPNMCA_01379 | hypothetical protein | 8208,11 | 1 |
| 919 | KIAPNMCA_02127 | hypothetical protein | 4582,29 | 1 |
| 920 | KIAPNMCA_01453 | Protein translocase subunit SecF | 33617 | 1 |
| 921 | KIAPNMCA_01428 | UDP-N-acetylmuramoyl-L-alanyl-D-glutamate--2,6-diaminopimelate ligase | 41260,97 | 1 |
| 922 | KIAPNMCA_01512 | Flavin-dependent tryptophan halogenase PrnA | 36697,89 | 1 |
| 923 | KIAPNMCA_00200 | Dipeptidyl aminopeptidase BII | 8331,3 | 1 |
| 924 | KIAPNMCA_01515 | hypothetical protein | 8508,03 | 1 |

| | | | | |
|---|---|---|---|---|
| 925 | KIAPNMCA_02900 | Energy-dependent translational throttle protein EttA | 10401,22 | 1 |
| 926 | KIAPNMCA_02901 | Energy-dependent translational throttle protein EttA | 42146,34 | 1 |
| 927 | KIAPNMCA_00123 | Cell division protein ZipA | 31159,71 | 1 |
| 928 | KIAPNMCA_01630 | Vitamin B12 transporter BtuB | 32478,33 | 1 |
| 929 | KIAPNMCA_01733 | Vitamin B12 transporter BtuB | 72497,69 | 1 |
| 930 | KIAPNMCA_01880 | Malonyl CoA-acyl carrier protein transacylase | 32734,03 | 1 |
| 931 | KIAPNMCA_04721 | Glutathione amide-dependent peroxidase | 9570,83 | 1 |
| 932 | KIAPNMCA_03849 | Acyl-[acyl-carrier-protein]--UDP-N-acetylglucosamine O-acyltransferase | 15900,11 | 1 |
| 933 | KIAPNMCA_02556 | ABC transporter permease protein NatB | 44496,85 | 1 |
| 934 | KIAPNMCA_03202 | hypothetical protein | 18920,7 | 1 |
| 935 | KIAPNMCA_04046 | Chemotaxis protein CheV | 33702,69 | 1 |
| 936 | KIAPNMCA_01046 | ATP-dependent RNA helicase RhlE | 46407,01 | 1 |
| 937 | KIAPNMCA_04326 | hypothetical protein | 12805,67 | 1 |
| 938 | KIAPNMCA_04327 | 1-deoxy-11-beta-hydroxypentalenate dehydrogenase | 25901,28 | 1 |
| 939 | KIAPNMCA_01251 | Glutaredoxin 3 | 9670,84 | 1 |
| 940 | KIAPNMCA_04169 | hypothetical protein | 22603,62 | 1 |
| 941 | KIAPNMCA_02992 | L-methionine gamma-lyase | 26576,33 | 1 |
| 942 | KIAPNMCA_02991 | L-methionine gamma-lyase | 16792,56 | 1 |
| 943 | KIAPNMCA_00437 | hypothetical protein | 9633,21 | 1 |
| 944 | KIAPNMCA_01669 | hypothetical protein | 24790,43 | 1 |
| 945 | KIAPNMCA_02688 | Flagellar motor switch protein FliN | 14287,43 | 1 |
| 946 | KIAPNMCA_03554 | Protein translocase subunit SecF | 34326,18 | 1 |
| 947 | KIAPNMCA_02395 | Putative multidrug export ATP-binding/permease protein | 59165,93 | 1 |
| 948 | KIAPNMCA_05375 | hypothetical protein | 17417,22 | 1 |
| 949 | KIAPNMCA_04131 | DNA mismatch repair protein MutL | 67009,43 | 1 |
| 950 | KIAPNMCA_00840 | hypothetical protein | 8610,36 | 1 |
| 951 | KIAPNMCA_04139 | Oligoribonuclease | 20688,47 | 1 |

| | | | | |
|---|---|---|---|---|
| 952 | KIAPNMCA_02194 | 6-phosphogluconate dehydrogenase, NADP(+)-dependent, decarboxylating | 35432,97 | 1 |
| 953 | KIAPNMCA_03121 | hypothetical protein | 13674,79 | 1 |
| 954 | KIAPNMCA_00902 | Agmatine deiminase | 12634,62 | 1 |
| 955 | KIAPNMCA_05605 | hypothetical protein | 24794,91 | 1 |
| 956 | KIAPNMCA_05748 | hypothetical protein | 30726,58 | 1 |
| 957 | KIAPNMCA_00906 | Transcription elongation factor GreA | 17331,21 | 1 |
| 958 | KIAPNMCA_00231 | hypothetical protein | 38298,53 | 1 |
| 959 | KIAPNMCA_02525 | Succinylglutamate desuccinylase | 20251,33 | 1 |
| 960 | KIAPNMCA_03252 | 3-hydroxyisobutyrate dehydrogenase | 26891,48 | 1 |
| 961 | KIAPNMCA_03746 | Chaperone protein DnaJ | 10808,57 | 1 |
| 962 | KIAPNMCA_02163 | Glutamate-pyruvate aminotransferase AlaA | 6538,45 | 1 |
| 963 | KIAPNMCA_00014 | Transcription-repair-coupling factor | 66201,91 | 1 |
| 964 | KIAPNMCA_00424 | Signal recognition particle protein | 23717,32 | 1 |
| 965 | KIAPNMCA_00422 | Ribosome maturation factor RimM | 19661,02 | 1 |
| 966 | KIAPNMCA_03251 | putative oxidoreductase | 18581,38 | 1 |
| 967 | KIAPNMCA_01199 | N-acetyl-gamma-glutamyl-phosphate reductase | 11689,94 | 1 |
| 968 | KIAPNMCA_03609 | p-aminobenzoyl-glutamate transport protein | 50323,09 | 1 |
| 969 | KIAPNMCA_04051 | hypothetical protein | 20435,16 | 1 |
| 970 | KIAPNMCA_02164 | Glutamate-pyruvate aminotransferase AlaA | 28552,75 | 1 |
| 971 | KIAPNMCA_02169 | Universal stress protein E | 34777,73 | 1 |
| 972 | KIAPNMCA_01309 | DNA-3-methyladenine glycosylase 1 | 22609,07 | 1 |
| 973 | KIAPNMCA_04691 | Ribonuclease PH | 25956,18 | 1 |
| 974 | KIAPNMCA_03908 | hypothetical protein | 4968,62 | 1 |
| 975 | KIAPNMCA_04083 | Isoleucine--tRNA ligase | 38773,3 | 1 |
| 976 | KIAPNMCA_04082 | Isoleucine--tRNA ligase | 14924,5 | 1 |
| 977 | KIAPNMCA_03361 | 50S ribosomal protein L35 | 7432,24 | 1 |
| 978 | KIAPNMCA_00558 | 5'-methylthioadenosine/S-adenosylhomocysteine nucleosidase | 25891,1 | 1 |
| 979 | KIAPNMCA_05927 | Acetolactate synthase isozyme 3 large subunit | 24507,06 | 1 |

| | | | | |
|---|---|---|---|---|
| 980 | KIAPNMCA_01610 | RNA polymerase sigma-54 factor | 20899,68 | 1 |
| 981 | KIAPNMCA_04152 | DNA topoisomerase 4 subunit A | 37027,81 | 1 |
| 982 | KIAPNMCA_03901 | Prolyl endopeptidase | 27210,65 | 1 |
| 983 | KIAPNMCA_04186 | DNA primase | 45764,9 | 1 |
| 984 | KIAPNMCA_01607 | Lipopolysaccharide export system protein LptA | 17741,98 | 1 |
| 985 | KIAPNMCA_01765 | Glycosyl hydrolase family 109 protein 1 | 15786,08 | 1 |
| 986 | KIAPNMCA_00074 | hypothetical protein | 19655,79 | 1 |
| 987 | KIAPNMCA_04591 | NAD(P)H-dependent FMN reductase | 20169,45 | 1 |
| 988 | KIAPNMCA_02014 | Ion-translocating oxidoreductase complex subunit C | 70778,5 | 1 |
| 989 | KIAPNMCA_02318 | Corrinoid adenosyltransferase | 13789,95 | 1 |
| 990 | KIAPNMCA_05364 | putative chromosome-partitioning protein ParB | 32184,1 | 1 |
| 991 | KIAPNMCA_05361 | ATP synthase subunit c | 8025,32 | 1 |
| 992 | KIAPNMCA_03254 | 2,3-dehydroadipyl-CoA hydratase | 19104,77 | 1 |
| 993 | KIAPNMCA_05362 | ATP synthase subunit a | 31624,77 | 1 |
| 994 | KIAPNMCA_02283 | hypothetical protein | 24959,3 | 1 |
| 995 | KIAPNMCA_04071 | Type IV pilus biogenesis factor PilY1 | 40837,51 | 1 |
| 996 | KIAPNMCA_05812 | Anaerobic nitric oxide reductase transcription regulator NorR | 71708,73 | 1 |
| 997 | KIAPNMCA_05951 | Uroporphyrinogen decarboxylase | 31395,95 | 1 |
| 998 | KIAPNMCA_02098 | Beta-barrel assembly-enhancing protease | 26861,89 | 1 |
| 999 | KIAPNMCA_00979 | Flavin prenyltransferase UbiX | 22873,07 | 1 |
| 1000 | KIAPNMCA_01554 | Fe(3+) ions import ATP-binding protein FbpC | 33903,8 | 1 |
| 1001 | KIAPNMCA_00976 | Aspartate 1-decarboxylase | 61525,73 | 1 |
| 1002 | KIAPNMCA_02284 | hypothetical protein | 29616,71 | 1 |
| 1003 | KIAPNMCA_03779 | Amidophosphoribosyltransferase | 15791,78 | 1 |
| 1004 | KIAPNMCA_01287 | Vitamin B12 transporter BtuB | 88816,06 | 1 |
| 1005 | KIAPNMCA_01173 | Tryptophan--tRNA ligase | 14995,7 | 1 |
| 1006 | KIAPNMCA_01629 | hypothetical protein | 46092,34 | 1 |
| 1007 | KIAPNMCA_02398 | hypothetical protein | 38482,73 | 1 |
| 1008 | KIAPNMCA_04106 | Zinc uptake regulation protein | 16525,18 | 1 |

| | | | | |
|---|---|---|---|---|
| 1009 | KIAPNMCA_04435 | Chromosome partition protein Smc | 51144,85 | 1 |
| 1010 | KIAPNMCA_05397 | DNA gyrase subunit B | 46138,92 | 1 |
| 1011 | KIAPNMCA_05100 | Nitrogenase iron protein | 26997,4 | 1 |
| 1012 | KIAPNMCA_02862 | hypothetical protein | 4116,2 | 1 |
| 1013 | KIAPNMCA_04571 | hypothetical protein | 35002,17 | 1 |
| 1014 | KIAPNMCA_03214 | Polyketide synthase PksM | 40134,75 | 1 |
| 1015 | KIAPNMCA_00298 | ATP-dependent Clp protease proteolytic subunit | 23182,75 | 1 |
| 1016 | KIAPNMCA_05390 | Ribonuclease P protein component | 14074,76 | 1 |
| 1017 | KIAPNMCA_05477 | hypothetical protein | 43526,56 | 1 |
| 1018 | KIAPNMCA_02671 | Protein-glutamate methylesterase/protein-glutamine glutaminase 1 | 40677,49 | 1 |
| 1019 | KIAPNMCA_03084 | UDP-N-acetyl-alpha-D-glucosamine C6 dehydratase | 26531,27 | 1 |
| 1020 | KIAPNMCA_00267 | hypothetical protein | 59480,24 | 1 |
| 1021 | KIAPNMCA_01436 | Transcriptional regulator MraZ | 18160,18 | 1 |
| 1022 | KIAPNMCA_01433 | Ribosomal RNA small subunit methyltransferase H | 7086,7 | 1 |
| 1023 | KIAPNMCA_00160 | hypothetical protein | 10366,89 | 1 |
| 1024 | KIAPNMCA_03858 | Regulator of sigma-E protease RseP | 38185,18 | 1 |
| 1025 | KIAPNMCA_02220 | Response regulator UvrY | 16913,74 | 1 |
| 1026 | KIAPNMCA_03856 | Outer membrane protein assembly factor BamA | 5563,77 | 1 |
| 1027 | KIAPNMCA_03851 | 3-hydroxyacyl-[acyl-carrier-protein] dehydratase FabZ | 12341,6 | 1 |
| 1028 | KIAPNMCA_03852 | UDP-3-O-(3-hydroxymyristoyl)glucosamine N-acyltransferase | 37108,13 | 1 |
| 1029 | KIAPNMCA_05604 | LexA repressor | 15353,13 | 1 |
| 1030 | KIAPNMCA_04084 | Isoleucine--tRNA ligase | 14733,57 | 1 |
| 1031 | KIAPNMCA_03995 | hypothetical protein | 11441,02 | 1 |
| 1032 | KIAPNMCA_01204 | Met repressor | 12713,56 | 1 |
| 1033 | KIAPNMCA_04224 | hypothetical protein | 14965,68 | 1 |
| 1034 | KIAPNMCA_03655 | hypothetical protein | 34779,1 | 1 |
| 1035 | KIAPNMCA_00620 | hypothetical protein | 10755,44 | 1 |
| 1036 | KIAPNMCA_02033 | Inosine-5'-monophosphate dehydrogenase | 14584,74 | 1 |
| 1037 | KIAPNMCA_04080 | FKBP-type 16 kDa peptidyl-prolyl cis-trans isomerase | 15338,69 | 1 |

| | | | | |
|---|---|---|---|---|
| 1038 | KIAPNMCA_01328 | hypothetical protein | 23904,9 | 1 |
| 1039 | KIAPNMCA_05056 | Ferrichrome outer membrane transporter/phage receptor | 77773,77 | 1 |
| 1040 | KIAPNMCA_01052 | hypothetical protein | 18076,22 | 1 |
| 1041 | KIAPNMCA_01608 | Lipopolysaccharide export system ATP-binding protein LptB | 20818,2 | 1 |
| 1042 | KIAPNMCA_03216 | hypothetical protein | 74805,03 | 1 |
| 1043 | KIAPNMCA_03353 | hypothetical protein | 26707,95 | 1 |
| 1044 | KIAPNMCA_00115 | Glutamate--tRNA ligase | 13944,39 | 1 |
| 1045 | KIAPNMCA_05697 | Amino-acid acetyltransferase | 24174,24 | 1 |
| | | | | 7487 |
